# Randomized, open-label, phase 2a study to evaluate the contribution of artefenomel to the clinical and parasiticidal activity of artefenomel plus ferroquine in African patients with uncomplicated *Plasmodium falciparum* malaria

**DOI:** 10.1186/s12936-022-04420-2

**Published:** 2023-01-03

**Authors:** Adama Gansane, Moussa Lingani, Adoke Yeka, Alain Nahum, Marielle Bouyou-Akotet, Ghyslain Mombo-Ngoma, Grace Kaguthi, Catalina Barceló, Bart Laurijssens, Cathy Cantalloube, Fiona Macintyre, Elhadj Djeriou, Andreas Jessel, Raphaël Bejuit, Helen Demarest, Anne Claire Marrast, Siaka Debe, Halidou Tinto, Afizi Kibuuka, Diolinda Nahum, Denise Patricia Mawili-Mboumba, Rella Zoleko-Manego, Irene Mugenya, Frederick Olewe, Stephan Duparc, Bernhards Ogutu

**Affiliations:** 1 Centre National de Recherche et de Formation sur le Paludisme (CNRFP), 01 BP 220801 BP 2208 Ouagadougou, Burkina Faso; 2 Institut de Recherche en Science de la Santé - Unité de Recherche Clinique de Nanoro (IRSS-URCN), Ouagadougou, Burkina Faso; 3 Infectious Diseases Research Collaboration (IDRC), Kampala, Uganda; 4 Centre de Recherches Entomologique de Cotonou (CREC), Cotonou, Benin; 5 Département de Parasitologie-Mycologie-Médecine Tropicale, Faculté de Médecine – Université des Sciences de la Santé, Libreville, Gabon; 6 Centre de Recherches Médicales de Lambaréné (CERMEL), Lambaréné, Gabon; 7 Department of Tropical Medicine, Bernhard Nocht Institute for Tropical Medicine, and University Medical Center Hamburg-Eppendorf, Hamburg, Germany; 8 Institute for Tropical Medicine, University of Tübingen, Tübingen, Germany; 9 Kenya Medical Research Institute-Centre for Respiratory Diseases Research (KEMRI-CRDR), Nairobi, Kenya; 10 Medicines for Malaria Venture, Geneva, Switzerland; 11 BEL Pharm Consulting, Chambonas, France; 12 Sanofi Research and Development, Chilly Mazarin, France; 13 Sanofi Research and Development, Bridgewater, NJ USA; 14 Centre for Clinical Research, Kenya Medical Research Institute, Kisumu, Kenya; 15 Centre for Research in Therapeutic Sciences (CREATES), Strathmore University, Nairobi, Kenya

**Keywords:** Artefenomel, Ferroquine, Combination treatment, Uncomplicated *P. falciparum* malaria, Clinical trial, Exposure–response

## Abstract

**Background:**

The contribution of artefenomel to the clinical and parasiticidal activity of ferroquine and artefenomel in combination in uncomplicated *Plasmodium falciparum* malaria was investigated.

**Methods:**

This Phase 2a, randomized, open-label, parallel-group study was conducted from 11th September 2018 to 6th November 2019 across seven centres in Benin, Burkina Faso, Gabon, Kenya, and Uganda. Patients aged ≥ 14–69 years with microscopically confirmed infection (≥ 3000 to ≤ 50,000 parasites/µL blood) were randomized 1:1:1:1 to 400 mg ferroquine, or 400 mg ferroquine plus artefenomel 300, 600, or 1000 mg, administered as a single oral dose. The primary efficacy analysis was a logistic regression evaluating the contribution of artefenomel exposure to Day 28 PCR-adjusted adequate clinical and parasitological response (ACPR). Safety was also evaluated.

**Results:**

The randomized population included 140 patients. For the primary analysis in the pharmacokinetic/pharmacodynamic efficacy population (N = 121), the contribution of artefenomel AUC_0–∞_ to Day 28 PCR-adjusted ACPR was not demonstrated when accounting for ferroquine AUC_0–d28_, baseline parasitaemia, and other model covariates: odds ratio 1.1 (95% CI 0.98, 1.2; *P* = 0.245). In the per-protocol population, Day 28 PCR-adjusted ACPR was 80.8% (21/26; 95% CI 60.6, 93.4) with ferroquine alone and 90.3% (28/31; 95% CI 74.2, 98.0), 90.9% (30/33; 95% CI 75.7, 98.1) and 87.1% (27/31; 95% CI 70.2, 96.4) with 300, 600, and 1000 mg artefenomel, respectively. Median time to parasite clearance (Kaplan–Meier) was 56.1 h with ferroquine, more rapid with artefenomel, but similar for all doses (30.0 h). There were no deaths. Adverse events (AEs) of any cause occurred in 51.4% (18/35) of patients with ferroquine 400 mg alone, and 58.3% (21/36), 66.7% (24/36), and 72.7% (24/33) with 300, 600, and 1000 mg artefenomel, respectively. All AEs were of mild-to-moderate severity, and consistent with the known profiles of the compounds. Vomiting was the most reported AE. There were no cases of QTcF prolongation ≥ 500 ms or > 60 ms from baseline.

**Conclusion:**

The contribution of artefenomel exposure to the clinical and parasitological activity of ferroquine/artefenomel could not be demonstrated in this study. Parasite clearance was faster with ferroquine/artefenomel versus ferroquine alone. All treatments were well tolerated.

*Trial registration*: ClinicalTrials.gov, NCT03660839 (7 September, 2018).

**Supplementary Information:**

The online version contains supplementary material available at 10.1186/s12936-022-04420-2.

## Background

Artemisinin-based combination therapy (ACT) is the current first-line treatment for uncomplicated *Plasmodium falciparum* malaria. However, the emergence and spread of parasite strains resistant to both the artemisinin and partner components in the Greater Mekong region has undermined clinical efficacy for several approved ACT options [1, 2]. In Africa, ACT generally remain highly efficacious for the treatment of uncomplicated *P. falciparum* malaria [3–5]. However, *P. falciparum* strains harbouring *Pfkelch13* (*k13*) mutations indicative of artemisinin resistance have been found in travellers returning from Africa [6, 7], and in at least one isolate from four African countries: Mali (F446I), Tanzania (M476I), Kenya, and Malawi (P553L) [8]. The emergence of new resistant haplotypes in Africa is also possible [8, 9], and declining clinical efficacy has been observed for ACT in the Democratic Republic of Congo [10], Angola [11], and Burkina Faso [12]. Importantly, in Rwanda, de novo emergence and selection of the R561H mutation was associated with delayed parasite clearance [13, 14]. Similarly, in Uganda the independent emergence and local spread of clinically artemisinin-resistant *P. falciparum* associated with the A675V *k13* mutation has been reported [15]. However, the reports from Rwanda and Uganda are from limited samples.

Considering the emergence of clinical resistance to ACT, new treatment options are clearly required. Ferroquine and artefenomel are novel anti-malarial drug candidates and a combination of these two drugs has been proposed as a treatment for uncomplicated malaria. New anti-malarial therapeutics should be developed as fixed-dose combinations as this is expected to improve patient adherence and reduce the risk of parasite resistance developing to either drug, particularly if they have contrasting modes of action. When developing fixed-dose anti-malarial drug combinations, it is necessary to demonstrate the contribution of each component to overall efficacy and establish the safety and tolerability profile with co-administration [16–18].

Artefenomel (also known as OZ439) is a synthetic peroxide, with a similar mechanism of action to artemisinin [19, 20]. In a study conducted in Thailand in adult patients with uncomplicated malaria, single-dose artefenomel (200, 400, 800 and 1200 mg) had a parasite clearance half-life of 1.3–8.5 h for *P. falciparum* and was well tolerated [21]. In healthy volunteers, the artefenomel terminal half-life (~ 30 h) was around 15-fold greater than for dihydroartemisnin [22], and an oral dispersion formulation was found to increase artefenomel drug exposure, reduce inter-patient variability, and mitigate the effect of food [22].

Ferroquine is a 4-aminoquinoline analog, and a strong inhibitor of hemozoin formation, with high efficacy against chloroquine-resistant and ACT-resistant *P. falciparum* [23–28]. Single-dose ferroquine (400–1600 mg) was well tolerated [29], and potent parasiticidal activity was observed in a human volunteer infection study [30]. Pharmacokinetic (PK) studies indicated a half-life of 16 days for ferroquine and 31 days for the active metabolite desmethyl-ferroquine with no relevant food effect on exposure [31]. Although development of the ferroquine/artefenomel combination has subsequently been discontinued, ferroquine remains under consideration for development with other active anti-malarial candidates.

The efficacy and safety of ferroquine/artefenomel was evaluated in the ‘Ferroquine and Artefenomel in adults and children with *Plasmodium falciparum* malaria’ (FALCI) Phase 2 study, investigating artefenomel (800 mg) plus ferroquine (400, 600, 900 or 1200 mg) in African and Asian patients aged > 6 months to < 70 years with uncomplicated falciparum malaria [32]. Efficacy with the single-dose regimen was insufficient in FALCI, particularly in patients from Vietnam. Note that most Asian patients (18/20) carried the *k13* C580Y mutation known to be associated with artemisinin resistance [32].

FALCI was designed with only one dose level of artefenomel, and so there was a risk that the study would not be able to identify the contribution of artefenomel. Consequently, this parallel investigation was designed to specifically evaluate the artefenomel contribution to the efficacy of the combination in African adolescents and adults with uncomplicated *P. falciparum* malaria.

## Methods

### Study design and objective

This Phase 2a, randomized, open-label, parallel-group study was conducted from 11th September 2018 to 6th November 2019 across seven study centres in Benin (Cotonou), Burkina Faso (Banfora and Nanoro), Gabon (Libreville and Lambaréné), Kenya (Kisumu), and Uganda (Kampala). The primary objective was to show the contribution of artefenomel to the clinical and parasitological activity of artefenomel and ferroquine in combination by analysing the exposure–response of artefenomel in patients with uncomplicated falciparum malaria. To achieve its objective, some patients would receive a sub-therapeutic dosing regimen, and this was considered in the design by using dedicated risk minimization activities, i.e., selection of a patient population at low risk of severe malaria, hospitalization for at least 48 h or longer based upon the investigator’s judgment, and administration of rescue therapy as soon as there was evidence of treatment failure or systematically on Day 29. The study protocol is provided as Additional file 1.

### Treatment

Investigational products were ferroquine 100 mg capsules (Sanofi, France) and artefenomel 300/400/600 mg granules formulation (Sanofi, France) presented in a sachet with alpha tocopherol polyethylene glycol 1000 succinate formulation and sucrose. Dose selection aimed to characterize the anti-malarial contribution of artefenomel to the combination. Artefenomel has been evaluated to doses up to 1200 mg with no safety concerns, and a wide range of doses (0–1000 mg) was selected to evaluate the exposure–response. The 400 mg ferroquine dose was selected as a sub-therapeutic dose so as not to mask the contribution of artefenomel. Details of the dose selection methods are in Additional file 2.

### Randomization

Patients were randomized centrally using interactive response technology in a ratio of 1:1:1:1 to 400 mg ferroquine alone, or 400 mg ferroquine plus artefenomel 300, 600, or 1000 mg. The administration of treatments was open label as a single oral dose on Day 0 and was directly observed. Ferroquine was administered in a fasted condition. Artefenomel was prepared as a suspension in sterile water and given approximately 15 min after ferroquine. If vomiting occurred after ferroquine, the patient was not re-dosed, artefenomel was not administered, and the patient received rescue therapy. If the artefenomel dose was vomited within 5 min of administration, the patient was re-dosed. Vomiting within 5–35 min of artefenomel administration did not prompt redosing, but any remaining drug was given. Rescue anti-malarial therapy as per local recommendations was administered to patients before Day 28 if clinically indicated, if the ferroquine dose was vomited, or at Day 29 if not given previously.

### Patients

To evaluate exposure–response, a sub-therapeutic dosing regimen was to be administered. Thus, the study population was selected to be at low risk of severe malaria. Eligible participants were aged 14 to 69 years, body weight 35–95 kg, of either sex, presenting with microscopically confirmed uncomplicated *P. falciparum* malaria (≥ 3000 to ≤ 50,000 parasites/µL blood) plus fever or a history of fever in the previous 24 h. All participants were required to use effective contraception and pregnant or lactating women were excluded. Key exclusion criteria were severe malaria [33], mixed *Plasmodium* infection, clinically important medical conditions, severe vomiting or diarrhoea, severe malnutrition [34], splenectomy, known hypersensitivity to study medications, positive test for viral hepatitis, clinically relevant laboratory abnormalities, including aspartate aminotransferase (AST) > 2 times the upper limit of normal (xULN), alanine aminotransferase (ALT) > 2xULN, or total bilirubin > 1.5xULN, or Fridericia-corrected QTc (QTcF) > 450 ms. Full inclusion and exclusion criteria are detailed in the protocol (Additional file 1).

### Procedures

Screening procedures included physical examination, medical history, 12-lead electrocardiogram (ECG), vital signs, clinical laboratory tests, viral hepatitis serology, and a pregnancy test. Patients were hospitalized for at least the first 48 h following treatment administration and longer if malaria symptoms or parasitaemia persisted. Patients were followed up on Days 3, 4, 5, 6, 7, 10, 14, 21 and 28. All patients received definitive approved anti-malarial treatment on Day 29 if they had not already received rescue therapy.

Blood samples for parasite assessments were taken at screening, every 6 h until 36 h post-dose, at hours 48, 72, 96, 120, 144, and 168 post-dose, on Days 10, 14, 21, and 28, and at any time if clinically indicated. Giemsa-stained thick and thin blood films were prepared, and parasites identified and enumerated independently by two trained microscopists using standard procedures [35]. Parasite polymerase chain reaction (PCR) genotyping to differentiate recrudescence from re-infection was done centrally by the Swiss Tropical and Public Health Institute following any positive parasite assessment after initial parasite clearance, as per published recommendations [36]. Based on the *P. falciparum* marker genes *msp1*, *msp2* and *glurp*, new infection was assumed when all the alleles in parasites from the post-treatment sample were different from those in the baseline sample, for one or more loci tested. Recrudescence was defined as at least one allele at each locus common to paired samples from baseline and at recurrence [36].

Adverse events were assessed throughout the study. Additional post-treatment safety assessments were 12-lead ECGs, vital signs, haematology, and clinical laboratory tests (Additional file 1).

Blood samples were taken pre- and post-dose for PK assessments of artefenomel (12 sample time points) and ferroquine (11 sample time points) (Additional file 1). PK samples were analysed using liquid chromatography tandem mass spectroscopy (LC-MS-MS). Artefenomel concentrations were determined at Swiss BioQuant (Basel, Switzerland) with a lower limit of quantification (LLQ) of 1 ng/mL and ferroquine samples at Covance (Salt Lake City, USA) with an LLQ of 5 ng/mL. Where anti-malarial rescue therapy was administered before Day 28, blood samples were taken for artefenomel and ferroquine PK assessments and parasite assessments.

### Endpoints

The primary efficacy endpoint was PCR-adjusted adequate clinical and parasitological response (ACPR) at Day 28, defined as the absence of parasitaemia without previous treatment failure or rescue therapy, adjusted for re-infection using PCR genotyping [35].

Secondary efficacy endpoints were Day 28 ACPR unadjusted for re-infection (crude ACPR); parasitaemia at baseline then every 6 h during the first 36 h post-dose, then at 48 h and every 24 h until Day 7; parasite clearance time; time to parasitaemia re-emergence, recrudescence, or reinfection; time elapsed below the LLQ of parasitaemia; fever clearance time; observed parasite reduction ratio at 24, 48 and 72 h post-dose (observed PRR_24_, PRR_48_, and PRR_72_); parasite clearance rate; and time to parasite reduction by 50% (PC_50_) and 99% (PC_99_) of baseline parasitaemia, time for parasitaemia to reduce by 50% (T_PC50_) and 90% (T_PC90_) independent of baseline parasitaemia, and the estimated parasite reduction ratio at 24, 48 and 72 h post-dose (estimated PRR_24_, PRR_48_, and PRR_72_) (Additional file 1).

Safety endpoints were the frequency, severity, and causality of all adverse events coded using MedDRA (version 22.0), the frequency of serious adverse events, clinically important changes in clinical laboratory data, ECGs, vital signs, or physical examination. Adverse events of special interest were pregnancy, symptomatic overdose, increase in ALT ≥ 3xULN (or ≥ 2 × the baseline value if baseline ALT was ≥ ULN), QTcF ≥ 500 ms, or QTcF prolongation > 60 ms from baseline.

Pharmacokinetic assessments were secondary endpoints, but also supported the primary analysis evaluating the exposure–response for artefenomel (Additional files 3 and 4). The following individual patient exposures for artefenomel in plasma and ferroquine and desmethyl-ferroquine in blood were estimated: maximal observed concentration (*C*_max_), concentration at Day 7 post-dose (*C*_d7_), area under the concentration–time curve from time 0 to infinity (AUC_0–∞_) for artefenomel and ferroquine, and AUC from time 0 to Day 28 (AUC_0–d28_) for ferroquine and desmethyl-ferroquine only.

### Analysis populations

The safety population included all randomized patients who received one dose or a partial dose of the investigational drugs. The PK population was a sub-set of the safety population with at least one evaluable PK blood sample for either artefenomel or ferroquine. The microbiological intention-to-treat population (mITT) included all randomized patients who received the investigational drugs, had microscopically confirmed *P. falciparum* infection at baseline, and a post-baseline parasitological assessment. The per-protocol (PP) population was a sub-set of the mITT population who were evaluable for Day 28 ACPR with no major protocol violations. The pharmacokinetic/pharmacodynamic (PK/PD) efficacy population was the primary efficacy analysis population and included patients in both the PK and mITT populations who had at least one evaluable PK sample for both artefenomel and ferroquine. Thus, patients who vomited or who did not receive a complete dose of study drug were not excluded from the PK/PD efficacy population.

### Sample size

Sample size was based on the estimated efficacy for artefenomel (0, 300, 600, and 1000 mg) plus ferroquine (400 mg) derived from clinical trial simulations assuming a parasitaemia > 3000 parasites/µL (Additional file 2). Based on an estimated PCR-adjusted ACPR of 72% for ferroquine alone and 81%, 91% and 97% in the three escalating ferroquine plus artefenomel arms, 30 patients per arm would be required to detect an exposure–response effect with artefenomel with ~ 90% power. Allowing for subject withdrawals, target sample size was 140 patients (35 per arm).

### Statistical methods

For the primary efficacy analysis, data processing, PK parameter estimation and logistic regression analyses were conducted within R (3.5.1) combined with MONOLIX (MLX2019R1) and the IQR package (v1.1.1) developed by IntiQuan (Basel, Switzerland) to support the entire workflow of a PK and logistic regression analysis from estimations to simulations. For simulations, IQR uses the library SUNDIALS (v2.9.0) from Computation (USA) (Additional files 3 and 4).

The contribution of artefenomel exposure to the Day 28 PCR-adjusted ACPR of the combination was evaluated using logistic regression evaluating the exposure to artefenomel (AUC_0–∞_) and ferroquine/desmethyl-ferroquine (AUC_0–d28_) as covariates as well as baseline parasitaemia, age, body weight, sex, vomit status, and study centre (Additional file 4). Data exploration suggested that three study centres (Libreville, Lambaréné, and Cotonou) had lower efficacy (Day 28 PCR-adjusted ACPR ≤ 75%) compared to the other centres (≥ 85%). These sites were identified based on their efficacy data and no quality issues were identified in the data review of this study. The covariate ‘low efficacy study centre’ was created by grouping these three centres versus all other centres to identify any study centre effects.

The base model included ferroquine AUC_0–d28_ as the predictor variable and the contribution of each of the remaining potential covariates on the base model was first assessed in a univariate addition analysis. A backward elimination approach was then implemented including all significant covariates from the univariate addition analysis, plus artefenomel exposure. Model selection was based on Akaike Information Criterion. Odds ratio estimates, corresponding 95% two-sided Wald confidence intervals (CI) and *P* values were calculated for covariates. As a secondary efficacy analysis, the relationship between the estimated exposure of artefenomel and ferroquine and Day 28 crude ACPR was evaluated as described for Day 28 PCR-adjusted ACPR.

For other secondary efficacy outcomes and safety outcomes, statistical analysis was done using SAS (version 9.4). Day 28 PCR-adjusted and crude ACPR were summarized for the PP and mITT populations, with Clopper–Pearson 95% CI. All other secondary efficacy outcomes were evaluated in the mITT population. Parasite clearance parameters were calculated using the WorldWide Antimalarial Resistance Network parasite clearance estimator (WWARN PCE) based on the linear portion of the individual natural logarithm parasitaemia–time profiles [37]. The time to each parasite clearance endpoint, parasite re-emergence, recrudescence, re-infection, fever clearance, and elapsed time below the LLQ of parasitaemia were estimated using Kaplan–Meier analysis. There was no adjustment for multiplicity of comparisons in this exploratory study.

Pharmacokinetic analysis was performed using non-linear mixed effect modelling as implemented in Monolix (version 2019R1), applying previously developed population PK models (see Additional file 3).

### Ethics

The study was conducted in accordance with the Declaration of Helsinki, Good Clinical Practice, and all applicable laws, rules, and regulations of the participating countries. Final approval by the relevant Independent Ethics Committees and, where relevant, local regulatory authorities, was obtained at each participating study centre before any patient was enrolled. All patients or their legal guardians provided informed consent and those under the age of legal majority provided assent.

## Results

### Patients

**Fig. 1:**
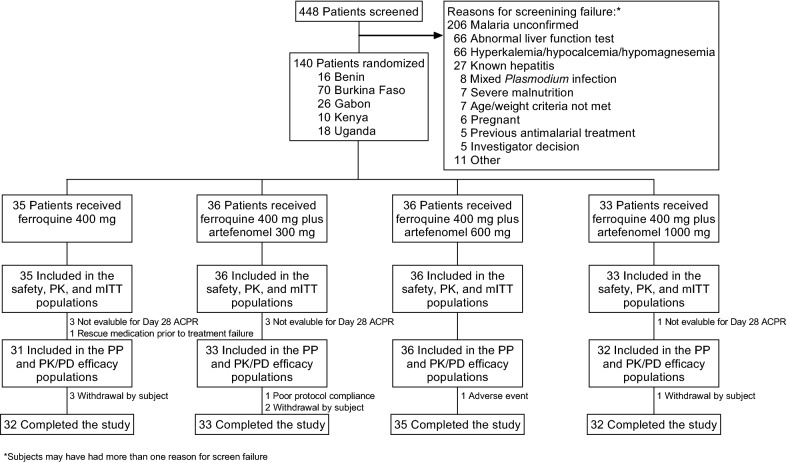
Participant flow. PK, pharmacokinetic; PK/PD, pharmacokinetic/pharmacodynamic; PP, per-protocol; mITT, microbiological intention-to-treat

The randomized population comprised 140 patients, all of whom were included in the safety, mITT, and PK populations (Fig. 1). Eight patients discontinued the study prematurely and were excluded from the PP and PK/PD efficacy populations, seven because of missing data on Day 28 and one because rescue therapy was administered before treatment failure (Fig. 1).

Demographic and baseline characteristics were similar across all the treatment arms (Table 1). Patients’ mean age was 22.5 (standard deviation [SD] 11.3) years, mean weight 56.4 (SD 11.2) kg and females comprised 69.3% (97/140) of the population. Mean baseline parasitaemia was 17,953 (SD 13,092) parasites/µL blood.

**Table 1:** Baseline demographic and clinical characteristics (randomized population)

Characteristic	Ferroquine 400 mg (N = 35)	Ferroquine 400 mg plus artefenomel		
		300 mg (N = 36)	600 mg (N = 36)	1000 mg (N = 33)
Age, years	25.4 (13.2)	21.8 (11.5)	20.7 (8.6)	22.2 (11.3)
Female, n (%)	23 (65.7)	28 (77.8)	26 (72.2)	20 (60.6)
Weight, kg	57.8 (11.1)	54.8 (10.0)	54.9 (11.6)	58.4 (12.1)
BMI, kg/m^2^	21.1 (3.3)	20.5 (3.6)	20.6 (3.3)	21.5 (4.0)
Fever present, n (%)	12 (34.3)	16 (44.4)	16 (44.4)	16 (48.5)
Parasitaemia, parasites/µL	17,496 (11,890) [3188 to 46,076]	18,800 (13,559) [3017 to 45,061]	15,172 (13,013) [2848 to 54,141]	20,547 (13,835) [3517 to 51,862]
Parasitaemia, n (%)				
< 3000, parasites/µL	0	0	1 (2.8)	0
3000–10,000, parasites/µL	11 (31.4)	14 (38.9)	15 (41.7)	11 (33.3)
10,000–50,000, parasites/µL	24 (68.6)	22 (61.1)	19 (52.8)	21 (63.6)
> 50,000, parasites/µL	0	0	1 (2.8)	1 (3.0)

Values are mean (standard deviation) [range] unless otherwise indicated

All participants were self-declared of black race

*BMI* body mass index

One patient in the ferroquine monotherapy group vomited within 5 min of ferroquine administration, but completed the study. In the ferroquine plus artefenomel groups, vomiting occurred between 5 and 35 min after administration in 0% (0/36), 5.6% (2/36), and 3.0% (1/33) of patients in the 300 mg, 600 mg, and 1000 mg arms, respectively. After 35 min following dosing, vomiting was noted for 8.3% (3/36) of patients with 300 mg, 11.1% (4/36) with 600 mg, 24.2% (8/33) with 1000 mg artefenomel. No patients were given rescue medication because of vomiting.

### Primary analysis: efficacy exposure–response

#### PK/PD efficacy population

The PK/PD efficacy population included 132 patients, but five cases of reinfection and six with undetermined PCR results were considered missing for the Day 28 PCR-adjusted ACPR analysis, with 121 patients evaluable. Baseline demographic and clinical characteristics, drug exposure, and Day 28 PCR-adjusted ACPR outcome for this population are shown in Additional file 5: Table S1.

#### Exposure

**Fig. 2:**
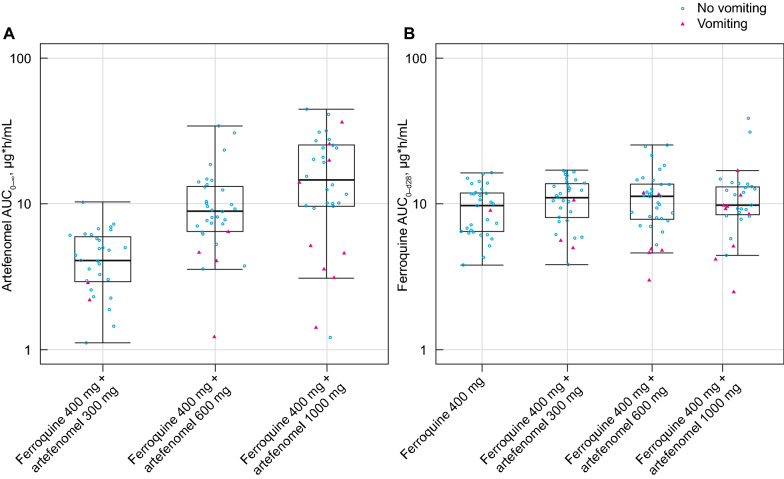
Artefenomel AUC_0–∞_ and ferroquine AUC_0–d28_ distribution across treatment groups. PK/PD efficacy population

Details of the PK analysis supporting the primary endpoint including concentration–time profiles and summaries of estimated exposures for artefenomel, ferroquine and desmethyl-ferroquine relative to dosing are provided in Additional file 3. Observations were well described by the historical population PK models, and the estimated exposures were within the expected range for the patients in this study. The distributions of artefenomel AUC_0–∞_ and ferroquine AUC_0–d28_ are shown in Fig. 2.

#### Response

**Fig. 3:**
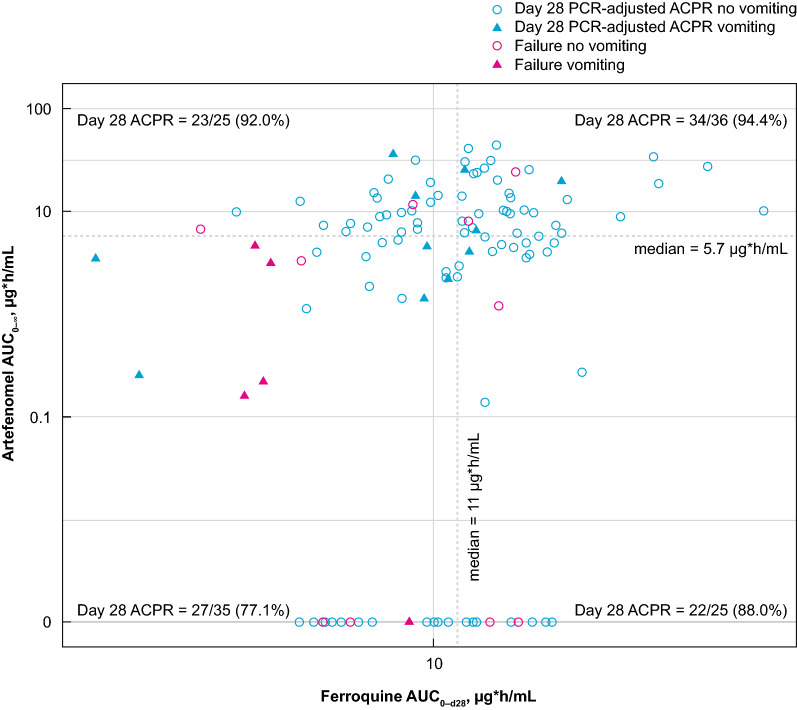
Graphical representation of Day 28 PCR-adjusted ACPR compared with ferroquine and artefenomel exposure. PK/PD efficacy population. Dotted lines represent median exposure in the PK/PD efficacy population for each drug

Day 28 PCR-adjusted ACPR was achieved by 94.4% (34/36) of patients who had an estimated AUC for both artefenomel and ferroquine above the median value, 92.0% (23/25) of patients when only exposure to artefenomel was above its median value, 88.0% (22/25) when only ferroquine exposure was above its median value, and 77.1% (27/35) when exposure to both drugs was below the median value (Fig. 3).

#### Exposure–response analysis

Full details of the exposure–response analysis are provided in Additional file 4. Ferroquine AUC_0–d28_, baseline parasitaemia, and low efficacy study centre were statistically significant covariates for Day 28 PCR-adjusted ACPR in the univariate analysis (*P* < 0.05), whereas artefenomel exposure was not. Univariate analysis indicated no effect on Day 28 PCR-adjusted ACPR of desmethyl-ferroquine exposure (*P* = 0.113), age (*P* = 0.192), sex (*P* = 0.0524), or body weight (*P* = 0.158). As exploring the contribution of artefenomel exposure to Day 28 PCR-adjusted ACPR was the primary objective, this covariate was included for the backward elimination analysis. The backward elimination suggested only the removal of artefenomel exposure.

**Fig. 4:**
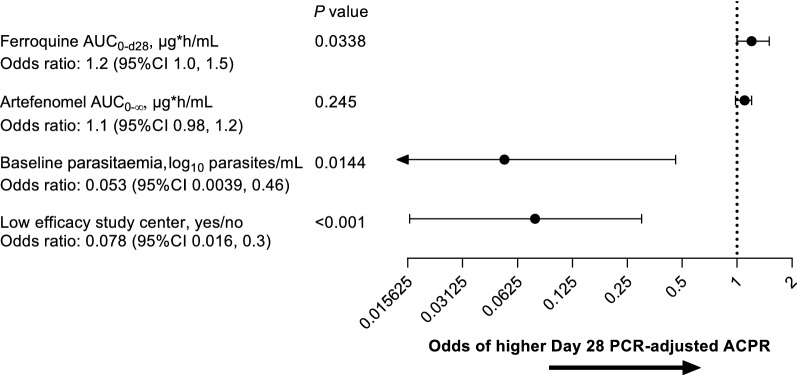
Logistic regression model for the exposure–response analysis for Day 28 PCR-adjusted ACPR. PK/PD efficacy population

In the model including artefenomel exposure, the odds ratio of artefenomel AUC_0–∞_ for Day 28 PCR-adjusted ACPR was 1.1 (95% CI 0.98, 1.2; *P* = 0.245) (Fig. 4). Therefore, the contribution of artefenomel exposure to the antimalarial efficacy of ferroquine and artefenomel in combination was not demonstrated.

The final logistic regression model for the efficacy exposure–response analysis indicated that Day 28 PCR-adjusted ACPR could be described as a function of ferroquine AUC_0–d28_, baseline parasitaemia, and low efficacy study centre (Additional file 5: Fig. S1).

Repeating the primary analysis for Day 28 crude ACPR showed similar results, with no statistically significant contribution of artefenomel AUC_0–∞_ to the Day 28 crude ACPR (odds ratio 1.0 [95% CI 0.98, 1.1] *P* = 0.21) (Additional file 5: Fig. S2).

### Secondary efficacy endpoints

#### Day 28 ACPR

**Fig. 5:**
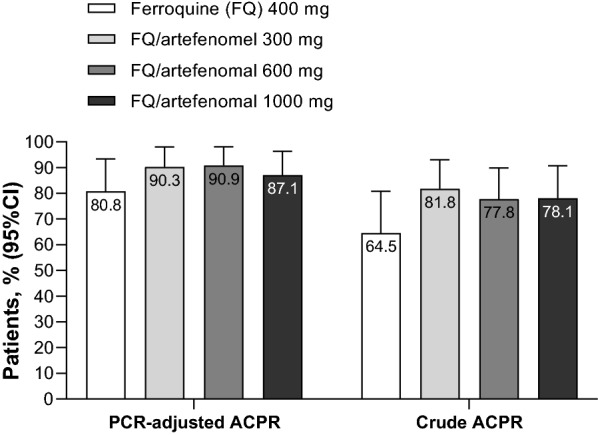
Day 28 PCR-adjusted ACPR and crude ACPR. PP population

In the PP population, Day 28 PCR-adjusted ACPR was 80.8% (21/26; 95% CI 60.6, 93.4) with ferroquine monotherapy. There was a trend for higher efficacy with artefenomel co-administration, though efficacy was similar across the artefenomel doses: 90.3% (28/31; 95% CI 74.2, 98.0) with 300 mg, 90.9% (30/33; 95% CI 75.7, 98.1) with 600 mg, and 87.1% (27/31; 95% CI 70.2, 96.4) with 1000 mg (Fig. 5).

Based on PCR-genotyping, there were 2/31 (6.5%) patients with reinfection in the ferroquine group, and 1/33 (3.0%), 2/36 (5.6%) and 0/32 (0%) with artefenomel 300, 600 and 1000 mg, respectively. Six of the recurrences had undetermined PCR results.

Day 28 crude ACPR was 64.5% (20/31; 95% CI 45.4, 80.8) for ferroquine alone, with a trend for higher efficacy in the artefenomel arms: 81.8% (27/33; 95% CI 64.5, 93.0) with 300 mg, 77.8% (28/36; 95% CI 60.8, 89.9) with 600 mg, and 78.1% (25/32; 95% CI 60.0, 90.7) with 1000 mg (Fig. 5). Similar trends were observed for the mITT population (Additional file 5: Table S2).

#### Parasite re-emergence, re-infection, and recrudescence

**Fig. 6:**
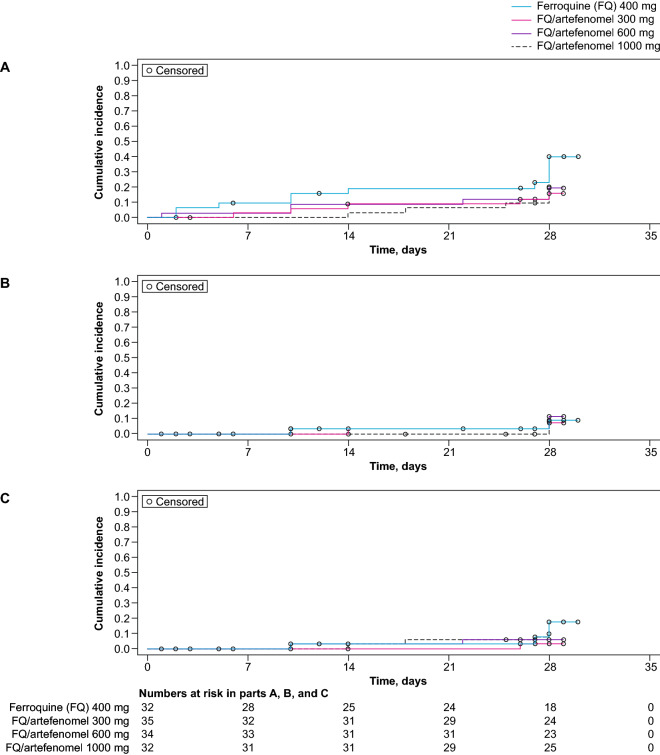
Time to parasite re-emergence, reinfection, or recrudescence. mITT population. A) Time to parasite re-emergence; B) Time to reinfection; C) Time to recrudescence

The median time to parasite re-emergence, re-infection, and recrudescence could not be calculated (Kaplan–Meier). However, there was a trend over the 28-day study period for a lower probability of re-emergence and recrudescence with the combination arms versus ferroquine alone (Fig. 6).

#### Parasite and fever clearance

**Fig. 7:**
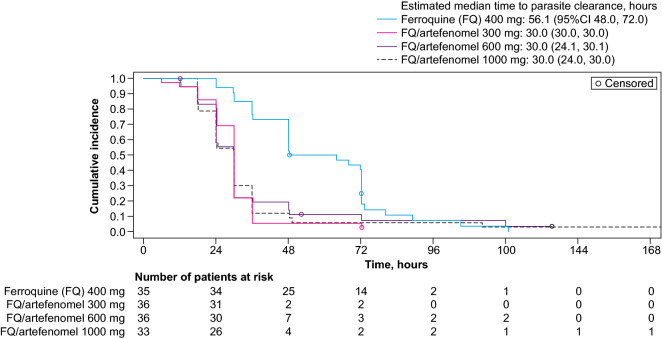
Parasite clearance time. mITT population

The estimated median time to parasite clearance (Kaplan–Meier) was 56.1 h (95% CI 48.0, 72.0) with ferroquine alone, but was more rapid in the combination arms, without an effect of artefenomel dose: 30.0 h (95% CI 30.0, 30.0) with 300 mg, 30.0 h (95% CI 24.1, 30.1) with 600 mg, and 30.0 h (95% CI 24.0, 30.0) with 1000 mg artefenomel (Fig. 7). The estimated time below the LLQ of parasitaemia was similar in the ferroquine group (25.0 days [95% CI 23.0, 26.0]) and the combination arms, 26.0 days (95% CI 26.0, 27.0) with 300 mg, 26.5 days (95% CI 26.0, 27.0) with 600 mg, and 26.0 days (95% CI 26.0, 27.0) with 1000 mg artefenomel (Additional file 5: Fig. S3). Median time to fever clearance was ~ 1 h across all the treatment groups (Additional file 5: Fig. S4).

Parasite clearance kinetics showed a trend for improvement in the ferroquine/artefenomel combination arms versus ferroquine alone, with no apparent trend between the different artefenomel dosing arms (Table 2; Additional file 5: Table S3). For example, the mean estimated PRR_48_ (log_10_) was 4.08 (SD 1.59) with ferroquine alone and ranged between 7.46 and 8.26 in the combination arms. Similarly, mean PC_50_ was 14.07 (SD5.6) compared with 7.74 to 9.47 for the combination arms (Table 2).

**Table 2:** Parasite clearance kinetics (mITT population)

Endpoint	Ferroquine 400 mg (N = 35)	Ferroquine 400 mg plus artefenomel		
		300 mg (N = 36)	600 mg (N = 36)	1000 mg (N = 33)
Parasite clearance rate (1/h)	n = 21	n = 28	n = 28	n = 25
Mean (SD) [range]	0.20 (0.08) [0.10 to 0.39]	0.36 (0.10) [0.17 to 0.67]	0.40 (0.12) [0.22 to 0.79]	0.36 (0.12) [0.07 to 0.60]
Estimated PRR_24_, log_10_	n = 21	n = 28	n = 28	n = 25
Mean (SD) [range]	2.0 (0.79) [1.00 to 4.09]	3.76 (1.04) [1.74 to 6.97]	4.13 (1.25) [2.29 to 8.23]	3.73 (1.30) [0.70 to 6.30]
Estimated PRR_48_, log_10_	n = 21	n = 28	n = 28	n = 25
Mean (SD) [range]	4.08 (1.59) [1.99 to 8.18]	7.52 (2.08) [3.48 to 13.94]	8.26 (2.50) [4.57 to 16.46]	7.46 (2.60) [1.41 to 12.60]
Estimated PRR_72_, log_10_	n = 21	n = 28	n = 28	n = 25
Mean (SD) [range]	6.12 (2.38) [2.99 to 12.27]	11.28 (3.12) [5.22 to 20.90]	12.39 (3.75) [6.86 to 24.69]	11.20 (3.90) [2.11 to 18.90]
PC_50_, h	n = 21	n = 28	n = 28	n = 25
Mean (SD) [range]	14.07 (5.60) [6.9 to 25.3]	7.74 (4.64) [0.4 to 19.9]	7.78 (3.79) [0.5 to 15.0]	9.47 (7.98) [0.4 to 33.3]
T_PC50_, h	n = 21	n = 28	n = 28	n = 25
Geometric mean point estimate (95% CI)	3.79 (3.29, 4.38)	1.99 (1.75, 2.25)	1.81 (1.60, 2.05)	2.09 (1.84, 2.39)
PC_99_, h	n = 21	n = 28	n = 28	n = 25
Mean (SD) [range]	36.92 (9.99) [24.6 to 56.0]	19.33 (6.11) [6.6 to 33.1]	18.34 (5.52) [5.6 to 29.4]	22.95 (17.07) [6.9 to 90.1]
T_PC99_, h	n = 21	n = 28	n = 28	n = 25
Geometric mean point estimate (95% CI)	25.21 (21.84, 29.10)	13.20 (11.66, 14.94)	12.05 (10.64, 13.65)	13.91 (12.20, 15.87)

*PRR* parasite reduction ratio

PC_50_ and PC_99_ were the time taken for parasitaemia to reduce by 50% and 99% from baseline parasitaemia, respectively, based on the linear model fitted to the linear part of the profile; T_PC50_ and T_PC99_ were the time taken for parasitaemia to reduce by 50% and 99%, respectively, based upon the parasite clearance rate, independent of the initial parasitaemia. PC_50_ PC_99_ and the parasite clearance rate were estimated using the WWARN PCE based upon the linear part of the individual natural log parasitaemia–time profiles. Data from patients with a poor fit to the linear model (r^2^ < 0.75) were excluded from the analysis

### Pharmacokinetic parameters

In addition to supporting the primary efficacy analysis, PK parameters were reported for the PK population (Additional file 5: Table S4). Geometric mean ferroquine and desmethyl-ferroquine exposures were similar across the treatment groups with a between-patient variability that was moderate to high. Ferroquine and desmethyl-ferroquine exposures were highly correlated. Artefenomel exposures were approximately dose proportional, with substantial inter-patient variability.

### Safety and tolerability

**Fig. 8:**
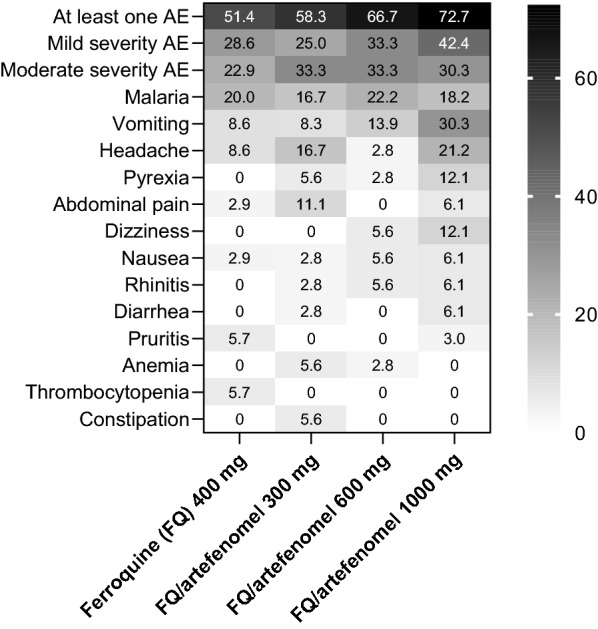
Most common treatment emergent adverse events of any cause. Safety population. Data are % patients for adverse events occurring in > 3% of patients in any treatment group. AE, adverse event

Treatment emergent adverse events of any cause were reported in 51.4% (18/35) of patients in the ferroquine only group, and in the combination arms for 58.3% (21/36) with 300 mg, 66.7% (24/36) with 600 mg, and 72.7% (24/33) with 1000 mg artefenomel. The most common adverse events were malaria (20.0%; 7/35) with ferroquine alone, malaria and headache (both 16.7%; 6/36) with artefenomel 300 mg, malaria (22.2%; 8/36) with artefenomel 600 mg, and vomiting (30.3%; 10/33) with 1000 mg artefenomel (Fig. 8, Additional file 5: Table S5). There was a trend for higher rates of vomiting and dizziness with increasing artefenomel dose (Fig. 8).

All adverse events were of mild-to-moderate severity (Fig. 8). There were no deaths, severe adverse events, or adverse events leading to treatment discontinuation in any group. There was one pregnancy in the ferroquine/artefenomel 300 mg arm, with unknown outcome. There was one serious adverse event in the ferroquine plus artefenomel 600 mg group (1/36; 2.8%) of severe malaria of moderate severity which was not considered related to treatment. This 16-year-old female had a baseline parasitaemia of 11,888 parasites/µL blood and vomited 38 min after treatment. At follow-up on Day 3, the participant remained febrile (39.0 °C) with a parasite count of 18,596 /µL blood. Artemether-lumefantrine was administered, but the patient vomited. As their body temperature had increased to 41.1 °C, blood pressure was 101/51 mmHg, and there were signs of general weakness a diagnosis of complicated malaria was made and treatment with artemether (intramuscular injection) initiated with rapid resolution of symptoms and parasite clearance confirmed on Day 5.

Adverse events considered in the investigator’s opinion related to drug administration occurred in 7.9% (11/140) of patients with ferroquine, most commonly vomiting (3.6%; 5/140) (Additional file 5: Table S6), and with artefenomel in 8.3% (3/36) of patients with 300 mg, 13.9% (5/36) with 600 mg, and 27.3% (9/33) with 1000 mg. Vomiting was the most common artefenomel-related event, reported for 5.6% (2/36) of patients with 300 mg, 8.3% (3/36) with 600 mg, and 24.2% (8/33) with 1000 mg (Additional file 5: Table S7).

There was one case of ALT increased in the ferroquine/artefenomel 1000 mg arm (3.0%; 1/33) observed in a female patient with baseline elevations of ALT (1.1xULN), alkaline phosphatase (ALP) (3.2xULN), and total bilirubin (1.2xULN), and no history of hepato-biliary disorders. From Day 5, ALT increased to a maximum of 3.3xULN on Day 15, with concurrent ALP 5.4xULN, AST 1.3xULN, and total bilirubin 1.2xULN. Total bilirubin continued to rise, peaking at 3.4xULN on Day 25 with direct bilirubin at 4.7xULN and ALP at 5xULN. There were no symptoms, and ALT, AST, and total bilirubin values had returned to normal by Day 42 without intervention. Further elevations in ALT (1.7xULN), AST (1.5xULN), total bilirubin (1.3xULN), and ALP (2.6xULN) were noted on Day 96, but all measures had resolved to baseline levels by Day 141.

There was no difference in the maximum post-baseline change in haematology parameters between treatment groups, which were consistent with recovery from malaria (Additional file 5: Table S8). There were no trends by artefenomel dose in the frequency of potentially clinically relevant changes in haematology parameters (Additional file 5: Fig. S5). Changes in clinical laboratory parameters showed no trends according to artefenomel dose (Additional file 5: Table S8). Excepting the case of increased ALT discussed above, there were no other clinically important ALT findings (Additional file 5: Fig. S6).

There were no differences between treatment arms in vital signs (Additional file 5: Table S9). A decrease in heart rate was observed for all groups, consistent with recovery from malaria. There were no cases of QTcF ≥ 500 ms, or QTcF prolongation > 60 ms from baseline, or when using Bazett’s correction (QTcB) (Additional file 5: Table S10).

## Discussion

The contribution of artefenomel exposure (AUC_0–∞_) to the clinical and parasiticidal activity of the artefenomel/ferroquine combination, defined as Day 28 PCR-adjusted ACPR, could not be identified in this study. The study also failed to identify the contribution of artefenomel exposure to the effect of the artefenomel/ferroquine combination for Day 28 crude ACPR. This occurred even though clinical trial simulations were performed to support the dose selection and sample size, and even though a wide range of individual artefenomel exposures (0–13.05 µg*h/ml) were observed.

Graphical explorations of the relationship between exposure and Day 28 PCR-adjusted ACPR were suggestive of a contribution of artefenomel to the efficacy of the artefenomel/ferroquine combination. In addition, parasite clearance parameters consistently demonstrated more rapid clearance with the artefenomel/ferroquine combination compared with ferroquine alone, indicating a contribution of both drugs to early parasite clearance. Importantly, for the considerably larger FALCI study, which ran in parallel, the exposure-dependent contribution of both artefenomel and ferroquine to both Day 28 PCR-adjusted ACPR and crude ACPR could be estimated, even if the overall efficacy of the exploratory dose regimens in FALCI was sub-optimal [32].

It seems, therefore, likely that the current study was not adequately powered to identify a statistically significant contribution rather than a lack of a contribution. A possible explanation for this is the higher-than-expected response with 400 mg ferroquine alone in the current study. The clinical trial simulations used to inform the study design were based on a predicted ferroquine Day 28 PCR-adjusted ACPR of 72% (90% CI 57.0, 84.0), whereas the observed response with ferroquine alone (80.8%) was close to the upper limit of predicted efficacy. With a lower than predicted number of treatment failures across all treatment arms, it was statistically more difficult to detect a significant contribution of artefenomel.

The reasons for a higher-than-expected response to ferroquine are unclear. However, this does not appear to result from higher than anticipated ferroquine or active metabolite exposures in this study population. Notably, limited exposure–response data were available for ferroquine to inform the clinical trial simulations and no efficacy data for the artefenomel/ferroquine combination were available. Consequently, there was considerable uncertainty associated with the predictions for ferroquine exposure–response. However, this uncertainty in the predicted ferroquine response was not considered in the clinical trial simulations.

The upper limit for parasitaemia for enrolment in this study was 50,000 parasites/µL for ethical reasons, considerably lower than the maximum parasitaemia level used in the clinical trial simulations (316,228 parasites/µL). Both in this study and in FALCI, higher baseline parasitaemia was shown to significantly decrease the odds of Day 28 PCR-adjusted ACPR [32]. The low baseline parasitaemia in the current study population is, therefore, likely to have increased the observed response in this study compared with the simulations.

In FALCI, there was evidence that age influenced baseline parasitaemia [32]. African patients aged ≤ 5 years had a median baseline parasitaemia that was approximately fivefold higher than observed for older patients (31,219 versus 5962 parasites/µL), likely related to increased immunity in older patients. FALCI also indicated that on average, higher drug exposures were required to achieve efficacy in African children ≤ 5 years versus older patients. In the current study and for ethical considerations, only African patients over 14 years of age were recruited, with a median baseline parasitaemia in the PK/PD efficacy population of 14,570 parasites/µL. The observations from FALCI suggest that even if the study inclusion criteria had allowed a higher upper limit for baseline parasitaemia, studying adult patients would still result in low baseline parasitaemia and a lower dose of ferroquine would have been required to establish the exposure–response.

Overall, eligible participants were aged 14 to 69 years, with *P. falciparum* parasitaemia of ≥ 3,000 to ≤ 50,000 parasites/µL blood. Given that the ferroquine only arm was not expected to achieve clinical cure rates > 72%, this population was selected to minimize the risk of developing severe malaria. Note that data from Burkina Faso indicate that asymptomatic carriage of malaria is rare at parasite densities ≥ 2,500 parasites/µL blood, occurring at a rate of 5.3% (7/133) in patients aged > 15 years [38]. Similarly, in Kenya geometric mean parasite density determined by microscopy in asymptomatic individuals has been reported as 1014 parasite/μL (95% CI: 940–1094) [39]. Thus, as the patient population in the current study had parasite densities ≥ 3,000 parasites/µL blood plus fever or history of fever, it is most likely that their fever was caused by malaria. However, it is recognized that in the relatively high transmission settings for this study, the eligibility criteria may have resulted in a patient population that could have sufficient partial immunity to drive down parasitaemia and support the higher-than-expected cure rate for ferroquine monotherapy and the other treatment regimens.

No new safety signals were identified, and the safety findings were consistent with previous clinical studies [29, 32, 40–43]. The combination of ferroquine and artefenomel was well tolerated at all doses, with only mild-to-moderate adverse events. The frequency of mild adverse events increased with increasing artefenomel dose, particularly vomiting and dizziness, but moderate adverse events were of similar frequency across the artefenomel dosing arms. Vomiting within 6 h of initial artefenomel administration was also observed in FALCI in 24.6% (90/366) of patients and was not associated with ferroquine dose [32].

Although there was one case of increased ALT, there was no suggestion of a relationship between artefenomel dosing and ALT increases. No patient had a QTcB or QTcF > 500 ms or an increase from baseline > 60 ms. These findings contrast with FALCI, as increased ALT was reported in 2.1% (8/373) of patients and appeared to be associated with ferroquine [32]. There was also evidence of a dose effect with ferroquine on QTcF and QTcB for increases from baseline > 60 ms, as well as three confirmed cases of QTcB > 500 ms [32].

This study illustrates the challenges in designing clinical trials to demonstrate the contribution of individual drugs to the overall efficacy of a combination therapy in a disease which if not rapidly treated can progress to a serious and life-threatening condition. To explore the contribution of the individual drugs, sub-therapeutic doses must be administered. However, the risk to the patients from disease progression must also be minimized. Consequently, in this study the patient population excluded children < 14 years of age who are most at risk of severe malaria, restricted baseline parasitaemia to the range ≥ 3000 to ≤ 50,000 parasites/µL blood and used drug doses that were not substantially sub-therapeutic.

## Conclusion

The contribution of artefenomel exposure to Day 28 PCR-adjusted ACPR could not be demonstrated. The main reason was likely the higher than anticipated efficacy with ferroquine alone which reduced the power of the study to identify the contribution of artefenomel to the drug combination, rather than a true lack of contribution. More rapid parasite clearance was demonstrated with the artefenomel/ferroquine combination compared to ferroquine alone, clearly showing the contribution of both drugs to parasite clearance. The combination was generally well tolerated, and the safety profile was consistent with the known profiles of the two compounds.

## Supplementary Information

**Additional file 1: **Study protocol.

**Additional file 2**: Clinical trial simulation to determine the dose and sample sizes.

**Additional file 3**: Pharmacokinetic analysis supporting the primary analysis.

**Additional file 4**: Exposure–response pharmacokinetic/pharmacodynamic analysis.

**Additional file 5**. Supplementary tables and figures.

## Data Availability

The data sets used and/or analysed during the current study are available from the corresponding author on reasonable request.

## Abbreviations

ACPR: Adequate clinical and parasitological response
ACT: Artemisinin-based combination therapy
ALP: Alkaline phosphatase
ALT: Alanine aminotransferase
AST: Aspartate aminotransferase
AUC_0–d28_: Area under the curve from time 0 to Day 28
AUC_0–∞_: Area under the curve from time 0 to infinity
*C*_d7_: Concentration at Day 7 post-dose
CI: Confidence interval
*C*_max_: Maximal observed concentration
ECG: Electrocardiogram
FALCI: ‘Ferroquine and artefenomel in adults and children with *Plasmodium falciparum* malaria’ study
LC-MS/MS: Liquid chromatography tandem mass spectroscopy
LLQ: Lower limit of quantitation
MedDRA: Medical Dictionary for Regulatory Activities
mITT: Microbiological intention-to-treat
PC_50_ and PC_99_: Time taken for parasitaemia to reduce by 50% and 99% of baseline parasitaemia, respectively.
PCR: Polymerase chain reaction
PD: Pharmacodynamic
PK: Pharmacokinetic
PP: Per-protocol
PRR_24_: Parasite reduction ratio at 24 h
PRR_48_: Parasite reduction ratio at 48 h
PRR_72_: Parasite reduction ratio at 72 h
QTc: Corrected QT interval
QTcB: Corrected QT interval using Bazett’s formula
QTcF: Corrected QT interval using Fridericia’s formula
SD: Standard deviation
T_PC50_ and T_PC90_: Time to parasite reduction by 50% and 90%, respectively, independent of baseline parasitaemia
ULN: Upper limit of normal
WWARN PCE: WorldWide Antimalarial Resistance Network parasite clearance estimator
