# Supplementary material for: Randomized, open-label, phase 2a study to evaluate the contribution of artefenomel to the clinical and parasiticidal activity of artefenomel plus ferroquine in African patients with uncomplicated Plasmodium falciparum malaria
Source: Malar J. 2023 Jan 3;22:2. doi: 10.1186/s12936-022-04420-2 (PMC9809015; doi:10.1186/s12936-022-04420-2)
Supplement: Supplementary file 1 — Additional file 1: Study protocol. [file 12936_2022_4420_MOESM1_ESM.pdf]

CLINICAL TRIAL PROTOCOL

**Protocol title:**	**A Randomized, Open label, Parallel-group, Single Dose Regimen, Phase 2a Study, to Investigate the Clinical and Parasiticidal Activity and the Pharmacokinetics of 3 dose levels of Artefenomel (OZ439) given in combination with Ferroquine (FQ) and FQ alone, in African Patients with Uncomplicated *Plasmodium falciparum* Malaria**
**Protocol number:**	**ACT14655**
**Amendment number:**	**Not applicable**
**Compound number (Trademark/INN):**	**OZ439 (artefenomel) and SSR97193 (ferroquine)**
**Short title:**	**A Randomized, Open, Parallel-group, Single Dose, Phase 2a Study, to Investigate the Clinical and Parasiticidal Activity and the Pharmacokinetics of 3 dose levels of Artefenomel in combination with Ferroquine (FQ) and FQ alone, in African Patients with Uncomplicated *Plasmodium falciparum* Malaria**
**Sponsor name and legal registered address:**	

Regulatory agency identifying numbers:

IND number:	FQ: IND115244
	OZ439: IND104549
UTN number:	U1111-1191-5573

Approval Date: 13-Nov-2017 Total number of pages: 93

Any and all information presented in this document shall be treated as confidential and shall remain the exclusive property of Sanofi (or any of its affiliated companies). The use of such confidential information must be restricted to the recipient for the agreed purpose and must not be disclosed, published or otherwise communicated to any unauthorized persons, for any reason, in any form whatsoever without the prior written consent of Sanofi (or the concerned affiliated company); 'affiliated company' means any corporation, partnership or other entity which at the date of communication or afterwards (i) controls directly or indirectly Sanofi, (ii) is directly or indirectly controlled by Sanofi, with 'control' meaning direct or indirect ownership of more than 50% of the capital stock or the voting rights in such corporation, partnership or other entity

Sponsor Signatory:

[redacted]

[redacted]

Monitoring Team's Representative Name and Contact Information:

TABLE OF CONTENTS

CLINICAL TRIAL PROTOCOL 1

TABLE OF CONTENTS 3

LIST OF TABLES 6

LIST OF FIGURES 6

1 SYNOPSIS 7

2 SCHEDULE OF ACTIVITIES (SOA) 11

3 INTRODUCTION 15

3.1 STUDY RATIONALE 15

3.2 BACKGROUND 16

3.3 BENEFIT/RISK ASSESSMENT 16

4 OBJECTIVES AND ENDPOINTS 18

4.1 APPROPRIATENESS OF MEASUREMENTS 19

5 STUDY DESIGN 21

5.1 OVERALL DESIGN 21

5.2 PARTICIPANT AND STUDY COMPLETION 22

5.3 END OF STUDY DEFINITION 22

5.4 SCIENTIFIC RATIONALE FOR STUDY DESIGN 23

5.5 JUSTIFICATION FOR DOSE 23

6 STUDY POPULATION 25

6.1 INCLUSION CRITERIA 25

6.2 EXCLUSION CRITERIA 26

6.3 LIFESTYLE RESTRICTIONS 28
6.3.1 Meals and dietary restrictions 28

6.4 SCREEN FAILURES 28

7 TREATMENTS 29

7.1 TREATMENTS ADMINISTERED 29
7.1.1 Investigational Medicinal Products....29
7.1.2 Noninvestigational Medicinal Products 30
7.2 DOSE MODIFICATION....30
7.3 METHOD OF TREATMENT ASSIGNMENT 30
7.4 BLINDING 31
7.5 PREPARATION/HANDLING/STORAGE/ACCOUNTABILITY....31
7.6 TREATMENT COMPLIANCE 32
7.7 CONCOMITANT THERAPY 32
7.7.1 Rescue medicine....33
7.8 TREATMENT AFTER THE END OF THE STUDY....34
8 DISCONTINUATION/WITHDRAWAL CRITERIA 35
8.1 DISCONTINUATION OF STUDY TREATMENT 35
8.2 WITHDRAWAL FROM THE STUDY 35
8.3 LOST TO FOLLOW UP 35
9 STUDY ASSESSMENTS AND PROCEDURES 37
9.1 EFFICACY ASSESSMENTS 37
9.1.1 MEASUREMENT OF PARASITEMIA....37
9.2 ADVERSE EVENTS....39
9.2.1 Time period and frequency for collecting AE and SAE information....41
9.2.2 Method of detecting AEs and SAEs....41
9.2.3 Follow-up of AEs and SAEs....41
9.2.4 Regulatory reporting requirements for SAEs 41
9.2.5 Cardiovascular and death events 42
9.2.6 Disease-related events and/or disease-related outcomes not qualifying as AEs or SAEs 42
9.2.7 Pregnancy....42
9.2.8 Guidelines for reporting product complaints 42
9.3 TREATMENT OF OVERDOSE....42
9.4 SAFETY ASSESSMENTS 43
9.4.1 Physical examinations 43
9.4.2 Vital signs....43

9.4.3 Electrocardiograms 44
9.4.4 Clinical safety laboratory assessments 44

9.5 PHARMACOKINETICS 44

9.6 PHARMACODYNAMICS 47

9.7 GENETICS 47

9.8 BIOMARKERS 47

9.9 MEDICAL RESOURCE UTILIZATION AND HEALTH ECONOMICS 47

10 STATISTICAL CONSIDERATIONS 48

10.1 SAMPLE SIZE DETERMINATION 48

10.2 POPULATIONS FOR ANALYSES 48

10.3 STATISTICAL ANALYSES 49
10.3.1 Efficacy analyses 49
10.3.2 Safety analyses 51
10.3.3 PK analyses 53
10.3.4 Other analyses 53
10.3.5 Interim analyses 54

11 REFERENCES 55

12 APPENDICES 57

APPENDIX 1 ABBREVIATIONS 58

APPENDIX 2 CLINICAL LABORATORY TESTS 60

APPENDIX 3 STUDY GOVERNANCE CONSIDERATIONS 61

APPENDIX 4 ADVERSE EVENTS: DEFINITIONS AND PROCEDURES FOR RECORDING, EVALUATING, FOLLOW-UP, AND REPORTING 65

APPENDIX 5 CONTRACEPTIVE GUIDANCE AND COLLECTION OF PREGNANCY INFORMATION 70

APPENDIX 6 FORBIDDEN MEDICATION 73

APPENDIX 7 LIVER AND OTHER SAFETY: SUGGESTED ACTIONS AND FOLLOW-UP ASSESSMENTS 82

APPENDIX 8 SAMPLE SIZE DETERMINATION 90

APPENDIX 9 COUNTRY-SPECIFIC REQUIREMENTS 93

LIST OF TABLES

Table 1 - Objectives and endpoints ... 18
Table 2 - Overview of treatments administered ... 29
Table 3 – Numbering of PK samples ... 45
Table 4 – Summary of handling procedure of PK blood samples ... 45
Table 5 – Summary of handling procedure of PK plasma samples ... 46
Table 6 – Summary of PK bioanalytical methods ... 46
Table 7 - Populations for analyses ... 48
Table 8 - Efficacy analyses ... 49
Table 9 - Safety analyses ... 51
Table 10 - PK analyses ... 53
Table 11 - Protocol-required safety laboratory assessments ... 60
Table 12 - Highly effective contraceptive methods ... 71

LIST OF FIGURES

Figure 1 - Graphical study design ... 22

1 SYNOPSIS

Protocol title: A Randomized, Open label, Parallel-group, Single Dose Regimen, Phase 2a Study, to Investigate the Clinical and Parasiticidal Activity and the Pharmacokinetics of 3 dose levels of Artefenomel (OZ439) given in combination with Ferroquine (FQ) and FQ alone, in African Patients with Uncomplicated *Plasmodium falciparum* Malaria

Short title: A Randomized, Open, Parallel-group, Single Dose, Phase 2a Study, to Investigate the Clinical and Parasiticidal Activity and the Pharmacokinetics of 3 dose levels of Artefenomel in combination with Ferroquine (FQ) and FQ alone, in African Patients with Uncomplicated *Plasmodium falciparum* Malaria

Rationale:

Plasmodium falciparum (*P. falciparum*) malaria remains a deadly endemic parasitic disease, with 212 million new cases and over 429,000 deaths reported in 2015. World Health Organization (WHO) guidelines recommend that artemisinin-based combination therapies (ACT) are used to treat uncomplicated *P. falciparum* malaria, which is administrated in a 3-day regimen. Sanofi and Medicines for Malaria Venture (MMV) are co-developing a fixed dose combination (FDC) of FQ and artefenomel (OZ439) for a single dose treatment of uncomplicated malaria in adults and children. Per Food and Drug Administration (FDA) guidance for co-development of 2 new investigational drugs for use in combination, it is recommended to demonstrate the contribution of each individual component to the effect of the combination. This phase 2a study will test clinical and parasiticidal activity, pharmacokinetics (PK) and safety of 3 doses of OZ439 associated with a fixed dose of FQ and FQ alone.

Objectives and endpoints

Objective	Endpoint
**Primary**	
• To show the contribution of OZ439 to the clinical and parasiticidal effect of OZ439/FQ combination by analyzing exposure-response of OZ439 measured by Day 28 polymerase chain reaction (PCR)-corrected adequate clinical and parasitological response (ACPR) for the effect and the area under the concentration time curve up to infinity (AUC) of OZ439 as PK predictor	• Day 28 PCR-corrected ACPR
**Secondary**	
• To evaluate the dose response of OZ439 combined with FQ on PCR-corrected and crude Day 28 ACPR	• Day 28 PCR-corrected ACPR<br>• Day 28 crude ACPR
• To evaluate the dose-response of OZ439 combined with FQ on selected secondary endpoints	• Parasitemia at baseline then every 6 h during the first 36 h then at 48 h and every 24 h until Day 7<br>• Observed parasite reduction rate (PRR) at 24 h, 48 h, and 72 h

	• Time to 99% parasite reduction<br>• Time to parasite clearance<br>• Parasite clearance rate<br>• Time to recrudescence or re-infection<br>• time elapsed below limit of quantification (LOQ) of parasitemia
• To evaluate the safety and tolerability of different dosages of OZ439 in combination with FQ and FQ alone	• Adverse events (AEs) including Serious AEs (SAEs), AEs of Special Interest (AESIs) and Treatment Emergent AEs (TEAEs)<br>• Clinical laboratory tests (including liver function tests [LFT]), vital signs, and electrocardiogram (ECG) including QTc assessment<br>• Physical examination and clinical signs and symptoms related to uncomplicated *P. falciparum* malaria (fever, dizziness, headache, nausea, anorexia, vomiting, diarrhea, itching, urticaria, skin rash, abdominal pain, joint pain, muscle pain, palpitations, sleep problems, confusion, hearing problems, vision problems, and fatigue).
• To characterize the PK of OZ439 in plasma, and of FQ and its active metabolite SSR97213 in blood	• Concentrations of OZ439 in plasma, and of FQ and SSR97213 in blood at specific time points<br>• PK parameters for OZ439 in plasma, and for FQ and SSR97213 in blood, including:<br>- maximal concentration (C_{max})<br>- time to reach C_{max} (t_{max})<br>- concentration at 168 h post dose (C_{168h})<br>- AUC<br>- terminal half-life ($t_{1/2}$)<br>- area under the concentration time curve from T0 to Day 28 ($AUC_{0\text{-}Day28}$) for FQ and SSR97213 only

Overall design:

A phase 2a, randomized, open label, parallel-group study, single-dose regimen, testing 3 dose levels of OZ439 given in combination with FQ and FQ alone in participants with uncomplicated *P. falciparum* malaria. Adults up to 69 years old and adolescents as of 14 years old will be included in 4 parallel arms in a 1:1:1:1 randomization ratio. Randomization will occur the day of or up to 1 day after screening procedures.

Participants will receive one of the 4 treatments: FQ 400 mg alone or combined with OZ439 300 mg, 600 mg, or 1000 mg.

Participants will be hospitalized for the first 48 hours or up to 4 days or longer upon investigator's judgment.

Number of participants:

Participants will be screened to achieve 140 randomly assigned to study treatment and 120 evaluable participants for an estimated total of 30 evaluable participants per treatment group.

Details on sample size determination are provided in Section 10.1.

Evaluable participants are defined as all randomized participants with parasitologically confirmed malaria at baseline, who received the single administration of investigational medicinal product (IMP) and having parasitemia data post-randomization (Section 10.2).

Treatment groups and duration:

Study will last for up to 32 days for each participant:

- Up to 1 day screening period before the single-dose treatment
- 5 days of post-treatment surveillance, including 2 to 4 days hospitalization
- 24±2 days follow-up

Study treatments

Study treatments include FQ (all participants including arm A), OZ439 + FQ (participants of study arms B, C, and D), and a rescue therapy (all participants).

IMP: FQ (SSR97193)

- Formulation: 100 mg capsules
- Route of administration: oral
- Dose regimen: single dose of 400 mg given before OZ439

IMP: Artefenomel (OZ439)

- Formulation: sachet of 300 mg, 400 mg, and 600 mg of OZ439-alpha tocopherol polyethylene glycol 1000 succinate (TPGS) formulation + sachet of sucrose
- Route of administration: oral
- Dose regimen: single dose given after FQ, depending on the treatment arm: 0 mg for arm A, 300 mg for arm B, 600 mg for arm C, and 1000 mg for arm D

Noninvestigational medicinal products: rescue therapy

- A rescue treatment will be systematically administered at Day 28 for participants who reach Day 28 without having received a rescue medication
- The use of established anti-malarial drug combination per WHO recommendations and per local regulations and the choice of the best therapeutic option is left at investigator's decision.

Statistical considerations:

- **Primary analysis:** Logistic regression with p: probability of a response in PCR-corrected ACPR at Day 28 and OZ439 AUC and FQ $AUC_{0\text{-Day28}}$ (SSR97213 $AUC_{0\text{-Day28}}$ will be considered also) and baseline parasitemia as covariates will be fitted. The Wald test will be used to test the OZ439 exposure effect on PCR-corrected ACPR at Day 28.
- **Analysis of secondary endpoints:**
  - **Dose effect analysis**:

    A generalized multiple comparison procedures-modeling (MCP-Mod) approach will be used with PCR-corrected and crude ACPR at Day 28. Both linear and log model will be fitted. Then the dose response signal will be tested via contrasts based on dose-response shape.

    Clearance rate estimation according to worldwide antimalarial resistance network (WWARN) (1).
  - **Other activity endpoints:**

    Cox model will be used for time to event endpoints with OZ439 doses and parasitemia at baseline as covariates, potential interaction will be explored.

    Mean values or percentage of participants, per treatment arm, reaching each of the secondary endpoints, will be calculated according to the type of endpoint (quantitative or qualitative) with the corresponding 95% confidence interval (CI).

    Safety analyses will be descriptive.

    The on-treatment phase will be defined as the time of the first IMP administration (included) up to Day 28 (included).

    The PK parameters for different entities will be estimated through Bayesian analysis using previously developed population PK models.

    Descriptive statistics for OZ439, FQ and SSR97213 concentrations and PK parameters (C_{max}, t_{max}, C_{168h}, AUC, and $t_{1/2}$ for all entities, $AUC_{0\text{-Day28}}$ for FQ and SSR97213 only) will be provided.

2 SCHEDULE OF ACTIVITIES (SOA)

Procedure	Screening (up to 1 day before Day 0)	Treatment Period and post-treatment surveillance (Days)														Follow-up (Days)							Notes
		0[a]								1			2	3	4	5	6	7	10	14 ±1	21 ± 2	28 ±2	
		Hours post-dose																					
		0	1	2	4	6	8	12	18	24	30	36	48	72	96	120	144	168					
**Visit**	**1[b]**	**2**								**3**			**4**	**5**	**6**	**7**	**8**	**9**	**10**	**11**	**12**	**13[c]**	
Informed consent	X																						
Demography, medical history	X																						
Inclusion/ exclusion criteria	X																						
IRT call	X	X																				X	
Randomization		X																					
Hospitalization	←====================================→••••••••••••→																						All patients hospitalized for 48 h post dosing or up to 4 days in case malaria symptoms and/or parasitemia persist at the end of day 2 or longer upon investigator's judgment
<u>**Treatment**</u>																							
FQ		X																					Dosing in fasted condition (see Section 6.3.1)
OZ439		X																					
Rescue therapy[d]		← ••• ••• ••• ••• ••• ••• ••• ••• ••• ••• ••• ••• ••• ••• ••• ••• ••• ••																				X	
Prior and concomitant medication	X	X								X			X	X	X	X	X	X	X	X	X	X	

Procedure	Screening (up to 1 day before Day 0)	Treatment Period and post-treatment surveillance (Days)																	Follow-up (Days)				Notes
		0[a]								1			2	3	4	5	6	7	10	14 ±1	21 ± 2	28 ±2	
		Hours post-dose																					
		0	1	2	4	6	8	12	18	24	30	36	48	72	96	120	144	168					
**Visit**	1[b]	2								3			4	5	6	7	8	9	10	11	12	13[c]	
**Activity**																							
Physical exam & malaria signs & symptoms	X							X		X		X	X	(X)	(X)			X				X	Physical exam to be performed at discharge from the hospital
Height	X																						
Weight	X												(X)	(X)	(X)							X	Weight to be taken at discharge from hospital
Temperature (single)[e]	X		X	X		X		X	X	X	X	X	X	X	X	X	X	X	X	X	X	X	
Asexual & sexual parasite count (thick & thin blood films)[e]	X					X		X	X	X	X	X	X	X	X	X	X	X	X	X	X	X	Measurement required within 4 hours prior to dosing
qPCR, parasite genotyping, RT-qPCR gametocyte detection sampling[f]	X					X		X	X	X	X	X	X	X	X	X	X	X	X	X	X	X	
**Safety**																							
12-Lead ECG[g]	X	X		X	X	X	X	X		X			X	(X)	(X)			X					72 and 96 h post-dose if participant still hospitalized
Vital signs	X					X		X		X			X	(X)	(X)		X	X	X	X	X	X	Vital signs to be taken at discharge from the hospital
AEs	←==→																						
**Laboratory testing**																							
Clinical laboratory safety[h]	X												X	(X)	(X)			X		X	X	X	72 and 96 h post-dose if participant still hospitalized

Procedure	Screening (up to 1 day before Day 0)	Treatment Period and post-treatment surveillance (Days)														Follow-up (Days)							Notes
		0[a]								1			2	3	4	5	6	7	10	14 ±1	21 ± 2	28 ±2	
		Hours post-dose																					
		0	1	2	4	6	8	12	18	24	30	36	48	72	96	120	144	168					
**Visit**	1[b]	2								3			4	5	6	7	8	9	10	11	12	13[c]	
Viral hepatitis serology	X																						rapid spot test (HAV IgM, HBsAg, HCV Ab), and/or HCV RNA (if known)
Pregnancy test	X																					X	Urine beta-HCG is the minimum acceptable test. Result must be confirmed negative prior to dosing.
PK OZ439[j]		X	X	X	X	X		X		X			X	X		X		X		X			Not measured in Arm A; Plasma samples
PK FQ (+metabolite)[j]		X	X		X	X	X	X		X				X				X		X		X	Dry blood spot

a Day 0 is defined as the day of single dose treatment to comply with WHO guidelines (2).

b Visit 1 may occur on the same day as Visit 2 provided that all baseline assessments and biology results (including parasitemia and laboratory safety) are obtained before patient randomization.

c In case of study discontinuation, all assessments planned on Day 28, including rescue therapy administration, should be performed as far as possible.

d Administered at Day 28 for participants who reach Day 28 without having received a rescue medication. At each contact post-dosing, conditions for treatment failure will be assessed and rescue therapy will be given accordingly. See details in Section 7.7.1.

e Local thick and thin blood films performed at the site, before Informed Consent signature, according to local standard procedures and within 4 hours prior to dosing, can be used as screening/ pre-dose parasitemia assessments (Section 9.1.1.1) provided that a standard procedure is in place at site and blood films staining is performed according to the study operational manual. Blood films (thick and thin) and temperature measurements need to be confirmed as follows: when 1st parasite clearance and 1st temperature < 37.5 °C, measurements need to be confirmed with second reading 6 to 12 hours after the first measurement (ie, to determine Parasite Clearance and Fever Clearance). The first measurement (if confirmed) will be considered the 'Clearance Time'. Asexual and sexual counts will be measured separately: only asexual count will be used as parasitemia for evaluation of study endpoints. If parasites have not cleared within 72 hours after IMP administration and criteria for rescue medication are not met at 72 hours post-dose, blood films should continue to be taken according to site standard practice (or at minimum every 8 hours) until parasite clearance is shown or until criteria for rescue medication are met (see Section 7.7.1). If these additional blood smears are taken they should be recorded as unscheduled visits in the e-CRF. Axillary temperature should be recorded, if the axillary method is not possible, an alternative route (oral, tympanic, rectal) may be used. Within an individual participant the same method of temperature measure should be used throughout the study.

f RT-qPCR and qPCR will be performed at all parasitemia time-points (To be collected, according to the schedule and the instructions reported in the study operational manual). Parasite genotyping analysis will be performed on previously collected blood spot sample only in case of a positive blood film after initial parasite clearance: one pre-dose sample and one sample at 18 or 24 hours post dosing. A further sample will be analyzed at the time point at which recrudescence/re-infection occurs (if applicable).

g Single local ECG reading for inclusion purpose and triplicate central ECG reading for safety evaluation during study.

h Laboratory safety will be performed locally: hematology, clinical chemistry, and urinalysis (see Appendix 2 for details).

i Where timepoints coincide, the PK samples should be taken after measurement of vital signs, temperature, and ECG. A PK sample (OZ439 and FQ) should be obtained if possible when QTcF >500 ms or QTcF is prolonged by >60 ms from baseline, or when increased ALT is measured (see Section 9.2). PK samples numbering is detailed in Table 3.

AE: adverse event; e-CRF: electronic case report form; ECG: electrocardiogram; FQ: ferroquine; HAV IgM: hepatitis A immunoglobulin M; HBsAg: hepatitis B surface antigen; HCG: human chorionic gonadotropin; HCV Ab: hepatitis C virus antibody; HCV RNA: hepatitis C virus ribonucleic acid; IMP: investigational medicinal product; IRT: Interactive response technology; PK: pharmacokinetics; qPCR: quantitative polymerase chain reaction; RT-qPCR: quantitative reverse transcription polymerase chain reaction; WHO: World Health Organization

3 INTRODUCTION

Sanofi and MMV are co-developing an FDC of FQ (investigational new drug IND-115244) and artefenomel (OZ439) (IND-104549) for a single dose treatment of uncomplicated malaria in adults and children.

OZ439 is a novel, synthetic trioxolane that shows promising parasiticidal activity as a peroxidic anti-malarial agent. Refer to the investigator's brochure (IB) (3) for details.

Ferroquine is a new 4-aminoquinoline analogue (ferrocenyl derivative of chloroquine) active against chloroquine-resistant and sensitive *P. falciparum* strains. Ferroquine is metabolized into one major metabolite (N-demethyl derivative, SSR97213), equally active in vitro on chloroquine-sensitive strain.

3.1 STUDY RATIONALE

When the clinical development of OZ439/FQ will be completed with phase 3 studies, Sanofi and MMV intend to submit a new drug application (NDA) for registration at United States Food and Drug Administration (USFDA).

Per FDA guidance (4) for co-development of 2 new investigational drugs or new chemical entities for use in combination, it is recommended to demonstrate the contribution of each individual component to the effect of the combination.

Contribution of FQ to the combination will be demonstrated in the ongoing OZ439/FQ phase 2b trial (DRI12805) in which clinical efficacy of 4 doses of FQ (400, 600, 900 and 1200 mg) associated with a fixed dose of 800 mg of OZ439 will be analyzed using an efficacy endpoint to show a dose effect.

This phase 2a study will evaluate the contribution of OZ439 to the effect of the combination by testing clinical and parasiticidal activity, PK and safety of 3 doses of OZ439 associated with a fixed dose of FQ and FQ alone in African adults and adolescents as of 14 years of age and affected with uncomplicated *P. falciparum* malaria. The reason for not including younger children in the study is to avoid administering sub-therapeutic treatment to a young population that is more susceptible to severe malaria. In addition, it is common to perform an activity phase 2a study in an adult population.

The primary objective of the study will be to show the contribution of OZ439 to the clinical and parasiticidal effect of OZ439/FQ combination by analyzing exposure-response of OZ439 measured by Day 28 PCR-corrected ACPR for the effect and the AUC of OZ439 as PK predictor.

Provided that the 400 mg FQ dose administered to all patients in this study is the minimal dose tested in the Phase 2b study (DRI12805) and that the present study will investigate the role of OZ439, a drug with a short/mid half-life, in the activity of the drug association, a 28 days follow-up may be sufficient to properly evaluate the activity while ensuring good safety assessment.

3.2 BACKGROUND

Despite a decline of 41% of malaria incidence rate and 62% of malaria mortality from 2000 to 2015, *P. falciparum* malaria remains a deadly endemic parasitic disease, with 212 million new cases reported in 2015. *P. falciparum* malaria killed over 429,000 people in 2015, 92% of them living in Africa and 70% being children less than 5 years of age (5). Malaria is curable and preventable: principal control strategies including rapid diagnosis, effective treatment and personal protection with bed nets. World Health Organization guidelines (6) recommend that ACTs are used to treat uncomplicated *P. falciparum* malaria to counter the threat of resistance of *P. falciparum* to monotherapies and to improve treatment outcome. Artemisinin derivatives produce rapid clearance of parasitemia and resolution of symptoms and needs a 3 day regimen when given in combination with slowly eliminated anti-malarial drugs such as 4-aminoquinoline drugs. With this shorter 3-day course, the complete clearance of all parasites is dependent on the partner medicine being effective and persisting at parasiticidal concentrations until all infecting parasites have been killed.

However evidence suggests that access to ACTs and compliance to the 3-day treatment course is not optimal, particularly in children (7). A once daily dosing has been proven to offer improved adherence as compared to twice daily regimen (5), while 3 days ACTs have improved adherence as comparted to longer regimens used before (6). A single dose antimalarial treatment could offer, for the first time, the realistic potential for optimal adherence.

In addition, evidence of emergence of potential plasmodial resistance to artemisinin (8,9,10,11,12,13) and more recently piperaquine phosphate (PQP) (14,15) suggests that resistance to ACT components may become an issue in the coming years. Thus, new drug combinations with new mode of action are required to treat sensitive and resistant genotypes of *P. falciparum*, and effective single exposure combination treatments in particular are likely to lead to significant improvement in real-life effectiveness.

Ferroquine has been tested on various clinical isolates (chloroquine-sensitive, chloroquine-resistant, artemisinin and PQP resistant and multi-drug resistant), from South East Asia and from Africa, and displayed a very potent antimalarial activity against *P. falciparum* with no cross-resistance with other anti-malaria drugs. Refer to the FQ IB (16) for details.

3.3 BENEFIT/RISK ASSESSMENT

The benefit and risk assessment of OZ439/FQ administered in combination in the Phase 2a ACT14655 study and the safety evaluation are based on nonclinical data and on clinical data from completed studies as of cut-off date of the referring respective IBs.

The primary AEs reported were mainly gastrointestinal (nausea, vomiting) and nervous system (headache, dizziness) events. However, based on nonclinical and clinical data analyses of OZ439 and FQ, hepatic effect (elevated liver enzymes) and proarrhythmic effect (QT interval corrected using Fridericia formula [QTcF] prolongation) are the identified risks for the individual components OZ439 and FQ given alone or in combination. Participants will be closely monitored for hepatic and cardiac conduction effects. Since this is an investigational study designed to

(electronic 3.0)

determine the contribution of OZ439 to the effect of the combination OZ439/FQ, participants enrolled in some of the 4 arms of the study might receive sub-therapeutic drug treatment. This risk will be properly mitigated by the fact that only adults and adolescents as of 14 years of age and presenting with uncomplicated *P. falciparum* malaria are eligible, and that participants will be hospitalized in a well-equipped health center for at least 48 h or up to 4 days in case malaria symptoms and/or parasitemia persist, or longer upon investigator’s judgment. Participants will receive a standard rescue therapy as soon as there is evidence of treatment failure or systematically on Day 28 for the ones who reach Day 28 without having received a rescue medication.

Justification for the selected doses of IMP is provided in Section 5.5.

More detailed information about the known and expected benefits and risks and reasonably expected AEs of OZ439 and FQ may be found in the respective IBs (3, 16).

4 OBJECTIVES AND ENDPOINTS

Table 1 - Objectives and endpoints

Objectives	Endpoints
**Primary**	
• To show the contribution of OZ439 to the clinical and parasiticidal effect of OZ439/FQ combination by analyzing exposure-response of OZ439 measured by Day 28 PCR-corrected ACPR for the effect and the AUC of OZ439 as PK predictor	• Day 28 PCR-corrected ACPR (see Section 10.3.1.1 for details)
**Secondary**	
• To evaluate the dose response of OZ439 combined with FQ on PCR-corrected and crude Day 28 ACPR	• Day 28 PCR-corrected ACPR (see Section 10.3.1.1 for details)<br>• Day 28 crude ACPR (see Section 10.3.1.1 for details)
• To evaluate the dose-response of OZ439 combined with FQ on selected secondary endpoints	• Parasitemia (/µl) at baseline then every 6 h during the first 36 h then at 48 h and every 24 h until Day 7<br>• observed PRR at 24 h, 48 h, and 72 h<br>• time (h) to 99% parasite reduction<br>• time (h) to parasite clearance<br>• Parasite clearance rate<br>• time (h) to recrudescence or re-infection<br>• time (h) elapsed below LOQ of parasitemia
• To evaluate the safety and tolerability of different dosages of OZ439 in combination with FQ and FQ alone	• AEs including SAEs, AESIs and TEAEs<br>• Clinical laboratory tests (including LFT), vital signs, and ECG including QTc assessment<br>• Physical examination and clinical signs and symptoms related to uncomplicated *P. falciparum* malaria (fever, dizziness, headache, nausea, anorexia, vomiting, diarrhea, itching, urticaria, skin rash,

Objectives	Endpoints
	abdominal pain, joint pain, muscle pain, palpitations, sleep problems, confusion, hearing problems, vision problems, and fatigue).
• To characterize the PK of OZ439 in plasma, and of FQ and its active metabolite SSR97213 in blood	• Concentrations of OZ439 in plasma, and of FQ and SSR97213 in blood at specific time points<br>• PK parameters for OZ439 in plasma, and for FQ and SSR97213 in blood, including:<br>- C_{max}<br>- t_{max}<br>- C_{168h}<br>- AUC<br>- $t_{1/2}$<br>• $AUC_{0\text{-Day 28}}$ for FQ and SSR97213 only
**Tertiary/exploratory**	
• To explore the impact of study treatment on functional gametocytemia	• Quantitative reverse transcription Polymerase Chain Reaction (RT-qPCR) to measure gametocyte maturation stage (expression of *Pfs*25 mRNA)
• To evaluate the relationship between parasitemia and concentration of OZ439/FQ	• Quantitative Polymerase Chain Reaction (qPCR) and RT-qPCR to explore, at all parasitemia time-points, the PK/PD of OZ439/FQ (to be reported in a separate report).
• To evaluate the relationship between QTc and concentration of OZ439/FQ	• QTcF to explore, at all ECG time-points, the PK/PD of OZ439/FQ

4.1 APPROPRIATENESS OF MEASUREMENTS

Day 28 PCR-corrected ACPR is an appropriate measure of the clinical and parasitological activity of the OZ439/FQ combination on the *P. falciparum* infection, reflecting both early parasite clearance and long lasting sterilization along a 28 days period.

A previous exposure-response analysis of data from MMV_OZ439_13_003 has demonstrated that both OZ439 and PQP contributed to the overall efficacy of the combination (17). Then, exposure (AUC) has been selected as PK predictor in the primary analysis based on previous internal data

(from MMV in the context of OZ439/PQP and from sanofi in the context of FQ/artesunate) indicative that it is a good predictive marker of parasiticidal activity (similar to C_{168h}).

At this stage of the clinical development, the effective dose of OZ439 and FQ selected to be tested in large scale phase 3 trials is unknown and the range of exposure and effect associated with the 3 individual selected OZ439 doses cannot be anticipated.

Modeling and simulations (M&S) show that OZ439 exposure-effect analysis may be a proper way to analyze and conclude on OZ439 contribution as well as dose-effect analysis, while needing fewer participants and maintaining an appropriate statistical power. A PK/PD model will be built specifically for OZ439/FQ combination. A similar model was used in an OZ439/PQP phase 2b study and it demonstrated the contribution of OZ439 in the OZ439/PQP combination using the same endpoint and analysis method (17).

Secondary objectives of the study will evaluate the dose-response of OZ439 on Day 28 PCR corrected ACPR and selected secondary activity endpoints (time to parasite clearance, time to 99% parasite reduction, ratio of participants with parasitemia at Day 4, observed PRR, estimated parasite clearance rate (1), time to recrudescence or re-infection, time LOQ of parasitemia, Day 28 crude ACPR). Secondary objectives will also evaluate the safety and tolerability of OZ439/FQ combination with dose ranging of OZ439 and will characterize the PK of OZ439 in plasma, and of FQ and its active metabolite SSR97213 in blood.

5 STUDY DESIGN

5.1 OVERALL DESIGN

This is a phase 2a, randomized, open label, parallel-group study, single-dose regimen, testing 3 dose levels of OZ439 given in combination with FQ and FQ alone in patients with uncomplicated *P. falciparum* malaria.

The study will be organized in up to 10 sites in Sub Saharan African countries with malaria transmission. Adults up to 69 years old and adolescents as of 14 years old will be included in 4 parallel arms in a 1:1:1:1 randomization ratio.

Participants will receive one of the 4 combinations: FQ 400 mg alone or combined with OZ439 300 mg, 600 mg or 1000 mg. Doses will not be adjusted to participant weight.

Participants will be hospitalized for the first 48 hours or up to 4 days in case malaria symptoms and/or parasitemia persist, or longer upon investigator's judgment. Following discharge, participants will return to the site for further assessments up to Day 28. A rescue treatment will be systematically administered on Day 28 for participants who reach Day 28 without having received a rescue medication.

Study will last for up to 32 days for each participant:

- Up to 1 day screening period before the single-dose treatment
- 5 days of post-treatment surveillance, including 2 to 4 days hospitalization
- 24±2 days follow-up

Figure 1 - Graphical study design

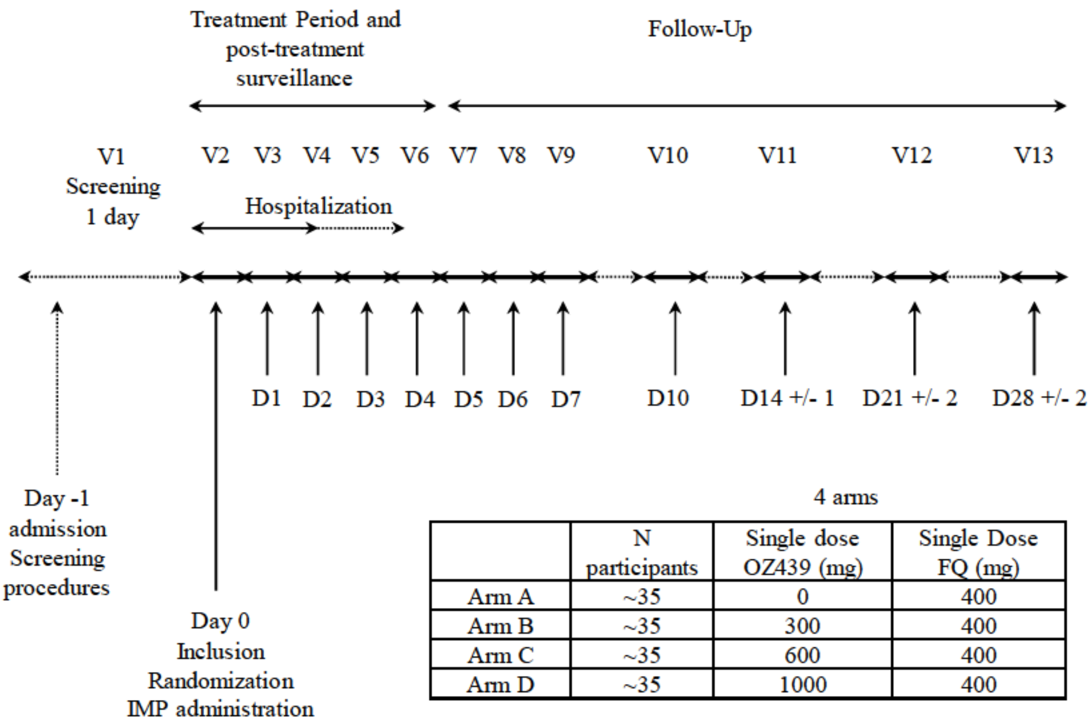

	N participants	Single dose OZ439 (mg)	Single Dose FQ (mg)
Arm A	~35	0	400
Arm B	~35	300	400
Arm C	~35	600	400
Arm D	~35	1000	400

Note: Visits 1 & 2 can occur on the same day ; Visit 13 : assessment of primary endpoint

IMP: Investigational Medicinal Product

5.2 PARTICIPANT AND STUDY COMPLETION

Participants will be screened to achieve 140 randomly assigned to study treatment and 120 evaluable participants for an estimated total of 30 evaluable participants per treatment group.

Details on sample size determination are provided in Section 10.1.

Evaluable participants are defined as all randomized participants with parasitologically confirmed malaria at baseline, who received the single administration of IMP and who have parasitemia data post-randomization (Section 10.2).

5.3 END OF STUDY DEFINITION

A participant is considered to have completed the study if he/she has completed all phases of the study including the last visit (ie, Day 28).

The end of the study is defined as the date of the last visit of the last participant in the study.

5.4 SCIENTIFIC RATIONALE FOR STUDY DESIGN

There are 3 aspects to consider to define most appropriate baseline parasitemia for the selection of the participants in the study:

- Baseline parasitemia is a strong covariate of parasiticidal activity and clinical efficacy. A phase 2b study testing OZ439 and PQP performed by MMV (MMV_OZ439_13_003) showed that baseline parasitemia is strongly correlated with clinical efficacy (Day 28 PCR-corrected ACPR), meaning that efficacy may increase when baseline parasitemia decreases, making more difficult to show a significant difference in efficacy across study arms. On the other hand increasing baseline parasitemia may not facilitate the recruitment of adult participants living in endemic places.
- There is also a need to minimize the risk of re-infection during the trial in participants receiving FQ 400 mg to keep sufficient evaluable patients in each arm while allowing to enroll 140 patients in a reasonable timeframe and discriminating levels of activity across study groups. A baseline parasitemia of ≥3000 parasites/µl is a good compromise that would meet both requisites and allow participants recruitment in places with moderate transmission and low risk of re-infection during the study.
- A baseline parasitemia ≥3000 parasites/µl is deemed to enlarge candidate site list, accelerate participant recruitment and minimize risk of re-infection during the study while keeping an adequate power. It will also minimize the risk of progression to severe malaria in a context of low dose of OZ439 in some groups.

5.5 JUSTIFICATION FOR DOSE

Malaria therapy is constrained by WHO's guidance that is recommending to treat an uncomplicated *P. falciparum* malaria with a combination of 2 highly potent and active drugs having a different mode of action with the objective to treat the infection and prevent resistance to the drugs (6).

The efficient minimal dose of FQ will be confirmed in the ongoing phase 2b trial (DRI12805). However, there is some evidence that FQ is a highly potent drug. Therefore it may be difficult to show a significant effect ranging of OZ439 within the combination if the FQ dose level is too high.

In this study, a dose of 400 mg of FQ was selected (this is one of the doses tested in the DRI12805). This dose is low enough to leave room for added efficacy attributed to OZ439. This dose will minimize the risk to see FQ effect hiding OZ439 effect and optimize the chance to show an effect ranging across the 4 groups tested in this study. Modeling and simulations indicate that efficacy of FQ 400 mg when given alone (PCR corrected Day 28 ACPR) may be around 72%, leaving room for increased efficacy related to OZ439 to less than 20%.

Participants enrolled in some of the 4 groups of the study might receive sub-therapeutic dose of drug combination, however only adults and adolescents as of 14 years of age and presenting with uncomplicated malaria with ≤50,000 asexual parasites/µL of blood will be enrolled, both measures mitigating the risk of development of a severe malaria. They will be hospitalized for at

least 48 h in a well-equipped health center and will receive a standard rescue therapy as soon as there is evidence of treatment failure. In addition to initial hospitalization, patients will have frequent assessments (biweekly after the hospitalization through Day14 and weekly thereafter through the end of the study), and they will receive a rescue therapy at Day 28 even without signs of treatment failure. All these measures will mitigate the risk of malaria management failure.

To optimize the likelihood to observe a difference on the primary endpoint (Day 28 PCR corrected ACPR) over the various doses of OZ439, FQ 400 mg will be administered alone or with OZ439 at a dose of 300, 600 or 1000 mg, while OZ439 800 mg is being tested in the ongoing OZ439/FQ DRI12805 phase 2b study.

The rationale to include a group receiving 1000 mg of OZ439 is based on:

- OZ439 was tested in phase 2a studies up to a single dose of 1200 mg (powder in bottle formulation with a glass of milk), with no particular safety issue,
- Increasing the range of effect across the 4 study groups. M&S show that including extreme doses (0 and 1000 mg) will help. This is also supported by FDA written response to Type C meeting dated August 2015, that suggested to consider an OZ439 dose higher than 800 mg to optimize the effect ranging,
- Modeling and simulation show a significant exposure-effect with good power and limited number of participants,
- An interest in framing the 800 mg OZ439 dose that is being tested in the Phase 2b, to comfort the assumption that this will be the optimal dose to carry forward into phase 3 trials.

6 STUDY POPULATION

Prospective approval of protocol deviations to recruitment and enrollment criteria, also known as protocol waivers or exemptions, is not permitted.

6.1 INCLUSION CRITERIA

Participants are eligible to be included in the study only if all of the following criteria apply:

Age

I 01. Participant must be 14 to 69 years of age inclusive, at the time of signing the informed consent.

Type of participant and disease characteristics

I 02. Participants who present with uncomplicated *P. falciparum* malaria, with a fever as defined with axillary temperature ≥37.5 degree Celsius (°C) or oral/ rectal/ tympanic temperature ≥38°C or history of fever in the previous 24 hours (history of fever must be documented), with a mono-infection with *P. falciparum* and parasitemia (microscopically, blood smear) ≥3,000 and ≤50,000 asexual parasites/µL of blood.

Weight

I 03. Body weight within 35 and 90 kg (inclusive).

Sex

I 04. Male and female

a) Male participants: A male participant must agree to use contraception as detailed in Appendix 5 of this protocol for 3 months after the last dose of study treatment and refrain from donating sperm during this period.

b) Female participants: A female participant is eligible to participate if she is not pregnant (see Appendix 5), not breastfeeding, and at least one of the following conditions applies:

- Not a woman of childbearing potential (WOCBP) as defined in Appendix 5

  OR

- A WOCBP who agrees to follow the contraceptive guidance in Appendix 5 for 3 months after the last dose of study treatment.

Informed Consent

I 05. Capable of giving signed informed consent (if the patient is ≥age defining majority) as described in Appendix 3, which includes compliance with the requirements and restrictions listed in the informed consent form (ICF) and in this protocol. Informed consent signed by the legally acceptable representative (LAR) of the minor patient (<18 years of age or <other age locally defining majority). In addition, in accordance with local regulation, minor participants with capacity for writing could sign off on an Assent Form. For those who are able to understand but with no capacity for writing the Assent Form would be read. In that case, an impartial witness could certify the document was read to the child.

6.2 EXCLUSION CRITERIA

Participants are excluded from the study if any of the following criteria apply:

Medical conditions

E 01. Presence of severe malaria (6, 7)

E 02. Known history or evidence of clinically significant gastrointestinal, cardiovascular, hepatic, renal, hematological, respiratory, endocrine, immunological, infectious, neurological (in particular convulsions), malignancy, psychiatric disease or symptoms which, in the judgment of the investigator, might confuse the interpretation of the safety information

E 03. Severe vomiting defined as more than 3 times in the 24 hours prior to enrollment in the study or inability to tolerate oral treatment or severe diarrhea defined as 3 or more watery stools per day

E 04. Severe malnutrition defined as a body mass index of less than 16 kg/m^2 (18) for adults and for children Z-score <-3 or weight for age (%) of the median <60

E 05. Splenectomized participants or presence of surgical scar on left hypochondrium

E 06. Known history of hypersensitivity, allergic, or anaphylactoid reactions to FQ or other amino quinolines or to OZ439 or OZ277 or any of the excipients

Prior/concomitant therapy

E 07. Participant treated with anti-malarial treatment:

- With PQP-based compound, mefloquine, naphthoquine or sulphadoxine/pyrimethamine within the previous 6 weeks.
- With amodiaquine or chloroquine within the previous 4 weeks.
- With quinine, halofantrine, lumefantrine-based compounds and any other anti-malarial treatment or antibiotics with antimalarial activity (including cotrimoxazole,

(electronic 3.0)

tetracyclines, quinolones and fluoroquinolones, and azithromycin) within the past 14 days.

- With any herbal products or traditional medicines, within the past 7 days

E 08. Previous treatment within 5 times the half-life or within the last 14 days, whichever the longest, which are: strong CYP2C or CYP3A inhibitors and/or moderate inhibitors but inhibiting both CYP2C and CYP3A and/or CYP inducers (Appendix 6)

E 09. Any treatment known to induce a prolongation of QT interval (Appendix 6)

Prior/concurrent clinical study experience

E 10. Participated in any trial investigating OZ439 and/or FQ compounds

E 11. Previous participation in any malaria vaccine study or received malaria vaccine in any other circumstance

E 12. Enrolled in another clinical trial within the past 4 weeks or during the study period

Diagnostic assessments

E 13. Mixed *Plasmodium* infection

E 14. Presence of Hepatitis A – immunoglobulin M (HAV-IgM), Hepatitis B surface antigen (HBs Ag) or Hepatitis C virus antibody (HCV Ab) and/or known to have active Hepatitis C virus RNA (HCV RNA)

E 15. Laboratory parameters with abnormalities deemed clinically significant by the investigator.

E 16. Abnormal LFT: aspartate transferase (AST) >2 upper limit of normal range (ULN), or alanine transferase (ALT) >2 ULN or total bilirubin >1.5 ULN

E 17. Positive pregnancy test at study screening for female participants of childbearing potential

E 18. QTcF >450 ms at screening or pre-dose

E 19. Hypokalemia (<3.5 mmol/L), hypocalcemia (<2.0 mmol/L) or hypomagnesemia (<0.5 mmol/L) at screening or pre-dose

Other exclusions

E 20. Any country-related specific regulation that would prevent the subject from entering the study - see Appendix 9 (country specific requirements)

E 21. Family history of sudden death or of congenital prolongation of the QT interval or known congenital prolongation of the QT-interval or any clinical condition known to prolong the QT interval e.g., participants with a history of symptomatic cardiac arrhythmias including atrial fibrillation (AF) or with clinically relevant bradycardia

E 22. Patient not suitable for participation, whatever the reason, as judged by the Investigator, including medical or clinical conditions, or patients potentially at risk of noncompliance to study procedures or unable to drink

6.3 LIFESTYLE RESTRICTIONS

6.3.1 Meals and dietary restrictions

1. No food or milk is allowed within 3 hours before dosing and until 2 hours after dosing.
2. No water is allowed until 2 hours after dosing, after which time, water is allowed ad libitum.

6.4 SCREEN FAILURES

Screen failures are defined as participants who consent to participate in the clinical study but are not subsequently randomly assigned to study treatment. A minimal set of screen failure information is required to ensure transparent reporting of screen failure participants to meet the Consolidated Standards of Reporting Trials (CONSORT) publishing requirements and to respond to queries from regulatory authorities. Minimal information includes demography, screen failure details, eligibility criteria, and any SAE.

Individuals who do not meet the criteria for participation in this study (screen failure) may be rescreened only for a subsequent malaria episode.

7 TREATMENTS

Study treatment is defined as any investigational treatment(s), marketed product(s), placebo, or medical device(s) intended to be administered to a study participant according to the study protocol.

7.1 TREATMENTS ADMINISTERED

Table 2 - Overview of treatments administered

Study treatment name	Arm A	Arm B	Arm C	Arm D
Dosage formulation	FQ	FQ + OZ439	FQ + OZ439	FQ + OZ439
Dosage level : FQ	400 mg	400 mg	400 mg	400 mg
OZ439	-	300 mg	600 mg	1000 mg
Route of administration	oral	oral	oral	oral
Dosing instructions	Single dose<br>Fasted condition	Single dose<br>Fasted condition<br>FQ then OZ439	Single dose<br>Fasted condition<br>FQ then OZ439	Single dose<br>Fasted condition<br>FQ then OZ439
Packaging and labeling	Study treatment will be provided in open label treatment box. Each box will be labeled as required per country requirement. Packaging is in accordance with the administration schedule.			
Content of each box:	1 wallet of FQ 400 mg	1 wallet of 400 mg FQ, 1 sachet of 300 mg OZ439 +TPGS, and 1 sachet of 5.625 g sucrose	1 wallet of 400 mg FQ, 1 sachet of 600 mg OZ439 +TPGS, and 1 sachet of 11.25 g sucrose	1 wallet of 400 mg FQ, 1 sachet of 400 mg OZ439 +TPGS, 1 sachet of 600 mg OZ439 +TPGS, and 1 sachet of 18.75 mg sucrose
Manufacturer	SANOFI Montpellier	SANOFI Montpellier	SANOFI Montpellier	SANOFI Montpellier

7.1.1 Investigational Medicinal Products

All details regarding the preparation of IMPs, including suspension OZ439, will be described in the operational manual.

7.1.1.1 Ferroquine

A combination of the 100 mg FQ capsules will be used to compose the 400 mg FQ single dose. FQ will be administered orally on Day 0 in fasted condition (see Section 6.3.1 for details).

No re-dosing of FQ will be performed if a patient vomits during or after FQ administration but before OZ439 administration. In that case, OZ439 will not be administered and the patient will receive a rescue therapy as per Section 7.7.1.

7.1.1.2 OZ439

Individual sachets contain OZ439 granules (dose strengths 300, 400 and 600 mg) and TPGS granules for oral suspension. Sucrose granules for oral suspension will be provided in separate sachets and will be added to the OZ439 + TPGS aqueous oral suspension to make it palatable.

OZ439 will be administered orally after the FQ administration. The suspension will have to be drunk within 2 hours and 20 minutes of the start of its preparation, as quickly as possible and over a maximum of 30-minute period.

- If a participant vomits within 5 minutes of the start of dosing with OZ439, they will be re-dosed with a freshly prepared new suspension from a new individual patient treatment kit assigned via interactive response technology (IRT). No re-dosing of FQ will be performed.
  - If the participant vomits again within 35 minutes after re-dosing, they will receive a rescue therapy as per Section 7.7.1.
- If vomiting occurs from 5 minutes to 35 minutes post start of OZ439, they will continue to take the OZ439 dose (if any left), but will not be re-dosed.
- If a patient vomits after 35 minutes from the start of OZ439 dosing, they will not be re-dosed.

7.1.2 Noninvestigational Medicinal Products

Rescue therapy will be administered to all participants, either before Day 28 if demanded by the participants health status (see Section 7.7.1 for details), or at Day 28 for participants who reach Day 28 without treatment failure.

Rescue therapy consists of established anti-malarial drug combination per WHO recommendations (6) and per local regulations and the choice of the best therapeutic option is left at investigator's decision.

7.2 DOSE MODIFICATION

Not applicable: this is a single-dose study.

7.3 METHOD OF TREATMENT ASSIGNMENT

The randomized treatment kit number list will be generated centrally by Sanofi. The IMPs will be packaged in accordance with this list.

Participants will be randomized to one of the treatment arms via a centralized randomization system using IRT. A participant will be considered randomized when the treatment number has been provided by the IRT, as documented in the IRT log file.

At the screening visit (Visit 1), the site will contact the IRT to obtain a participant number for each participant who gave an informed consent. The participant number will be associated with the center and allocated in chronological order in each center.

IRT will manage one participant randomization list and allocate the treatment number and the corresponding treatment kit to the participant.

Approximately 140 participants will be randomized to one of the 4 treatment arms of OZ439/FQ. The randomization ratio is 1:1:1:1 with FQ fixed at 400 mg and OZ439 doses will be 0, 300, 600 and 1000 mg.

A participant can be randomized only once.

Centralized randomization and IRT-related procedures will be detailed in the study operational manual.

7.4 BLINDING

Not applicable since this is an open label study using central randomization via IRT; the specific treatment to be taken by a participant will be assigned using IRT. The site will contact IRT prior to the start of study treatment administration for each participant.

7.5 PREPARATION/HANDLING/STORAGE/ACCOUNTABILITY

All details regarding the preparation of IMP will be described in the study operational manual.

1. The investigator or designee must confirm appropriate temperature conditions have been maintained during transit for all study treatment received and any discrepancies are reported and resolved before use of the study treatment.
2. Only participants enrolled in the study may receive study treatment and only authorized site staff may supply or administer study treatment. All study treatments must be stored in a secure, environmentally controlled, and monitored (manual or automated) area with access limited to the investigator and authorized site staff in accordance with local regulations, labeled storage conditions, policies, and procedures.
3. The investigator, institution, or the head of the medical institution (where applicable) is responsible for study treatment accountability, reconciliation, and record maintenance (ie, receipt, reconciliation, and final disposition records).
4. Further guidance and information for the final disposition of unused study treatment are provided in the study operational manual.

Any quality issue noticed with the receipt or use of an IMP/NIMP (deficiency in condition, appearance, pertaining documentation, labeling, expiration date, etc) must be promptly notified to the Sponsor. Some deficiencies may be recorded through a complaint procedure (see Section 9.2.8).

A potential defect in the quality of IMP/NIMP may be subject to initiation of a recall procedure by the Sponsor. In this case, the Investigator will be responsible for promptly addressing any request made by the Sponsor, in order to recall the IMP/NIMP and eliminate potential hazards.

Under no circumstances will the Investigator supply IMP/NIMP to a third party, allow the IMP/NIMP to be used other than as directed by this clinical trial protocol, or dispose of IMP/NIMP in any other manner.

7.6 TREATMENT COMPLIANCE

Measures taken to ensure and document treatment compliance and IMP accountability include:

- The investigator or his/her delegate among the study personnel will monitor the administration of IMP and the IMP uptake will be recorded in the electronic case report form (e-CRF)
- Proper recording of treatment kit number or packaging number as required on appropriate e-CRF page for accounting purposes
- The pharmacist or designee will prepare the kit assigned by IRT, will complete the corresponding treatment log form, and will perform reconciliation by checking the consistency of the treatment number between IRT assignment notification, the treatment number printed on the kit boxes and the treatment log forms
- IMP will also be recorded and tracked on the site IMP inventory forms
- The monitor in charge of the study will check the data entered on the e-CRF by comparing them with the IMP that has been retrieved and the treatment log form
- A detailed treatment log of the destroyed IMP will be established with the Investigator (or the pharmacist) and countersigned by the Investigator and the monitoring team. The Investigator will not destroy the unused IMP unless the Sponsor provides written authorization

7.7 CONCOMITANT THERAPY

All drugs other than oral contraceptives, paracetamol (acetaminophen) at the maximum dose of 40 mg/kg/day, metoclopramide if repeated vomiting and beta-lactams for any infection needing an antibiotic treatment are prohibited over the entire study period unless medically justified. The dosage and number of dose(s) will be recorded. The use of other contraceptive methods may be discussed. Traditional and herbal remedies are not permitted during the study.

Appendix 6 is listing drugs that can interfere with OZ439 or FQ metabolism (strong cytochrome P450 [CYP] 2C or CYP3A inhibitors, moderate inhibitors inhibiting both CYP2C and CYP3A, and CYP inducers) or can be affected by OZ439 and FQ in their metabolism (CYP2D6 main substrates, P-glycoprotein [P-gp] substrate) and drugs that can increase QT interval.

Any medication or vaccine (including over-the-counter or prescription medicines, vitamins, and/or herbal supplements) that the participant is receiving at the time of enrollment or receives during the study must be recorded along with:

- Reason for use
- Dates of administration including start and end dates
- Dosage information including dose and frequency

The Medical Monitor should be contacted if there are any questions regarding concomitant or prior therapy.

7.7.1 Rescue medicine

The study site will supply rescue medication that will be obtained locally upon local recommendation.

The use of established anti-malarial drug combination per WHO recommendations (6) and per local regulations and the choice of the best therapeutic option is left at investigator's decision.

The use of rescue medications is allowed as soon as

- vomiting occurs during FQ dosing or after FQ dosing but before OZ439 administration,
- vomiting occurs within 35 minutes after OZ439 re-dosing (see Section 7.1.1.2), or
- there is evidence of treatment failure (see definition below).

If no rescue medication is administered before Day 28, it will be administered on Day 28.

Treatment failure is defined according to WHO recommendations (2) as follows:

Early treatment failure (one the following)

- danger signs or severe malaria on Day 1, 2, or 3 in the presence of parasitemia;
- parasite count on Day 2 higher than on Day 0, irrespective of axillary temperature;
- parasitemia on Day 3 with axillary temperature ≥37.5°C;
- parasite count on Day 3 ≥25% on Day 0.

Late clinical failure

- danger signs or severe malaria on any day between Day 4 and Day 28 in the presence of parasitemia, without previously meeting any of the criteria of early treatment failure;
- presence of parasitemia and axillary temperature ≥37.5°C (or history of fever) on any day between Day 4 and Day 28, without previously meeting any of the criteria of early treatment failure.

Late parasitological failure

- presence of parasitemia on any day between Day 7 and Day 28 and axillary temperature <37.5°C, without previously meeting any of the criteria of early treatment failure or late clinical failure.

Before giving a rescue therapy before Day 28, where possible:

- a PK (OZ439 and FQ) sample should be taken
- blood films, parasite genotyping, RT-qPCR, and qPCR must be taken

Following administration of rescue therapy before Day 28, patients should be managed according to local standard of care practices. Safety data, parasitemia (blood films), and PK data should continue to be collected in the e-CRF, according to the study schedule, and up to the end of the study (Day 28) even if re-emergence is confirmed.

7.8 TREATMENT AFTER THE END OF THE STUDY

No treatment will be provided after the end of the study.

8 DISCONTINUATION/WITHDRAWAL CRITERIA

8.1 DISCONTINUATION OF STUDY TREATMENT

Not applicable: this is a single-dose study.

8.2 WITHDRAWAL FROM THE STUDY

A participant may withdraw from the study at any time at his/her own request, or may be withdrawn at any time at the discretion of the investigator for safety, behavioral, compliance, or administrative reasons.

If the participant withdraws consent for disclosure of future information, the sponsor may retain and continue to use any data collected before such a withdrawal of consent.

If a participant withdraws from the study, he/she may request destruction of any samples taken and not tested, and the investigator must document this in the site study records.

See SoA for data to be collected at the time of study discontinuation and follow-up and for any further evaluations that need to be completed.

All study withdrawals should be recorded by the Investigator in the appropriate screens of the e-CRF and in the participant's medical records. In the medical record, at least the date of the withdrawal and the reason should be documented.

Withdrawal of consent for follow-up visits should be distinguished from withdrawal of consent for non-patient contact follow-up, eg, medical record checks. The site should document any case of withdrawal of consent.

Participants who have withdrawn from the study cannot be rerandomized (treated) in the study. Their inclusion and treatment numbers must not be reused.

8.3 LOST TO FOLLOW UP

A participant will be considered lost to follow-up if he or she repeatedly fails to return for scheduled visits and is unable to be contacted by the study site.

The following actions must be taken if a participant fails to return to the clinic for a required study visit:

- The site must attempt to contact the participant and reschedule the missed visit as soon as possible and counsel the participant on the importance of maintaining the assigned visit schedule and ascertain whether or not the participant wishes to and/or should continue in the study.

- Before a participant is deemed lost to follow up, the investigator or designee must make every effort to regain contact with the participant (where possible, 3 telephone calls and, if necessary, a certified letter to the participant's last known mailing address or local equivalent methods). These contact attempts should be documented in the participant's medical record.
- Should the participant continue to be unreachable, he/she will be considered to have withdrawn from the study with a primary reason of lost to follow-up.

9 STUDY ASSESSMENTS AND PROCEDURES

Protocol waivers or exemptions are not allowed.

Immediate safety concerns should be discussed with the sponsor immediately upon occurrence or awareness to determine if the participant should continue or discontinue study treatment.

Adherence to the study design requirements, including those specified in the SoA, is essential and required for study conduct.

All screening evaluations must be completed and reviewed to confirm that potential participants meet all eligibility criteria. The investigator will maintain a screening log to record details of all participants screened and to confirm eligibility or record reasons for screening failure, as applicable.

Procedures conducted as part of the participant's routine clinical management (eg, blood count) and obtained before signing of the ICF may be utilized for screening or baseline purposes provided the procedures met the protocol-specified criteria and were performed within the time frame defined in the SoA.

The maximum amount of blood collected from each participant over the duration of the study, including any extra assessments that may be required, will not exceed 110 mL. Repeat or unscheduled samples may be taken for safety reasons or for technical issues with the samples.

9.1 EFFICACY ASSESSMENTS

9.1.1 MEASUREMENT OF PARASITEMIA

Blood sampling for parasitology can be done usually by means of finger prick except when the timing for parasitology assessments coincide with time for clinical laboratory tests, in which case blood films can be done using the venous blood collected for clinical laboratory analyses. Parasite density, for asexual parasite and for gametocyte counts, expressed as the number of parasites per microliter of blood, will be recorded separately throughout the study.

Two qualified microscopists should independently read the stained thick and thin films. Parasite densities will be calculated by averaging the two counts. Blood smears with non-concordant results (differences between the two microscopists in species diagnosis, or differences in parasite density by >50%) will be re-examined by a third, independent microscopist: if discordance relates to parasite density, it will be calculated by averaging the two most concordant counts; if discordance relates to species diagnosis, it will be determined by the third microscopist.

Blood samples or blood spots will be collected for qPCR genotyping analysis and for RT-qPCR gametocyte detection (see Section 9.1.1.4 and Section 9.1.1.5).

For full details of slide preparation, determination of parasitemia and quality control, refer to the study operational manual.

9.1.1.1 Screening/Pre-dose Blood Films

Three slides (two thick films and one thin film) collected at the same timepoint should be prepared using samples taken within 4 hours of dosing. Local thick and thin blood films performed at the site, before Informed Consent signature, according to local standard procedures and within 4 hours prior to dosing, can be used for screening/pre-dose parasitemia assessment, provided that a standard procedure is in place at site and blood films staining is performed according to Study Parasitology Procedures Manual.

The parasite count from the first thick film will be used to calculate the screening parasitemia value.

The second thick film slide will be used to calculate a more accurate pre-dose parasitemia count (asexual).

The thin film slide is used for parasite speciation. Only patients with *P. falciparum* mono-infection should be recruited in the study.

9.1.1.2 Post-dose Blood Films

Three slides (two thick films and one thin film) will be prepared at each planned time point (see SoA for detailed time schedule). The first thick film slide will be used to determine parasite counts. The second thick film slide should be kept as contingency. The thin film slide is used specifically for parasite speciation which should be recorded at each time point.

Additional unscheduled films may be prepared to confirm parasite clearance as described below.

9.1.1.3 Definition of Parasite Clearance (by Microscopy)

A blood film will be considered 'negative' when the examination of 1000 white blood cells reveals no asexual parasites (see the study operational manual for more detail). Parasite clearance time is defined as the time of the first negative film, to be confirmed with a second negative film prepared within 6 to 12 hours of the first one. Parasite clearance will be concluded following confirmation of the second negative film.

If parasites have not cleared by 72 hours after IMP administration and criteria for rescue medication are not met at 72h, blood films should continue to be taken according to site standard practice (or at minimum every 8 hours) until parasite clearance is shown or until criteria for rescue medication are met (see Section 7.7.1).

9.1.1.4 Quantitative PCR, parasite genotyping analysis

Blood samples to measure parasitemia by qPCR will be taken according to SoA; qPCR analysis will be performed by the testing laboratory at all scheduled time points.

Blood samplings for genotyping will be collected according to SoA and at the time-points when recrudescence or re-infection after initial parasite clearance was shown on the blood slide. Parasite genotyping analysis will be performed by the testing laboratory on previously collected blood spot sample only in case of a positive blood film after initial parasite clearance: one pre-dose sample and one sample at 18 or 24 hours post dosing. A further sample will be analyzed at the time point at which recrudescence/re-infection occurs.

9.1.1.5 Gametocyte Detection (RT-qPCR)

Blood samples for RT-qPCR gametocyte detection will be taken according to SoA. RT-qPCR analysis will be performed by the testing laboratory at all scheduled time points to detect submicroscopic gametocytemia and characterize gametocyte maturation stage using the expression of *Pfs25* in mRNA.

9.2 ADVERSE EVENTS

Adverse event of special interest

An AESI) is an AE (serious or nonserious) of scientific and medical concern specific to the Sponsor's product or program, for which ongoing monitoring and immediate notification by the Investigator to the Sponsor is required. Such events may require further investigation in order to characterize and understand them. Adverse events of special interest may be added, modified or removed during a study by protocol amendment.

- **Pregnancy** of a female subject entered in a study as well as pregnancy occurring in a female partner of a male subject entered in a study with IMP/NIMP;
  - Pregnancy occurring in a female patient entered in the clinical trial or in a female partner of a male patient entered in the clinical trial. It will be qualified as an SAE only if it fulfills one of the seriousness criteria (see Appendix 4).
  - Follow-up of the pregnancy in a female participant or in a female partner of a male participant is mandatory until the outcome has been determined (see Appendix 5)
- **Symptomatic overdose** (serious or nonserious) with IMP/noninvestigational medicinal product (NIMP)
  - An overdose (accidental or intentional) with the IMP/NIMP is an event suspected by the Investigator or spontaneously notified by the patient (not based on systematic pills count) and defined as at least twice the intended dose.

  Of note, asymptomatic overdose has to be reported as a standard AE.
- **Increase in alanine transaminase (ALT)**): ALT ≥3 ULN (if baseline ALT <ULN) or, ALT ≥2 times the baseline value (if baseline ALT ≥ULN) will be reported as AESI.

Cases of ALT >3 ULN will be monitored according to the procedures indicated in Appendix 7.

Patients with abnormal ALT values at Day 28 will be followed up until ALT returns to normal values. A PK sample will be made at time of event for OZ439 and FQ (& SSR97213) concentration measurement.

An ALT increase ≥3ULN is reportable as an SAE in any of the following situation:

- Possible Hy's law: ALT or AST >3x ULN and bilirubin >2x ULN (>35% direct bilirubin) in the absence of a serum alkaline phosphatase level >2x ULN. [If fractionation is unavailable, record presence of detectable urinary bilirubin on dipstick indicating direct bilirubin elevations and suggesting liver injury]) or
- when ALT >10 x ULN,
- when associated with jaundice or,
- when associated with coagulation disorder (prothrombin time <50%) or,
- in presence of signs of hepatic encephalopathy.

- Other project specific AESI:
    - **QTcF ≥500 ms or QTcF prolongation > 60 ms** from baseline

In the event of QTcF interval ≥500 ms (automatic measurement) or a QTcF prolongation >60 ms (automatic measurement) from baseline, the patient should be placed under supervision in a specialized setting.

Subsequent ECG monitoring of the patient should then be performed on a regular and clinically responsible basis until the QTc interval returns to their baseline QTcF value.

In case of QTcF ≥500 ms (automatic measurement) or QTcF prolongation >60 ms from baseline, patient will have blood drawn for measurement of OZ439 and FQ (& SSR97213) concentration.

The Investigator will report an AESI only if QTcF ≥500 ms or QTcF prolongation >60 ms from baseline is confirmed by central reading.

The definitions of an AE or SAE can be found in Appendix 4.

AE will be reported by the participant (or, when appropriate, by a caregiver, surrogate, or the participant's legally authorized representative).

The investigator and any designees are responsible for detecting, documenting, and recording events that meet the definition of an AE or SAE and remain responsible for following up AEs that are serious, considered related to the study treatment or study procedures, or that caused the participant to discontinue the study (see Section 8).

9.2.1 Time period and frequency for collecting AE and SAE information

All SAEs will be collected from the signing of the ICF until Day 28 at the time points specified in the SoA (Section 2).

All AE will be collected from the signing of the ICF until Day 28 at the time points specified in the SoA (Section 2).

All SAEs and AESIs will be recorded and reported to the sponsor or designee within 24 hours, as indicated in Appendix 4. The investigator will submit any updated SAE data to the sponsor within 24 hours of it being available.

Investigators are not obligated to actively seek AE or SAE in former study participants. However, if the investigator learns of any SAE, including a death, at any time after a participant has been discharged from the study, and he/she considers the event to be reasonably related to the study treatment or study participation, the investigator must promptly notify the sponsor.

The method of recording, evaluating, and assessing causality of AE and SAE and the procedures for completing and transmitting SAE reports are provided in Appendix 4.

9.2.2 Method of detecting AEs and SAEs

Care will be taken not to introduce bias when detecting AEs and/or SAEs. Open-ended and non-leading verbal questioning of the participant is the preferred method to inquire about AE occurrences.

9.2.3 Follow-up of AEs and SAEs

After the initial AE/SAE report, the investigator is required to proactively follow each participant at subsequent visits/contacts. All SAEs, and non-serious AEs of special interest (as defined in Section 9.2), will be followed until resolution, stabilization, the event is otherwise explained, or the participant is lost to follow-up (as defined in Section 8.3). Further information on follow-up procedures is given in Appendix 4.

9.2.4 Regulatory reporting requirements for SAEs

- Prompt notification by the investigator to the sponsor of a SAE is essential so that legal obligations and ethical responsibilities towards the safety of participants and the safety of a study treatment under clinical investigation are met.
- The sponsor has a legal responsibility to notify both the local regulatory authority and other regulatory agencies about the safety of a study treatment under clinical investigation. The sponsor will comply with country-specific regulatory requirements relating to safety reporting to the regulatory authority, Institutional Review Boards (IRB)/Independent Ethics Committees (IEC), and investigators.

- Investigator safety reports must be prepared for suspected unexpected serious adverse reactions (SUSAR) according to local regulatory requirements and sponsor policy and forwarded to investigators as necessary.
- An investigator who receives an investigator safety report describing a SAE or other specific safety information (eg, summary or listing of SAEs) from the sponsor will review and then file it along with the IB and will notify the IRB/IEC, if appropriate according to local requirements.

9.2.5 Cardiovascular and death events

Cardiovascular and death events will be managed as generally described in Section 9.2.

9.2.6 Disease-related events and/or disease-related outcomes not qualifying as AEs or SAEs

Symptoms related to uncomplicated malaria are not to be considered as AEs or SAEs, except if they meet seriousness criteria. Severe malaria is to be considered as SAE.

9.2.7 Pregnancy

- If a pregnancy is reported, the investigator should inform the sponsor within 24 hours of learning of the pregnancy and should follow the procedures outlined in Appendix 5.
- Abnormal pregnancy outcomes (eg, spontaneous abortion, fetal death, stillbirth, congenital anomalies, ectopic pregnancy) are considered SAEs.

9.2.8 Guidelines for reporting product complaints

Any defect in the IMP/NIMP must be reported as soon as possible by the Investigator to the monitoring team that will complete a product complaint form within required timelines.

Appropriate information (eg, samples, labels or documents like pictures or photocopies) related to product identification and to the potential deficiencies may need to be gathered. The Investigator will assess whether or not the quality issue has to be reported together with an AE or SAE.

9.3 TREATMENT OF OVERDOSE

The sponsor does not recommend specific treatment for an overdose (3, 16). The investigator will treat the symptoms of the overdose by current standard of care.

In the event of an overdose, the investigator or delegate should:

1. Contact the Medical Monitor immediately.
2. Closely monitor the participant for any AE/SAE and laboratory abnormalities until IMP can no longer be detected systemically (at least 5 months).

3. Obtain a plasma sample for PK analysis within 1 day from the date of the last dose of study treatment if requested by the Medical Monitor (determined on a case-by-case basis).
4. Document the quantity of the excess dose as well as the duration of the overdose in the e-CRF.

9.4 SAFETY ASSESSMENTS

Planned time points for all safety assessments are provided in the SoA.

9.4.1 Physical examinations

- Physical examination will include general appearance, head and eyes, ears, nose and throat, chest and lungs, cardiovascular, abdomen, neurological, lymphatic and musculoskeletal and any additional body system considered of relevance by the Investigator. Height and weight will also be measured and recorded.
- A full assessment of uncomplicated *P. falciparum* malaria signs and symptoms will be made alongside the physical examination and will include fever, dizziness, headache, nausea, anorexia, vomiting, diarrhea, itching, urticaria, skin rash, abdominal pain, joint pain, muscle pain, palpitations, sleep problems, confusion, hearing problems, vision problems, and fatigue.
- Investigators should pay special attention to clinical signs related to previous serious illnesses.
- Any new finding or worsening of previous finding should be reported as a new AE.

9.4.2 Vital signs

- Axillary temperature, pulse rate, and blood pressure will be assessed.
- Axillary temperature should be recorded in °C and to an accuracy of one decimal place. If the axillary method is not possible, an alternative route (tympanic, oral or rectal) may be used. The alternative route shall be recorded in the e-CRF. Within an individual patient the same method of temperature measurement (axillary, tympanic, oral or rectal) should be used throughout the entire study period.
- Blood pressure and pulse measurements will be assessed with a completely automated device. Manual techniques will be used only if an automated device is not available.
- Blood pressure and pulse measurements should be preceded by at least 5 minutes of rest in supine position for the participant in a quiet setting without distractions (eg, television, cell phones).
- Vital signs (to be taken before blood collection) will consist of 1 pulse and 3 blood pressure measurements (3 consecutive blood pressure readings will be recorded at intervals of at least 1 minute). The average of the 3 blood pressure readings will be recorded on the e-CRF.

9.4.3 Electrocardiograms

- Heart rate, QRS duration, PR interval, QT interval, ST deviation and T-wave morphology and also U-wave presence or absence will be determined using centralized readings of all ECGs.
- The screening visit ECG will be read by the Investigator and the automatic QTcF calculation will serve as the reference for exclusion criterion E 18.
- All ECG recordings (triplicate) will be centrally read by independent experts.
- Refer to the study operational manual for more details.

9.4.4 Clinical safety laboratory assessments

- See Appendix 2 for the list of clinical laboratory tests to be performed and to the SoA for the timing and frequency.
- The investigator must review the laboratory report, document this review, and record any clinically relevant changes occurring during the study in the AE section of the e-CRF. The laboratory reports must be filed with the source documents. Clinically significant abnormal laboratory findings are those which are not associated with the underlying disease, unless judged by the investigator to be more severe than expected for the participant's condition.
- All laboratory tests with values considered clinically significantly abnormal during participation in the study should be repeated until the values return to normal or baseline or are no longer considered clinically significant by the investigator or medical monitor.
  - If such values do not return to normal/baseline within a period of time judged reasonable by the investigator, the etiology should be identified and the sponsor notified.
  - All protocol-required laboratory assessments, as defined in Appendix 2, must be conducted in accordance with the local laboratory manual and the SoA.
  - If laboratory values from non-protocol specified laboratory assessments performed at the institution’s local laboratory require a change in participant management or are considered clinically significant by the investigator (eg, SAE or AE or dose modification), then the results must be recorded in the CRF.

9.5 PHARMACOKINETICS

Whole blood samples will be collected for measurement of plasma concentrations of OZ439 and blood concentration (Dry Blood Spot [DBS]) of FQ and SSR97213 as specified in the SoA. The list of PK samples is provided in Table 3. Instructions for the collection and handling of biological samples will be provided by the sponsor. The actual date and time (24-hour clock time) of each sample will be recorded.

Table 3 – Numbering of PK samples

Timepoint	FQ and SSR97213 in blood	OZ439 in plasma
Predose	DBS00	P00
1 h	DBS01	P01
2 h	-	P02
4 h	DBS02	P03
6 h	DBS03	P04
8 h	DBS04	-
12 h	DBS05	P05
24 h	DBS06	P06
48 h	-	P07
72 h	DBS07	P08
120 h	-	P09
168 h	DBS08	P10
Day 14±1	DBS09	P11
Day 28±2	DBS10	-
Total number of samples	11	12

A summary of handling procedures is provided in Table 4 and Table 5. Blood sample of at least 1.2 mL (*) will be used to evaluate the PK of FQ and SSR97213 and the PK of OZ439.

Table 4 – Summary of handling procedure of PK blood samples

	FQ and SSR97213 in blood
Anticoagulant	K3-ethylenediaminetetraacetic acid (EDTA)
Handling procedure	see study operational manual specifications
Blood volume	At least 1.2 mL (*)
Blood aliquot split	2
Blood storage conditions	Dried Blood Spot (DBS)
Blood shipment conditions	Room temperature

Table 5 – Summary of handling procedure of PK plasma samples

	OZ439 in plasma
Anticoagulant	K3-EDTA
Handling procedure	see study operational manual specifications
Blood volume	At least 1.2 mL (*)
Blood aliquot split	2
Blood storage conditions	Frozen at -20°C
Blood shipment conditions	Dry ice

Each sample for each analyte will be divided into 2 aliquots:

- Aliquot #1 samples will be sent to the reference laboratory
- Aliquot #2 samples will be stored at the investigator's site until further instructions

Plasma and DBS remaining after determination of the above concentrations may be used for possible exploratory analysis of drug/metabolites.

A summary of bioanalytical methods is provided in Table 6.

Table 6 – Summary of PK bioanalytical methods

	FQ and SSR97213 in blood	OZ439 in plasma
Analyte	FQ (SSR97193) and SSR97213	OZ439
Matrix	Dried Blood Spot	Plasma
Analytical technique	Liquid chromatography tandem mass spectroscopy (LC-MS/MS)	LC-MS/MS
Lower limit of quantification	5 ng/mL	1 ng/mL
Assay volume	Whole spot of 10 µL	50 µL
Site of bioanalysis	Covance, Salt Lake City, UT, US	Swiss BioQuant AG
Method reference	TSLS15-350 (Sanofi number: DOH1364)	SBQ-12026

The PK parameters derived from the concentrations are described in Section 4.

Genetic analyses will not be performed on these samples.

Residual or leftover plasma or blood samples may be used for additional research purposes on malaria, after the patient will have consented to it.

9.6 PHARMACODYNAMICS

Pharmacodynamic parameters are not evaluated in this study.

9.7 GENETICS

Human genetics are not evaluated in this study.

9.8 BIOMARKERS

Biomarkers are not evaluated in this study.

9.9 MEDICAL RESOURCE UTILIZATION AND HEALTH ECONOMICS

Health Economics/Medical Resource Utilization and Health Economics parameters are not evaluated in this study.

10 STATISTICAL CONSIDERATIONS

10.1 SAMPLE SIZE DETERMINATION

The strategy for selecting fixed FQ dose, baseline parasitemia, and sample size was composed of two parts:

1. Estimated efficacy (Day 28 PCR corrected ACPR) of FQ 200, 400, 600 and 900 mg administrated in presence of OZ439 TPGS for different levels of baseline parasitemia
2. Estimated power for various sample size of an OZ439 exposure-effect analysis, considering the fixed FQ dose, the 4 selected OZ439 dose levels (0, 300, 600, 1000 mg) and the selected baseline parasitemia

Details of the method are provided in Appendix 8.

Results estimated that 30 evaluable participants per treatment arm will yield a power around 90% to detect an OZ439 concentration effect relation (in a population of patient with a baseline parasitemia greater than 3000 parasites/µL, for 4 arms FQ 400 mg + OZ439 at 0, 300, 600, and 1000 mg).

Around 15 % of early dropout is anticipated, consequently approximately 35 participants are to be included in each arm (to get 30 evaluable participants/arm) resulting in approximately 140 participants to be enrolled in the whole study.

10.2 POPULATIONS FOR ANALYSES

For purposes of analysis, the following populations are defined (Table 7):

Table 7 - Populations for analyses

Population	Description
Screened participants	All participants who sign the ICF and met the inclusion criteria
Randomized participants (intent to treat [ITT])	All participants with a treatment kit number allocated and recorded in IRT database at Visit 2 (Day 0), and regardless of whether the treatment kit was used or not
Evaluable (modified ITT [mITT])	All randomized participants with parasitologically confirmed malaria at baseline, who received at least the single administration of OZ439/FQ 400 mg and who have parasitemia data post-randomization. Participants will be analyzed according to the treatment arm allocated by randomization
Safety	All randomized participants who take at least 1 dose or part of a dose of the single administration OZ439 & FQ 400 mg. Participants will be analyzed according to the treatment they actually received
PK	All participants included in the Safety population with at least one evaluable blood sample for PK post IMP administration and with adequate documentation of date of dosing and date of sampling
PK/PD	All participants included in both the mITT population and the PK population

10.3 STATISTICAL ANALYSES

The statistical analysis plan (SAP) will be developed and finalized before database lock and will describe the participant populations to be included in the analyses, and procedures for accounting for missing, unused, and spurious data. This section is a summary of the planned statistical analyses of the primary and secondary endpoints.

10.3.1 Efficacy analyses

All efficacy analyses will be performed on the mITT Population. The efficacy analyses are summarized in Table 8.

Table 8 - Efficacy analyses

Endpoint	Statistical Analysis Methods
For primary objective:	
• Day 28 PCR-corrected ACPR	• The exposure-response association of single-dose OZ439/FQ for PCR-corrected ACPR at Day 28 will demonstrate how the probability of a response in PCR-corrected ACPR at Day 28 increases with exposure (AUC) to OZ439/FQ. Logistic regression with p: probability of PCR-corrected ACPR at Day 28 and OZ439 AUC and FQ $AUC_{0\text{-}Day28}$ (SSR97213 $AUC_{0\text{-}Day28}$ will be considered also) and baseline parasitemia as covariates will be fitted.<br>• Results of logistic regressions of PCR-corrected ACPR on exposure will be provided in a summary table including odds ratio estimates, the corresponding 95% Wald CIs and p-value.<br>• Frequency table for Day 28 PCR-corrected ACPR, i.e. proportion, percentage and exact binomial 95% CIs (two-sided by using Clopper-Pearson method for calculating binomial CIs) by treatment arm, will also be provided.
For secondary objectives:	
• Day 28 PCR-corrected ACPR	• The dose-response exposure-response association of single-dose OZ439/FQ for PCR-corrected ACPR at Day 28 will demonstrate how the probability of a response in PCR-corrected ACPR at Day 28 increases with increasing dose of OZ439/FQ thanks to a logistic regression. Results of logistic regressions of PCR-corrected ACPR on dose will be provided in a summary table including odds ratio estimates, the corresponding 95% Wald CIs, and p-value.
• Parasitemia at baseline then every 6 h during the first 36 h then at 48 h and every 24 h until Day 7	• Descriptive statistics (number of observations [n], mean, geometric mean, standard deviation [SD], median, first quartile [Q1], third quartile [Q3], minimum, and maximum) for parasitemia data (number of parasites/µL) (raw data and absolute change from baseline) will be displayed at each post-baseline assessment. Time profile plots (Mean ± standard error of the mean) of parasitemia will be provided for each treatment arm, one curve per arm.
• Day 28 crude ACPR	• Same analysis using Clopper-Pearson method as for PCR-corrected ACPR will be done on crude ACPR at Day 28. The exposure-response (respectively dose-response) association of single-dose OZ439/FQ for crude ACPR will also be evaluated.<br>A generalized MCP-Mod approach will be used with PCR-corrected and crude ACPR at Day 28. Both linear and log model will be fitted. Then the dose response signal will be tested via contrasts based on dose-response shape. Selection of the model needs investigations which will be detailed in the SAP.

• Observed PRR at 24 h, 48 h, and 72 h	• Descriptive statistics (n, mean, geometric mean, standard deviation, median, Q1, Q3, minimum, and maximum) by treatment arm and time of measurement.
• Parasite clearance rate	• Parasite clearance estimator established by WWARN (1) will be used to identify the lag phase, clean the data for outliers, and determine the best model (linear, quadratic, cuboidal) to fit the log-transformed parasite data and afterward calculate the following estimate: the parasite clearance rate. Descriptive statistics (median, first Q1, Q3, minimum, and maximum) will be provided for each treatment arm.
• Time<br>- to parasite clearance<br>- to 99% parasite reduction<br>- to recrudescence or re-infection<br>- elapsed below LOQ of parasitemia	• Kaplan-Meier estimators.<br>• For each variable, an overall overview including number of events (frequency and %), 25%, median and 75% quantile with 95% CI (expressed in weeks), cumulative incidence of events with 95% CI will be provided in a summary table for all participants.<br>• Survival curves will be plotted by representing each treatment arm for all participants.<br>• Cox model will be used for time to event endpoints with OZ439 doses and parasitemia at baseline as covariates, potential interaction will be explored.
Exploratory	Will be described in the statistical analysis plan finalized before database lock

10.3.1.1 Definition of ACPR, recrudescence, and re-infection

ACPR is defined based on WHO recommendations (2) as follows:

ACPR

- absence of parasitemia (blood films) on Day 28, irrespective of axillary temperature, without previously meeting any of the criteria of early treatment failure or late clinical failure or late parasitological failure (see definitions in Section 7.7.1) or having received a rescue treatment for malaria in the conditions defined in Section 7.7.1.

Recrudescence

- appearance of asexual parasites after clearance of initial infection with a genotype identical to that of parasites present at baseline.

Re-infection

- appearance of asexual parasites after clearance of initial infection with a genotype that differs from that of parasites present at baseline. Confirmed new infection will not be regarded as treatment failure or recrudescence.

Crude ACPR

- Crude ACPR applies to both recrudescence of the parasite clone responsible for baseline infection and re-infection by a different clone.

PCR-corrected ACPR

- the PCR-corrected ACPR applies only to recrudescence (re-emergence of the original clone of parasite that is present at baseline). Recrudescence is distinguished from re-infection by genotyping the parasite clone.

10.3.2 Safety analyses

All safety analyses will be performed on the Safety Population (see Table 7). The safety analyses are summarized in Table 9.

Table 9 - Safety analyses

Endpoint	Statistical Analysis Methods
For secondary objectives	The summary of safety results will be presented by treatment arm thanks to descriptive statistics (summary tables, graphics). No formal inferential testing will be performed.
• AEs	• Frequency tables of TEAEs and SAEs by treatment arm during the on-treatment phase<br>• Summary tables of participant count (%) for each AESI by treatment arm<br>• Summary tables of deaths<br>• incidence of liver-related AEs summarized by treatment arm
• Clinical laboratory tests	• Counts of participants with potentially clinically significant abnormalities (PCSAs) at any time during the on-treatment phase will be summarized by treatment arm in summary tables showing shifts from normal and abnormal baselines to postbaseline abnormalities.
• LFT	• raw data and changes from baseline summarized in descriptive statistics, by treatment arm and visit for all participants<br>• time profile plots (mean+/- SD) on raw data and change from baseline<br>• proportion of participants with PCSA values at any post baseline visit by baseline status will be displayed by treatment arm<br>• graph of distribution (e-Dish plot) of peak values of ALT versus peak values of total bilirubin<br>• normalization (to ≤1 x ULN or return to baseline) of elevated liver function tests summarized by categories of elevation
• Vital signs	• raw parameter value and change from baseline<br>• Counts of participants with post-baseline PCSAs in summary tables by treatment arm
• ECG	• Heart rate, PR-, QRS-, and QT-intervals, corrected QTc (Fridericia) will be analyzed as raw parameter value and change from baseline, as well as percent change from baseline for selected parameters<br>• Counts of participants with post-baseline PCSAs in summary tables by treatment arm.<br>• A summary of ECG morphological assessments (for all high level types of comments) by treatment arm<br>• Summary plots (mean+/-SD) on raw data and changes from baseline, as well as percent change from baseline for selected parameters
• Physical examination	• Summary tables of participant count (%) for each physical examination (general appearance, head and eyes, ears, nose and throat, chest and lungs, cardiovascular,

	abdomen, neurological, lymphatic and musculoskeletal and any additional body system considered of relevance by the Investigator) by treatment arm.
• Clinical signs and symptoms related to uncomplicated *P. falciparum* malaria	• Summary tables of participant count (%) for each of the clinical signs and symptoms (Fever, Dizziness, Headache, Nausea, Anorexia, Vomiting, Diarrhea, Itching, Urticaria, Skin Rash, Abdominal Pain, Joint Pain, Muscle Pain, Palpitations, Sleep Problems, Confusion, Hearing Problems, Vision Problems, and Fatigue) by primary system organ class, high level term and treatment arm.

10.3.2.1 Observation period

For all safety data (see Section 9.2 and Section 9.4 for details), the observation period will be divided into three phases:

- The pre-treatment phase defined as the time between the participant gives informed consent and the start time of first IMP administration (excluded)
- The on-treatment phase defined as the start time of first dose of IMP administration (included) up to Day 28 visit (included)
- The post-treatment phase will be defined as the time after the Day 28 visit (excluded)

10.3.2.2 Baseline

The baseline value is defined as the last available value before first IMP at Visit 1 (screening) for laboratory and vital signs and at Visit 2 (Day 0 before IMP administration) for ECG parameters.

10.3.2.3 Adverse events

AEs will be coded according to the Medical Dictionary for Regulatory Activities (MedDRA, last version available before database lock).

They will be classified into predefined standard categories according to chronological criteria:

- Pre-treatment AEs are defined as AEs that occurred, worsened (according to Investigator opinion) or became serious during the pre-treatment phase
- TEAEs are defined as AEs that occurred or worsened or became serious during the on-treatment phase
- Post-treatment AEs are defined as AEs that occurred worsened or became serious during the post-treatment phase.

10.3.2.4 Potentially clinically significant abnormalities

The following definitions will be applied to laboratory parameters, vital signs, and ECG:

- The PCSA values are defined as abnormal values considered medically important by the Sponsor according to predefined criteria/thresholds based on literature review and defined by the Sponsor for clinical laboratory tests, vital signs and ECG

- PCSA criteria will determine which participants had at least 1 PCSA during the on-treatment phase, taking into account all evaluations performed during the on-treatment phase, including unscheduled or repeated evaluations. The number of all such participants will be the numerator for the PCSA percentage.

10.3.2.5 Drug-induced liver injury and QT prolongation

To prevent missing any event related to the AESI defined for the study (see Section 9.2), the selection of preferred terms will be based on standardized MedDRA queries (SMQs):

- Hepatic disorder (code 20000005, level 1, active) for potential DILI events
- Torsade de pointes/QT prolongation (code 20000001, level 1, active) for potential cardiac events

Time to liver-related treatment discontinuation and time to liver death may also be provided based on hepatic disorder SMQ.

10.3.3 PK analyses

All PK analyses will be performed on the PK Population (see Table 7). The PK analyses are summarized in Table 10.

Table 10 - PK analyses

Endpoint	Statistical Analysis Methods
For secondary objectives	
• Concentrations	• Descriptive statistics by treatment arm and by time point
• PK parameters	• Descriptive statistics (such as arithmetic mean, geometric mean, median, SD, SEM, coefficient of variation, minimum, and maximum) for each treatment arm

10.3.3.1 Pharmacokinetic parameters

Pharmacokinetic parameters are listed in Section 4.

The PK parameters will be estimated through Bayesian analysis using previously developed population PK models.

The Bayesian PK analyses for OZ439 and for Ferroquine and its metabolite will be reported separately from the main clinical report in 2 different reports.

10.3.4 Other analyses

Exploratory analyses (such as PK/QTc analyses) will be described in the SAP finalized before database lock. The PK/QTc analysis will be presented in the clinical report; the parasitemia evaluated by qPCR (pharmacodynamics) and the exploratory analysis between parasitemia

(measured by qPCR) and concentration of OZ439/FQ analyses will be presented separately from the main clinical study report.

10.3.5 Interim analyses

No interim analysis is planned (16).

11 REFERENCES

1. Flegg JA, Guerin PJ, White NJ, Stepniewska K. Standardizing the measurement of parasite clearance in falciparum malaria: the parasite clearance estimator. Malar J. 2011 Nov 10;10:339. doi: 10.1186/1475-2875-10-339.

2. Methods for surveillance of antimalarial drug efficacy, WHO, 2009, ISBN 978 92 4 159753 1.

3. Medicines for Malaria Venture. Investigator's Brochure OZ439 (artefenomel). Version 11.0, 20 July 2017.

4. U.S. Department of Health and Human Services Food and Drug Administration (FDA) - Center for Drug Evaluation and Research (CDER). Guidance for Industry Co-development of Two or More New Investigational Drugs for Use in Combination. Clinical Medical. June 2013.

5. World Health Organization. World Malaria Report 2016. 2016. ISBN: 978 92 4 151171 1.

6. World Health Organization. Guidelines for the treatment of Malaria. Third edition. 2015. ISBN: 978 92 4 154912 7.

7. World Health Organization. Management of severe malaria: a practical handbook. Third edition. 2012. ISBN: 978924154852 6.

8. Tun KM, Imwong M, Lwin KM, Win AA, Hlaing TM, Hlaing T, et al, Spread of artemisinin-resistant Plasmodium falciparum in Myanmar: a cross-sectional survey of the K13 molecular marker. Lancet Infect Dis. 2015 Apr; 15(4):415-21.

9. Denis MB, Tsuyuoka R, Poravuth Y, Narann TS, Seila S, Lim C, et al. Surveillance of the efficacy of artesunate and mefloquine combination for the treatment of uncomplicated falciparum malaria in Cambodia. Trop Med Int Health. 2006 Sep;11(9):1360-6.

10. Rogers WO, Sem R, Tero T, Chim P, Lim P, Muth S, et al. Failure of artesunate-mefloquine combination therapy for uncomplicated Plasmodium falciparum malaria in southern Cambodia. Malar J. 2009 Jan; 8:10.

11. Noedl H, Se Y, Schaecher K, Smith BL, Socheat D, Fukuda MM. Evidence of artemisinin-resistant malaria in western Cambodia. N Engl J Med. 2008 Dec 11;359(24):2619-20.

12. Dondorp AM, Nosten F, Yi P, Das D, Phyo AP, Tarning J, et al. Artemisinin Resistance in Plasmodium falciparum Malaria. N Engl J Med. 2009 Jul 30;361(5):455-67.

13. World Health Organization. Emergence and spread of artemisinin resistance calls for intensified efforts to withdraw oral artemisinin based monotherapy from the market. Global Malaria Programme. May 2014.

14. Leang R, Barette A, Bouth DM, Menard D, Abdur R, Duong S, et al. Efficacy of dihydro-artemisnin-piperaquine for treatment of uncomplicated Plasmodium falciparum and Plasmodium vivax in Cambodia, 2008 to 2010. Antimicrob Agents Chemother. 2013 Feb;57(2);818-26.

15. Saunders DL, Vanachayangkul P, Lon C. Dihydroartemisinin–Piperaquine Failure in Cambodia. N Engl J Med. 2014 Jul 31;371(5):484-5.

16. Sanofi-Aventis. Investigator's Brochure SSR97193-Ferroquine. Version 11, 30 August 2017.

17. Macintyre F, Laurijssens B, Adamy M, Adoke Y, Bassat Q, Biguenet S, et al. Phase IIb study of artefenomel (OZ439) and piperaquine to investigate single dose treatment for uncomplicated P. falciparum malaria. ASTMH 2016, poster 1535.

18. World Food Program and Centers for Diseases Control and Prevention. A manual: measuring and interpreting malnutrition and mortality. Nov 2005. http://www.unhcr.org/45f6abc92.pdf19.Held J, Supan C, Salazar CLO, Tinto H, Bonkian LN, Nahum A, et al. Ferroquine and artesunate in African adults and children with Plasmodium falciparum malaria: a phase 2, multicenter, randomized, double-blind, dose-ranging, non-inferiority study. Lancet Infect Dis. 2015 Dec 3;15(12):1409-19.

12 APPENDICES

Appendix 1 Abbreviations

°C:	degree Celsius
ACPR:	adequate clinical and parasitological response
ACT:	artemisinin-based combined therapy
AE:	adverse event
AESI:	adverse event of special interest
AF:	atrial fibrillation
ALT:	alanine transferase
AST:	aspartate transferase
AUC:	area under the concentration time curve up to infinity
$AUC_{0\text{-}Day28}$:	area under the concentration time curve from T0 to Day 28
C_{168h}:	concentraton at 168 h post dose
CI:	confidence interval
C_{max}:	maximum concentration
CONSORT:	Consolidated Standards of Reporting Trials
CYP:	cytochrome P450
DBS:	Dry Blood Spot
ECG:	electrocardiogram
e-CRF:	electronic case report form
EDTA:	ethylenediaminetetraacetic acid
FDA:	Food and Drug Administration
FDC:	fixed dose combination
FQ:	ferroquine
HAV IgM:	hepatitis A immunoglobulin M
HBsAg:	hepatitis B surface antigen
HCG:	human chorionic gonadotropin
HCV Ab:	hepatitis C virus antibody
HCV RNA:	hepatitis C virus ribonucleic acid
IB:	investigator's brochure
ICF:	informed consent form
IEC:	Independent Ethics Committee
IMP:	investigational medicinal product
IND:	investigational new drug
INN:	international nonproprietary name
IRB:	Institutional Review Board
IRT:	interactive response technology
ITT:	intent to treat
LAR:	legally acceptable representative
LC-MS/MS:	liquid chromatography tandem mass spectroscopy
LFT:	liver function tests
LOQ:	limit of quantification
M&S:	modeling and simulations
MCP-Mod:	multiple comparisons procedures-modeling

MedDRA:	Medical Dictionary for Regulatory Activities
mITT:	modified intent to treat
MMV:	Medicines for Malaria Venture
n:	number of observations
NDA:	new drug administration
NIMP:	noninvestigational medicinal product
P. falciparum:	Plasmodium falciparum
PCR:	polymerase chain reaction
PCSA:	potentially clinically significant abnormalities
P-gp:	P-glycoprotein
PK:	pharmacokinetic(s)
PQP:	piperaquine phosphate
PRR:	parasite reduction rate
Q1:	first quartile
Q3:	third quartile
qPCR:	quantitative polymerase chain reaction
QTcF:	QT interval corrected using Fridericia formula
RT-qPCR:	quantitative reverse transcription polymerase chain reaction
SAE:	serious adverse event
SAP:	statisctical analysis plan
SD:	standard deviation
SMQ:	standardized MedDRA query
SoA:	schedule of activities
SUSAR:	suspected unexpected serious adverse reaction
$t_{1/2}$:	terminal half-life
TEAE:	treatment emergent adverse event
t_{max}:	time to reach the maximal concentration
TPGS:	alpha tocopherol polyethylene glycol 1000 succinate
ULN:	upper limit of normal range
USFDA:	United States Food and Drug Administration
WHO:	World Health Organization
WOCBP:	woman of childbearing potential
WWARN:	worldwide antimalarial resistance network

Appendix 2 Clinical laboratory tests

- The tests detailed in Table 11 will be performed by the local laboratory
- Protocol-specific requirements for inclusion or exclusion of participants are detailed in Section 6.1 and Section 6.2 of the protocol.
- Additional tests may be performed at any time during the study as determined necessary by the investigator or required by local regulations.

Table 11 - Protocol-required safety laboratory assessments

Laboratory assessments	Parameters			
Hematology	Platelet count<br>RBC count<br>Hemoglobin<br>Hematocrit<br>Reticulocytes[a]			WBC Count with Differential:<br>Neutrophils<br>Lymphocytes<br>Monocytes<br>Eosinophils<br>Basophils
Clinical chemistry[b]	Urea	Potassium	Aspartate aminotransferase (AST)	Total bilirubin
	Lactate dehydrogenase[a]	Sodium	Alanine aminotransferase (ALT)	Direct bilirubin if total bilirubin ≥ULN[a]
	Creatinine	Magnesium (screening)	Alkaline phosphatase	Albumin
	Creatinine clearance[c]	Calcium (screening)		Haptoglobin[a]
	Creatine kinase			
	Glucose (non-fasting)			
Routine urinalysis	• Specific gravity<br>• pH, glucose, protein, blood, ketones, bilirubin, leukocyte esterase by dipstick<br>• Microscopic examination (if blood or protein is abnormal)<br>• Pregnancy test (urine β-HCG)[d] for WOCBP			
Other screening tests	• Hepatitis serology: HAV IgM, HBsAg, HCV Ab, and/or HCV RNA (if known)<br>• The results of each test must be entered into the e-CRF.			

NOTES :

a Test not needed for screening but must be available during the study to monitor the participant's health status. The test must be either available locally or in a national reference laboratory with which the site has an agreement for performing the test.

b Details of liver chemistry criteria and required actions and follow-up assessments are given in Section 9.2 and Appendix 7. .

c For participants >18 years old, the creatinine clearance (CrCl) will be calculated from Cockroft and Gault formula: CrCl (mL/min) = 1.23 (for male) or 1.04 (for female) x weight (kg) x (140 - Age) / serum creatinine (µmol/L) with age in years. For participants ≤18 years old, the GFR Bedside Schwartz Formula should be used: eGFR (mL/min/1.73 m²) = 0.413 x height (cm) / serum creatinine (mg/dL)

d Local urine testing will be standard for the protocol unless serum testing is required by local regulation or IRB/IEC.

Investigators must document their review of each laboratory safety report.

Appendix 3 Study governance considerations

Regulatory and ethical considerations

- This study will be conducted in accordance with the protocol and with the following:
  - Consensus ethical principles derived from international guidelines including the Declaration of Helsinki and Council for International Organizations of Medical Sciences (CIOMS) International Ethical Guidelines
  - Applicable ICH Good Clinical Practice (GCP) Guidelines
  - Applicable laws and regulations
- The protocol, protocol amendments, ICF, Investigator Brochure, and other relevant documents (eg, advertisements) must be submitted to an IRB/IEC by the investigator and reviewed and approved by the IRB/IEC before the study is initiated.
- Any amendments to the protocol will require IRB/IEC approval before implementation of changes made to the study design, except for changes necessary to eliminate an immediate hazard to study participants.
- The investigator will be responsible for the following:
  - Providing written summaries of the status of the study to the IRB/IEC annually or more frequently in accordance with the requirements, policies, and procedures established by the IRB/IEC
  - Notifying the IRB/IEC of SAEs or other significant safety findings as required by IRB/IEC procedures
  - Providing oversight of the conduct of the study at the site and adherence to requirements of 21 CFR, ICH guidelines, the IRB/IEC, European regulation 536/2014 for clinical studies (if applicable), and all other applicable local regulations

Financial disclosure

Investigators and sub-investigators will provide the sponsor with sufficient, accurate financial information as requested to allow the sponsor to submit complete and accurate financial certification or disclosure statements to the appropriate regulatory authorities. Investigators are responsible for providing information on financial interests during the course of the study and for 1 year after completion of the study.

Informed consent process

- The investigator or his/her representative will explain the nature of the study to the participant or his/her legally authorized representative and answer all questions regarding the study.
- Participants must be informed that their participation is voluntary. Participants or their legally authorized representative will be required to sign a statement of informed consent that meets the requirements of 21 CFR 50, local regulations, ICH guidelines, Health

Insurance Portability and Accountability Act (HIPAA) requirements, where applicable, and the IRB/IEC or study center.

- The medical record must include a statement that written informed consent was obtained before the participant was enrolled in the study and the date the written consent was obtained. The authorized person obtaining the informed consent must also sign the ICF.
- Participants must be re-consented to the most current version of the ICF(s) during their participation in the study.
- A copy of the ICF(s) must be provided to the participant or the participant's legally authorized representative.
- Participants who are rescreened are required to sign a new ICF.

Data protection

- Participants will be assigned a unique identifier by the sponsor. Any participant records or datasets that are transferred to the sponsor will contain the identifier only; participant names or any information which would make the participant identifiable will not be transferred.
- The participant must be informed that his/her personal study-related data will be used by the sponsor in accordance with local data protection law. The level of disclosure must also be explained to the participant.
- The participant must be informed that his/her medical records may be examined by Clinical Quality Assurance auditors or other authorized personnel appointed by the sponsor, by appropriate IRB/IEC members, and by inspectors from regulatory authorities.

Safety Management Team

A Safety Management Team (SMT), cross-functional team led by the Global Safety Officer, will be set-up to ensure comprehensive characterization of the safety profile of IMP and appropriate risk mitigation.

The SMT may identify and invite ad-hoc members or contributors (external and/or internal) as required providing necessary expertise based on the safety medical concepts/issues to be reviewed.

Publication policy

- The results of this study may be published or presented at scientific meetings. If this is foreseen, the investigator agrees to submit all manuscripts or abstracts to the sponsor before submission. This allows the sponsor to protect proprietary information and to provide comments.
- The sponsor will comply with the requirements for publication of study results. In accordance with standard editorial and ethical practice, the sponsor will generally support publication of multicenter studies only in their entirety and not as individual site data. In this case, a coordinating investigator will be designated by mutual agreement.

- Authorship will be determined by mutual agreement and in line with International Committee of Medical Journal Editors authorship requirements.

Dissemination of clinical study data

Sanofi shares information about clinical trials and results on publically accessible websites, based on company commitments, international and local legal and regulatory requirements, and other clinical trial disclosure commitments established by pharmaceutical industry associations. These websites include clinicaltrials.gov, EU clinicaltrialregister (eu.ctr), and sanofi.com, as well as some national registries.

In addition, results from clinical trials in patients are required to be submitted to peer-reviewed journals following internal company review for accuracy, fair balance and intellectual property. For those journals that request sharing of the analyzable data sets that are reported in the publication, interested researchers are directed to submit their request to clinicalstudydatarequest.com.

Individual participant data and supporting clinical documents are available for request at clinicalstudydatarequest.com. While making information available we continue to protect the privacy of participants in our clinical trials. Details on data sharing criteria and process for requesting access can be found at this web address: clinicalstudydatarequest.com.

Data quality assurance

- All participant data relating to the study will be recorded on printed or e-CRF unless transmitted to the sponsor or designee electronically (eg, laboratory data). The investigator is responsible for verifying that data entries are accurate and correct by physically or electronically signing the e-CRF.
- The investigator must maintain accurate documentation (source data) that supports the information entered in the e-CRF.
- The investigator must permit study-related monitoring, audits, IRB/IEC review, and regulatory agency inspections and provide direct access to source data documents.
- The sponsor or designee is responsible for the data management of this study including quality checking of the data.
- Study monitors will perform ongoing source data verification to confirm that data entered into the e-CRF by authorized site personnel are accurate, complete, and verifiable from source documents; that the safety and rights of participants are being protected; and that the study is being conducted in accordance with the currently approved protocol and any other study agreements, ICH GCP, and all applicable regulatory requirements.
- Records and documents, including signed ICFs, pertaining to the conduct of this study must be retained by the investigator for 15 years after study completion unless local regulations or institutional policies require a longer retention period. No records may be destroyed during the retention period without the written approval of the sponsor. No records may be transferred to another location or party without written notification to the sponsor.

Source documents

- Source documents provide evidence for the existence of the participant and substantiate the integrity of the data collected. Source documents are filed at the investigator’s site.
- Data entered in the e-CRF that are transcribed from source documents must be consistent with the source documents or the discrepancies must be explained. The investigator may need to request previous medical records or transfer records, depending on the study. Also, current medical records must be available.
- Source data is all information in original records and certified copies of original records of clinical findings, observations, or other activities in a clinical study necessary for the reconstruction and evaluation of the study. Source data are contained in source documents (original records or certified copies).

Study and site closure

The sponsor designee reserves the right to close the study site or terminate the study at any time for any reason at the sole discretion of the sponsor. Study sites will be closed upon study completion. A study site is considered closed when all required documents and study supplies have been collected and a study-site closure visit has been performed.

The investigator may initiate study-site closure at any time, provided there is reasonable cause and sufficient notice is given in advance of the intended termination.

Reasons for the early closure of a study site by the sponsor or investigator may include but are not limited to:

- Failure of the investigator to comply with the protocol, the requirements of the IRB/IEC or local health authorities, the sponsor's procedures, or GCP guidelines
- Inadequate recruitment of participants by the investigator
- Discontinuation of further study treatment development

Appendix 4 Adverse events: definitions and procedures for recording, evaluating, follow-up, and reporting

DEFINITION OF AE

AE definition

- An AE is any untoward medical occurrence in a patient or clinical study participant, temporally associated with the use of study treatment, whether or not considered related to the study treatment.
- NOTE: An AE can therefore be any unfavorable and unintended sign (including an abnormal laboratory finding), symptom, or disease (new or exacerbated) temporally associated with the use of study treatment.

Events meeting the AE definition

- Any abnormal laboratory test results (hematology, clinical chemistry, or urinalysis) or other safety assessments (eg, ECG, radiological scans, vital signs measurements), including those that worsen from baseline, considered clinically significant in the medical and scientific judgment of the investigator (ie, not related to progression of underlying disease).
- Exacerbation of a chronic or intermittent pre-existing condition including either an increase in frequency and/or intensity of the condition.
- New conditions detected or diagnosed after study treatment administration even though it may have been present before the start of the study.
- Signs, symptoms, or the clinical sequelae of a suspected drug-drug interaction.
- Signs, symptoms, or the clinical sequelae of a suspected overdose of either study treatment or a concomitant medication.
- "Lack of efficacy" or "failure of expected pharmacological action" per se will not be reported as an AE or SAE. Such instances will be captured in the efficacy assessments. However, the signs, symptoms, and/or clinical sequelae resulting from lack of efficacy will be reported as AE or SAE if they fulfil the definition of an AE or SAE.

Events NOT meeting the AE definition

- Any clinically significant abnormal laboratory findings or other abnormal safety assessments which are associated with the underlying disease, unless judged by the investigator to be more severe than expected for the participant's condition.
- The disease/disorder being studied or expected progression, signs, or symptoms of the disease/disorder being studied, unless more severe than expected for the participant's condition.
- Medical or surgical procedure (eg, endoscopy, appendectomy): the condition that leads to the procedure is the AE.

- Situations in which an untoward medical occurrence did not occur (social and/or convenience admission to a hospital).
- Anticipated day-to-day fluctuations of pre-existing disease(s) or condition(s) present or detected at the start of the study that do not worsen

DEFINITION OF SAE

If an event is not an AE per definition above, then it cannot be an SAE even if serious conditions are met (eg, hospitalization for signs/symptoms of the disease under study, death due to progression of disease).

A SAE is defined as any untoward medical occurrence that, at any dose:

a) **Results in death**

b) **Is life-threatening**

   The term 'life-threatening' in the definition of 'serious' refers to an event in which the participant was at risk of death at the time of the event. It does not refer to an event, which hypothetically might have caused death, if it were more severe.

c) **Requires inpatient hospitalization or prolongation of existing hospitalization**

   In general, hospitalization signifies that the participant has been detained (usually involving at least an overnight stay) at the hospital or emergency ward for observation and/or treatment that would not have been appropriate in the physician’s office or outpatient setting. Complications that occur during hospitalization are AEs. If a complication prolongs hospitalization or fulfills any other serious criteria, the event is serious. When in doubt as to whether “hospitalization” occurred or was necessary, the AE should be considered serious.

   Hospitalization for elective treatment of a pre-existing condition that did not worsen from baseline is not considered an AE.

d) **Results in persistent disability/incapacity**

   - The term disability means a substantial disruption of a person’s ability to conduct normal life functions.
   - This definition is not intended to include experiences of relatively minor medical significance such as uncomplicated headache, nausea, vomiting, diarrhea, influenza, and accidental trauma (eg, sprained ankle) which may interfere with or prevent everyday life functions but do not constitute a substantial disruption.

e) **Is a congenital anomaly/birth defect**

f) **Other situations:**

   - Medical or scientific judgment should be exercised in deciding whether SAE reporting is appropriate in other situations such as important medical events that may not be immediately life-threatening or result in death or hospitalization but may jeopardize the participant or may require medical or surgical intervention to prevent one of the other outcomes listed in the above definition. These events should usually be considered serious.

- Examples of such events include invasive or malignant cancers, intensive treatment in an emergency room or at home for allergic bronchospasm, blood dyscrasias or convulsions that do not result in hospitalization, or development of drug dependency or drug abuse.

RECORDING AND FOLLOW-UP OF AE AND/OR SAE

AE and SAE recording

- When an AE/SAE occurs, it is the responsibility of the investigator to review all documentation (eg, hospital progress notes, laboratory reports, and diagnostics reports) related to the event.
- The investigator will then record all relevant AE/SAE information in the e-CRF.
- It is **not** acceptable for the investigator to send photocopies of the participant's medical records in lieu of completion of the AE/SAE e-CRF page.
- There may be instances when copies of medical records for certain cases are requested by the monitoring team. In this case, all participant identifiers, with the exception of the participant number, will be redacted on the copies of the medical records before submission to the monitoring team.
- The investigator will attempt to establish a diagnosis of the event based on signs, symptoms, and/or other clinical information. Whenever possible, the diagnosis (not the individual signs/symptoms) will be documented as the AE/SAE.

Assessment of intensity

The investigator will make an assessment of intensity for each AE and SAE reported during the study and assign it to 1 of the following categories:

- Mild: An event that is easily tolerated by the participant, causing minimal discomfort and not interfering with everyday activities.
- Moderate: An event that causes sufficiently discomfort and interferes with normal everyday activities.
- Severe: An event that prevents normal everyday activities. An AE that is assessed as severe should not be confused with a SAE. Severe is a category utilized for rating the intensity of an event; and both AEs and SAEs can be assessed as severe.

An event is defined as 'serious' when it meets at least 1 of the predefined outcomes as described in the definition of an SAE, NOT when it is rated as severe.

Assessment of causality

- The investigator is obligated to assess the relationship between study treatment and each occurrence of each AE/SAE.
- A "reasonable possibility" of a relationship conveys that there are facts, evidence, and/or arguments to suggest a causal relationship, rather than a relationship cannot be ruled out.

- The investigator will use clinical judgment to determine the relationship.
- Alternative causes, such as underlying disease(s), concomitant therapy, and other risk factors, as well as the temporal relationship of the event to study treatment administration will be considered and investigated.
- The investigator will also consult the Investigator’s Brochure (IB) and/or Product Information, for marketed products, in his/her assessment.
- For each AE/SAE, the investigator **must** document in the medical notes that he/she has reviewed the AE/SAE and has provided an assessment of causality.
- There may be situations in which an SAE has occurred and the investigator has minimal information to include in the initial report to the monitoring team . However, **it is very important that the investigator always make an assessment of causality for every event before the initial transmission of the SAE data to the monitoring team.**
- The investigator may change his/her opinion of causality in light of follow-up information and send a SAE follow-up report with the updated causality assessment.
- The causality assessment is one of the criteria used when determining regulatory reporting requirements.

Follow-up of AEs and SAEs

- The investigator is obligated to perform or arrange for the conduct of supplemental measurements and/or evaluations as medically indicated or as requested by the monitoring team to elucidate the nature and/or causality of the AE or SAE as fully as possible. This may include additional laboratory tests or investigations, histopathological examinations, or consultation with other health care professionals.
- If a participant dies during participation in the study or during a recognized follow-up period, the investigator will provide the monitoring team with a copy of any post-mortem findings including histopathology.
- New or updated information will be recorded in the originally completed e-CRF.
- The investigator will submit any updated SAE data to the sponsor within 24 hours of receipt of the information.

REPORTING OF SAES

SAE reporting to the monitoring team via an electronic data collection tool

- The primary mechanism for reporting an SAE to the monitoring team will be the electronic data collection tool.
- If the electronic system is unavailable for more than 24 hours, then the site will use the paper SAE data collection tool (see next section).
- The site will enter the SAE data into the electronic system as soon as it becomes available.
- After the study is completed at a given site, the electronic data collection tool will be taken off-line to prevent the entry of new data or changes to existing data.

- If a site receives a report of a new SAE from a study participant or receives updated data on a previously reported SAE after the electronic data collection tool has been taken off-line, then the site can report this information on a paper SAE form (see next section) or to the monitoring team by telephone.
- Contacts for SAE reporting can be found in the clinical trial protocol.

SAE reporting to the monitoring team via paper CRF

- Facsimile transmission of the SAE paper CRF is the preferred method to transmit this information to the monitoring team.
- In rare circumstances and in the absence of facsimile equipment, notification by telephone is acceptable with a copy of the SAE data collection tool sent by overnight mail or courier service.
- Initial notification via telephone does not replace the need for the investigator to complete and sign the SAE CRF pages within the designated reporting time frames.
- Contacts for SAE reporting can be found in the clinical trial protocol.

Appendix 5 Contraceptive guidance and collection of pregnancy Information

DEFINITIONS

Woman of childbearing potential (WOCBP)

A woman is considered fertile following menarche and until becoming post-menopausal unless permanently sterile (see below).

Women in the following categories are not considered WOCBP

1. Premenarchal
2. Premenopausal female with 1 of the following:
    - Documented hysterectomy
    - Documented bilateral salpingectomy
    - Documented bilateral oophorectomy

    Note: Documentation can come from the site personnel's: review of the participant's medical records, medical examination, or medical history interview.
3. Postmenopausal female
    - A postmenopausal state is defined as no menses for 12 months without an alternative medical cause. A high follicle stimulating hormone (FSH) level in the postmenopausal range may be used to confirm a postmenopausal state in women not using hormonal contraception or hormonal replacement therapy (HRT). However, in the absence of 12 months of amenorrhea, a single FSH measurement is insufficient.
    - Females on HRT and whose menopausal status is in doubt will be required to use one of the non-hormonal highly effective contraception methods if they wish to continue their HRT during the study. Otherwise, they must discontinue HRT to allow confirmation of postmenopausal status before study enrollment.

CONTRACEPTION GUIDANCE

Male participants

- Male participants with female partners of childbearing potential are eligible to participate if they agree to ONE of the following for 3 months after the last dose of study treatment:
    - Are abstinent from penile-vaginal intercourse as their usual and preferred lifestyle (abstinent on a long term and persistent basis) and agree to remain abstinent
    - Agree to use a male condom plus partner use of a contraceptive method with a failure rate of <1% per year as described in Table 12 when having penile-vaginal intercourse with a woman of childbearing potential who is not currently pregnant

- In addition male participants must refrain from donating sperm for the duration of the study and for 3 months after the last dose of study treatment
- Male participants with a pregnant or breastfeeding partner must agree to remain abstinent from penile-vaginal intercourse or use a male condom during each episode of penile penetration for 3 months after the last dose of study treatment

Female participants

Female participants of childbearing potential are eligible to participate if they agree to use a highly effective method of contraception consistently and correctly as described in Table 12.

Table 12 - Highly effective contraceptive methods

**Highly effective contraceptive methods that are user dependent**[a]<br>*Failure rate of <1% per year when used consistently and correctly.*
Combined (estrogen and progestogen containing) hormonal contraception associated with inhibition of ovulation[b]<br>• Oral<br>• Intravaginal<br>• Transdermal
Progestogen only hormonal contraception associated with inhibition of ovulation[b]<br>• Oral<br>• Injectable
**Highly effective methods that are user independent**[a]
Implantable progestogen only hormonal contraception associated with inhibition of ovulation[b]<br>• Intrauterine device (IUD)<br>• Intrauterine hormone-releasing system (IUS)<br>Bilateral tubal occlusion
**Vasectomized partner**<br>*A vasectomized partner is a highly effective contraception method provided that the partner is the sole male sexual partner of the WOCBP and the absence of sperm has been confirmed. If not, an additional highly effective method of contraception should be used.*
**Sexual abstinence**<br>*Sexual abstinence is considered a highly effective method only if defined as refraining from heterosexual intercourse during the entire period of risk associated with the study treatment. The reliability of sexual abstinence needs to be evaluated in relation to the duration of the study and the preferred and usual lifestyle of the participant.*

NOTES:
a Typical use failure rates may differ from those when used consistently and correctly. Use should be consistent with local regulations regarding the use of contraceptive methods for participants participating in clinical studies.
b Hormonal contraception may be susceptible to interaction with the study treatment, which may reduce the efficacy of the contraceptive method. In this case, two highly effective methods of contraception should be utilized during the treatment period and for at least 3 months after the last dose of study treatment

PREGNANCY TESTING

- WOCBP should only be included after a confirmed menstrual period and a negative highly sensitive urine pregnancy test.
- Additional pregnancy testing will be performed on Day 28 and as required locally.
- Pregnancy testing will be performed whenever a menstrual cycle is missed or when pregnancy is otherwise suspected
- Urinary pregnancy testing will be performed locally

COLLECTION OF PREGNANCY INFORMATION

Male participants with partners who become pregnant

- The investigator will attempt to collect pregnancy information on any male participant's female partner who becomes pregnant while the male participant is in this study. This applies only to male participants who receive IMP.
- After obtaining the necessary signed informed consent from the pregnant female partner directly, the investigator will record pregnancy information on the appropriate form and submit it to the sponsor within 24 hours of learning of the partner's pregnancy. The female partner will also be followed to determine the outcome of the pregnancy. Information on the status of the mother and child will be forwarded to the sponsor. Generally, the follow-up will be no longer than 6 to 8 weeks following the estimated delivery date. Any termination of the pregnancy will be reported regardless of fetal status (presence or absence of anomalies) or indication for the procedure.

Female Participants who become pregnant

- The investigator will collect pregnancy information on any female participant who becomes pregnant while participating in this study. Information will be recorded on the appropriate form and submitted to the sponsor within 24 hours of learning of a participant's pregnancy. The participant will be followed to determine the outcome of the pregnancy. The investigator will collect follow-up information on the participant and the neonate and the information will be forwarded to the sponsor. Generally, follow-up will not be required for longer than 6 to 8 weeks beyond the estimated delivery date. Any termination of pregnancy will be reported, regardless of fetal status (presence or absence of anomalies) or indication for the procedure.
- Any pregnancy complication or elective termination of a pregnancy will be reported as an AE or SAE. A spontaneous abortion is always considered to be an SAE and will be reported as such. Any post-study pregnancy related SAE considered reasonably related to the study treatment by the investigator will be reported to the sponsor as described in Section 9.2. While the investigator is not obligated to actively seek this information in former study participants, he or she may learn of an SAE through spontaneous reporting.
- Any female participant who becomes pregnant while participating in the study will not be withdrawn from the study.

Appendix 6 Forbidden medication

CYPs inducer/inhibitor/substrate, P-gp substrate (non-exhaustive list)

Compound international nonproprietary name (INN)	Terminal half life	Reason for exclusion
Aliskiren	38h	Pgp substrate
Amiodarone	25 days	CYP2C and CYP3A inhibitor
Atomoxetine	20h	main CYP2D6 substrate
Avasimibe	24h	CYP inducer
Boceprevir	3h	Strong CYP3A inhibitor
Bosentan	7h	CYP inducer
Carbamazepine	15h	CYP inducer
Carvedilol	2h	main CYP2D6 substrate
Chlorpromazine	30h	main CYP2D6 substrate
Clarithromycin	3h	Strong CYP3A inhibitor
Colchicine	31h	Pgp substrate
Conivaptan	5h	Strong CYP3A inhibitor
Dabigatran etexilate	11h	Pgp substrate
Danoprevir/ritonavir	2h/5h	Strong CYP3A inhibitor
Desipramine	28h	main CYP2D6 substrate
Dextromethorphan	3h	main CYP2D6 substrate
Digoxin	36h	Pgp substrate
Efavirenz	3 days	CYP inducer
Elvitegravir/ritonavir	8h/5h	Strong CYP3A inhibitor
Encainide	3h	main CYP2D6 substrate
Enzalutamide	6h	CYP inducer
Etoposide	8h	Pgp substrate
Etravirine	40h	CYP inducer
Everolimus	30h	Pgp substrate
Fexofenadine	15h	Pgp substrate
Flecainide	11h	main CYP2D6 substrate
Fluconazole	32h	Strong CYP2C inhibitor
Fluoxetine	2 days	CYP2C and CYP3A inhibitor
Fluvoxamine	15h	Strong CYP2C inhibitor

Compound international nonproprietary name (INN)	Terminal half life	Reason for exclusion
Gemfibrozil	1h	Strong CYP2C inhibitor
Genistein	8h	CYP inducer
Grapefruit juice	-	Strong CYP3A inhibitor
Imipramine	16h	main CYP2D6 substrate
Indinavir, Indinavir/ritonavir	2h/5h	Strong CYP3A inhibitor
Itraconazole	21h	Strong CYP3A inhibitor
Ketoconazole	3h	Strong CYP3A inhibitor
Lapatinib	14h	Pgp substrate
Lersivirine	6h	CYP inducer
Linezolide	5h	Pgp substrate
Loperamide	11h	Pgp substrate
Lopinavir, Lopinavir/ritonavir	5h	Strong CYP3A inhibitor, CYP inducer
Maprotiline	45h	main CYP2D6 substrate
Maraviroc	18h	Pgp substrate
Metoprolol	3h	main CYP2D6 substrate
Mibefradil	22h	Strong CYP3A inhibitor
Miconazole	24h	Strong CYP2C inhibitor
Mitotane	18-159 days	Potent CYP inducer
Modafinil	11h	CYP inducer
Nafcillin	2h	CYP inducer
Nebivolol	11h	main CYP2D6 substrate
Nefazodone	4h	Strong CYP3A inhibitor
Nelfinavir	5h	Strong CYP3A inhibitor
Nilotinib	17h	Pgp substrate
Nortriptyline	31h	main CYP2D6 substrate
Paroxetine	17h	main CYP2D6 substrate
Perphenazine	9h	main CYP2D6 substrate
Phenobarbital	4 days	CYP inducer
Phenytoine	24h	CYP inducer
Pimozide	5 days	main CYP2D6 substrate
Posaconazole	35h	Strong CYP3A inhibitor, Pgp substrate
Propafenone	6h	main CYP2D6 substrate

Compound international nonproprietary name (INN)	Terminal half life	Reason for exclusion
Propranolol	4h	main CYP2D6 substrate
Ranolazine	7h	Pgp substrate
Rifabutine	28-62h	CYP inducer
Rifampin	4h	CYP inducer
Rifapentine	18h	CYP inducer
Risperidone	3h	main CYP2D6 substrate
Ritonavir	5h	Strong CYP3A inhibitor
Saquinavir, Saquinavir/ritonavir	12h/5h	Strong CYP3A inhibitor, Pgp substrate
Semagacestat	2.5h	CYP inducer
Sirolimus	62h	Pgp substrate
St John's wort	-	CYP inducer
Talinolol	9h	Pgp substrate
Talviraline	-	CYP inducer
Telaprevir	11h	Strong CYP3A inhibitor
Telithromycin	12h	Strong CYP3A inhibitor
Thioridazine	24h	main CYP2D6 substrate
Ticlopidine	4 days	Strong CYP2C inhibitor
Timolol	3h	main CYP2D6 substrate
Tipranavir/ritonavir	6h/5h	Strong CYP3A inhibitor
Tolterodine	2h	main CYP2D6 substrate
Tolvaptan	12h	Pgp substrate
Trimipramine	24h	main CYP2D6 substrate
Troleandomycine	3h	Strong CYP3A inhibitor
Venlafaxine	5h	main CYP2D6 substrate
Voriconazole	7h	Strong CYP3A inhibitor

Drugs known to modify QT interval (non-exhaustive list)

Generic Name	Risk[a]	Brand Name
Alfuzosin	PR	Uroxatral
Amantadine	CR	Symmetrel and others
Amiodarone	KR	Cordarone and others

Generic Name	Risk[a]	Brand Name
Amisulpride	CR	Solian and others
Amitriptyline	CR	Elavil (Discontinued 6/13) and others
Amphotericin B	CR	Fungilin and others
Anagrelide	KR	Agrylin and others
Apomorphine	PR	Apokyn and others
Aripiprazole	PR	Abilify and others
Arsenic trioxide	KR	Trisenox
Artenimol+piperaquine	PR	Eurartesim
Asenapine	PR	Saphris and others
Astemizole	KR	Hismanal
Atazanavir	CR	Reyataz and others
Atomoxetine	PR	Strattera
Azithromycin	KR	Zithromax and others
Bedaquiline	PR	Sirturo
Bendamustine	PR	Treanda and others
Bendroflumethiazide or bendrofluazide	CR	Aprinox
Bepridil	KR	Vascor
Bortezomib	PR	Velcade and others
Bosutinib	PR	Bosulif
Buprenorphine	PR	Butrans and others
Capecitabine	PR	Xeloda
Ceritinib	PR	Zykadia
Chloral hydrate	CR	Aquachloral and others
Chloroquine	KR	Aralen
Chlorpromazine	KR	Thorazine and others
Cilostazol	KR	Pletal
Ciprofloxacin	KR	Cipro and others
Cisapride	KR	Propulsid
Citalopram	KR	Celexa and others
Clarithromycin	KR	Biaxin and others
Clomipramine	PR	Anafranil
Clozapine	PR	Clozaril and others
Cocaine	KR	Cocaine
Crizotinib	PR	Xalkori

Generic Name	Risk[a]	Brand Name
Cyamemazine (cyamepromazine)	PR	Tercian
Dabrafenib	PR	Tafinlar
Dasatinib	PR	Sprycel
Degarelix	PR	Firmagon
Delamanid	PR	Deltyba
Desipramine	PR	Pertofrane and others
Deutetrabenazine	PR	Austedo
Dexmedetomidine	PR	Precedex and others
Diphenhydramine	CR	Benadryl and others
Disopyramide	KR	Norpace
Dofetilide	KR	Tikosyn
Dolasetron	PR	Anzemet
Domperidone	KR	Motilium and others
Donepezil	KR	Aricept
Doxepin	CR	Sinequan and others
Dronedarone	KR	Multaq
Droperidol	KR	Inapsine and others
Efavirenz	PR	Sustiva and others
Eliglustat	PR	Cerdelga
Eribulin mesylate	PR	Halaven
Erythromycin	KR	E.E.S. and others
Escitalopram	KR	Cipralex and others
Esomeprazole	CR	Nexium and others
Ezogabine (Retigabine)	PR	Potiga and others
Famotidine	PR	Pepcid and others
Felbamate	PR	Felbatol
Fingolimod	PR	Gilenya
Flecainide	KR	Tambocor and others
Fluconazole	KR	Diflucan and others
Fluoxetine	CR	Prozac and others
Flupentixol	PR	Depixol and others
Fluvoxamine	CR	Faverin and others
Furosemide (frusemide)	CR	Lasix and others
Galantamine	CR	Reminyl and others

Generic Name	Risk[a]	Brand Name
Garenoxacin	CR	Geninax
Gatifloxacin	KR	Tequin
Gemifloxacin	PR	Factive
Granisetron	PR	Kytril and others
Grepafloxacin	KR	Raxar
Halofantrine	KR	Halfan
Haloperidol	KR	Haldol (US & UK) and others
Hydrochlorothiazide	CR	Apo-Hydro and others
Hydrocodone - ER	PR	Hysinglaâ„¢ ER and others
Hydroxychloroquine	CR	Plaquenil and others
Hydroxyzine	CR	Atarax and others
Ibogaine	KR	None
Ibutilide	KR	Corvert
Iloperidone	PR	Fanapt and others
Imipramine (melipramine)	PR	Tofranil
Indapamide	CR	Lozol and others
Isradipine	PR	Dynacirc
Itraconazole	CR	Sporanox and others
Ivabradine	CR	Procoralan and others
Ketanserin	PR	Sufrexal
Ketoconazole	CR	Nizoral and others
Lansoprazole	CR	Prevacid
Lapatinib	PR	Tykerb and others
Lenvatinib	PR	Lenvima
Leuprolide	PR	Lupron and others
Levofloxacin	KR	Levaquin and others
Levomepromazine	KR	Nosinan and others
Levomethadyl acetate	KR	Orlaam
Levosulpiride	KR	Lesuride and others
Lithium	PR	Eskalith and others
Loperamide	CR	Imodium and many other OTC and Rx brands
Melperone	PR	Bunil and others
Mesoridazine	KR	Serentil
Methadone	KR	Dolophine and others

Generic Name	Risk[a]	Brand Name
Metoclopramide	CR	Reglan and others
Metronidazole	CR	Flagyl and many others
Midostaurin	PR	Rydapt
Mifepristone	PR	Korlym and others
Mirabegron	PR	Myrbetriq
Mirtazapine	PR	Remeron
Moexipril/HCTZ	PR	Uniretic and others
Moxifloxacin	KR	Avelox and others
Necitumumab	PR	Portrazza
Nelfinavir	CR	Viracept
Nicardipine	PR	Cardene
Nilotinib	PR	Tasigna
Norfloxacin	PR	Noroxin and others
Nortriptyline	PR	Pamelor and others
Nusinersen	PR	Spinraza
Ofloxacin	PR	Floxin
Olanzapine	CR	Zyprexa and others
Omeprazole	CR	Losec and others
Ondansetron	KR	Zofran and others
Osimertinib	PR	Tagrisso
Oxaliplatin	KR	Eloxatin
Oxytocin	PR	Pitocin and others
Paliperidone	PR	Invega and others
Panobinostat	PR	Farydak
Pantoprazole	CR	Protonix and others
Papaverine HCl (Intra-coronary)	KR	none
Paroxetine	CR	Paxil and others
Pasireotide	PR	Signifor
Pazopanib	PR	Votrient
Pentamidine	KR	Pentam
Perflutren lipid microspheres	PR	Definity and others
Perphenazine	PR	Trilafon and others
Pilsicainide	PR	Sunrythm
Pimavanserin	PR	Nuplazid

Generic Name	Risk[a]	Brand Name
Pimozide	KR	Orap
Pipamperone	PR	Dipiperon (E.U) and others
Posaconazole	CR	Noxafil and others
Probucol	KR	Lorelco
Procainamide	KR	Pronestyl and others
Promethazine	PR	Phenergan
Propofol	KR	Diprivan and others
Prothipendyl	PR	Dominal and others
Quetiapine	CR	Seroquel
Quinidine	KR	Quinaglute and others
Quinine sulfate	CR	Qualaquin
Ranolazine	CR	Ranexa and others
Ribociclib	PR	Kisqali
Rilpivirine	PR	Edurant and others
Risperidone	PR	Risperdal
Ritonavir	CR	Norvir
Romidepsin	PR	Istodax
Roxithromycin	KR	Rulide and others
Saquinavir	PR	Invirase (combo)
Sertindole	PR	Serdolect and others
Sertraline	CR	Zoloft and others
Sevoflurane	KR	Ultane and others
Solifenacin	CR	Vesicare
Sorafenib	PR	Nexavar
Sotalol	KR	Betapace and others
Sparfloxacin	KR	Zagam
Sulpiride	KR	Dogmatil and others
Sultopride	KR	Barnetil and others
Sunitinib	PR	Sutent
Tacrolimus	PR	Prograf and others
Tamoxifen	PR	Nolvadex (discontinued 6/13) and others
Telaprevir	CR	Incivo and others
Telavancin	PR	Vibativ
Telithromycin	PR	Ketek

Generic Name	Risk[a]	Brand Name
Terfenadine	KR	Seldane
Terlipressin	KR	Teripress and others
Terodiline	KR	Micturin and others
Tetrabenazine	PR	Nitoman and others
Thioridazine	KR	Mellaril and others
Tiapride	PR	Tiapridal and others
Tipiracil and Trifluridine	PR	Lonsurf
Tizanidine	PR	Zanaflex and others
Tolterodine	PR	Detrol and others
Toremifene	PR	Fareston
Torsemide	CR	Demadex and others
Trazodone	CR	Desyrel (discontinued 6/13) and others
Trimipramine	PR	Surmontil and others
Tropisetron	PR	Navoban and others
Valbenazine	PR	Ingrezza
Vandetanib	KR	Caprelsa
Vardenafil	PR	Levitra
Vemurafenib	PR	Zelboraf
Venlafaxine	PR	Effexor and others
Voriconazole	CR	VFend
Vorinostat	PR	Zolinza
Ziprasidone	CR	Geodon and others
Zotepine	PR	Losizopilon and others

a KR: Known Risk of Torsade de Pointe (TdP); PR: Possible Risk of TdP; CR: Conditional Risk of TdP

Source: https://www.crediblemeds.org/pdftemp/pdf/CombinedList.pdf, list last reviewed on 22-July-2017.

Appendix 7 Liver and other safety: suggested actions and follow-up assessments

NEUTROPENIA

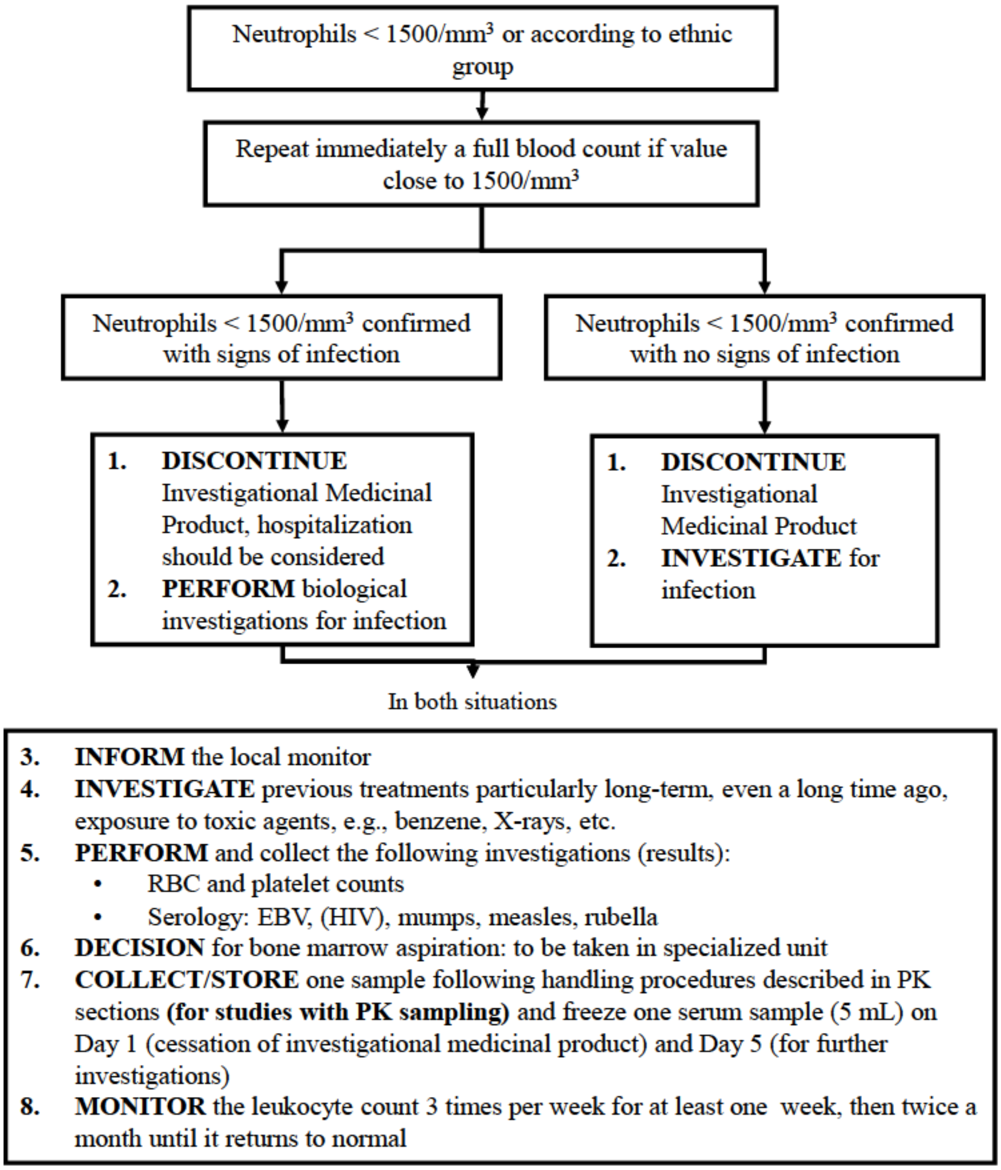

Note:

•The procedures described in the above flowchart are to be discussed with the patient only in case the event occurs. If applicable (according to local regulations), an additional consent (e.g., for HIV testing) will only be obtained in the case the event actually occurs.

•For individuals of African descent, the relevant value of concern is <1000/mm3

Neutropenia is to be recorded as an AE only if at least 1 of the criteria listed in the general guidelines for reporting adverse events in Appendix 4 is met. Due to single dose regimen, instructions regarding IMP discontinuation/resumption do not apply.

THROMBOCYTOPENIA

Platelets < 100 000/mm³ (rule out EDTA – induced pseudo-thrombocytopenia)

↓

Repeat immediately the count (rule out EDTA anticoagulant in the sample)

↓

Platelets < 100 000/mm³ confirmed with bleeding	Platelets < 100 000/mm³ confirmed with no bleeding
1. **DISCONTINUE** Investigational Medicinal Product<br>2. **HOSPITALIZATION** should be considered	1. **DISCONTINUE** Investigational Medicinal Product<br>2. **INVESTIGATE** for bleeding

In both situations

3. **INFORM** the local Monitor
4. **QUESTION** about last intake of quinine (drinks), alcoholism, heparin administration
5. **PERFORM** or collect the following investigations:
   - Complete blood count, schizocytes, creatinine
   - Bleeding time and coagulation test (fibrinogen, INR or PT, aPTT), Fibrin Degradation Product
   - Viral serology: EBV, HIV, mumps, measles, rubella
6. **COLLECT/STORE** one sample following handling procedures described in PK sections **(for studies with PK sampling)** and freeze one serum sample (5 mL) on Day 1 (cessation of investigational medicinal product) and Day 5 (for further investigations)
7. **DECISION** for bone marrow aspiration: to be taken in specialized unit
   - On Day 1 in the case of associated anemia and/or leukopenia
   - On Day 8 if platelets remain < 50 000/mm³
8. **MONITOR** the platelet count every day for at least one week and then regularly until it returns to normal

Note:
The procedures above flowchart are to be discussed with the patient only in case described in the the event occurs. If applicable (according to local regulations), an additional consent (e.g., for HIV testing) will only be obtained in the case the event actually occurs.

For patients with low platelets count (platelets <100,000 /mm^3), after immediately repeating the count (rule out EDTA anticoagulant in the sample), the instructions from point 2 to 8 must be followed, if at least one of the following situations is present:

- If the confirmed decrease (repeat measure) is deemed by the investigator larger than expected due to underlying disease
- 50,000 /mm^3 ≤Platelets <100,000 /mm^3 confirmed with bleeding
- Platelets <50,000 /mm^3 confirmed

Thrombocytopenia is to be recorded as an AE only if at least 1 of the criteria listed in the general guidelines for reporting adverse events in Appendix 4 is met. Due to single dose regimen, instructions regarding IMP discontinuation/resumption do not apply.

INCREASE IN ALT

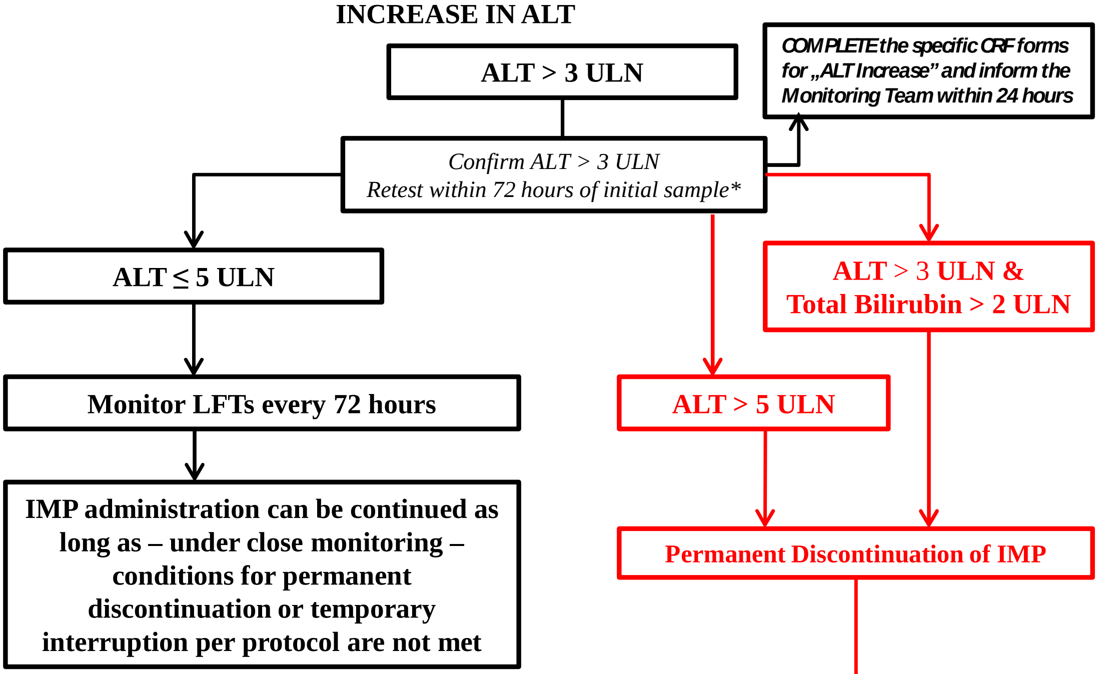

In ANY CASE, FOLLOW the instructions listed in the box below:

1. **INFORM** the Site Monitor who will forward the information to the Study Manager
2. **INVESTIGATE** specifically for malaise with or without loss of consciousness, dizziness, and/or hypotension and/or episode of arrhythmia in the previous 72 hours; rule out muscular injury
3. **PERFORM** the following tests:
   - LFTs: AST, ALT, alkaline phosphatase, total and conjugated bilirubin and prothrombin time / INR
   - CPK, serum creatinine, complete blood count
   - Anti-HAV IgM, anti-HBc IgM, (HBV-DNA if clinically indicated), anti-HCV and HCV RNA, anti-CMV IgM and anti-HEV IgM antibodies
   - Depending on the clinical context, check for recent infection with EBV, herpes viruses, and toxoplasma
   - Hepatobiliary ultrasonography (or other imaging investigations if needed)
4. **CONSIDER** Auto-antibodies: antinuclear, anti-DNA, anti-smooth muscle, anti-LKM
5. **CONSIDER** consulting with hepatologist
6. **CONSIDER** patient hospitalisation if INR>2 (or PT<50%) and/or central nervous system disburbances suggesting hepatic encephalopathy
7. **MONITOR LFTs after discontinuation of IMP:**
   - As *closely as possible* (or **every 48 hours)** until stabilization, then every 2 weeks until return to normal/baseline or clinical resolution.
8. **FREEZE** serum sample (5ml x 2)
9. **In case of SUSPICION of GILBERT Syndrome,** a DNA diagnostic test should be done

*If unable to retest in 72 hours, use original lab results to decide on further reporting/monitoring/discontinuation.

Note:

- "Baseline" refers to ALT sampled at baseline visit; or if baseline value unavailable, to the latest ALT sampled before the baseline visit. The algorithm does not apply to the instances of increase in ALT during screening.
- See Appendix 4 for guidance on safety reporting.
- Normalization is defined as ≤ ULN or baseline value, if baseline value is >ULN.
- Due to single dose regimen, instructions regarding IMP discontinuation/resumption do not apply.

INCREASE IN SERUM CREATININE

Rapid increase in serum creatinine over 150 µmol/L or rapid decrease in creatinine clearance below 50 mL/mn

Can be rapidly reversed:
- By volume repletion
- Or relief of urinary tract obstruction (according to etiology)

1. Investigational medicinal product may be continued
2. **MONITOR** serum creatinine until return to baseline level

Cannot be rapidly reversed:
- Occurrence/aggravation of life threatening symptoms of ARF: anemia, hyperkalemia, hyperuricemia, metabolic acidosis, cardiac insufficiency, pulmonary edema, arrhythmia, DIC, etc.
- And/or predominant elimination of investigational medicinal product by renal route

1. **INFORM** the local monitor
2. **DISCONTINUE** investigational medicinal product administration
3. **HOSPITALIZATION** should be considered and seek for nephrologic advice
4. **PERFORM** the following examinations:
   - BP, HR, hydration status, ECG
   - Blood count
   - Liver function tests + CPK
   - Biochemistry, including urea
   - Urinalysis
5. **COLLECT/STORE** one sample following handling procedures described in PK sections **(for studies with PK sampling)** and freeze one serum sample (5 mL) on Day 1 (cessation of investigational medicinal product)
6. **MONITOR** renal function until return to baseline level (every day at the beginning, then every week)

Increase in serum creatinine is to be recorded as an AE only if at least 1 of the criteria listed in the general guidelines for reporting adverse events in Appendix 4 is met. Due to single dose regimen, instructions regarding IMP discontinuation/resumption do not apply.

INCREASE IN CPK SUSPECTED TO BE OF NON-CARDIAC ORIGIN AND NOT RELATED TO INTENSIVE PHYSICAL ACTIVITY

Muscular symptoms (myalgia, pain, weakness, dark urines)

Systematic CPK assessment as per protocol

Perform CPK

If Increase in CPK (expressed in ULN)

> 3 ULN

Repeat immediately the count.
If confirmed, inform the local monitor and

INVESTIGATE for the origin:
- **PERFORM**:
    - ECG
    - CPK-MB -MM
    - Troponin
    - Creatinine
    - Iono (k+, Ca^{2}+)
    - Transaminases + Total and conjugated bilirubin
    - Myoglobin (serum and urines)
- **COLLECT/STORE** one sample following handling procedures described in PK sections **(for studies with PK sampling)** and freeze one serum sample (5 mL) on Day 1 (cessation of investigational medicinal product).
- **INTERVIEW** the patient about a recent intensive muscular effort, trauma, convulsions, electrical injury, injury or stress to the skeletal muscle, multiple intramuscular injections, recent surgery, concomitant medications, consumption of alcohol, morphine, cocaine.
- **SEARCH** for alternative causes to cardiac or muscular toxicity, ie, stroke, pulmonary infarction, dermatomyositis or polymyositis, convulsions, hypothyroidism, delirium tremens, muscular dystrophies.

If either the cardiac origin or the rhabdomyolysis is confirmed or if CPK > 10 ULN:
1. **DISCONTINUE** investigational medicinal product administration
2. **MONITOR** CPK every 3 days for the first week then once weekly until return to normal or for at least 3 months
3. **HOSPITALIZATION** should be considered

If the cardiac origin or the rhabdomyolysis is ruled out and if CPK ≤ 10 ULN:
MONITOR CPK every 3 days for the first week then once weekly until return to normal or for at least 3 months

Increase in CPK is to be recorded as an AE only if at least 1 of the criteria in the general guidelines for reporting adverse events in Appendix 4 is met. Due to single dose regimen, instructions regarding IMP discontinuation/resumption do not apply.

Appendix 8 Sample size determination

Part 1: Estimated efficacy (Day 28 PCR-corrected ACPR) of FQ 200, 400, 600 and 900 mg administrated in presence of OZ439 TPGS for different levels of baseline parasitemia

Clinical trial simulations were used, consisting of simulating FQ concentrations at Day 7, baseline parasitemia, and then PCR-corrected ACPR at Day 28 using a model linking ACPR at Day 28 probability to FQ concentration at Day 7 and baseline parasitemia.

The model (eq 1) was a logistic regression modeling PCR corrected ACPR at Day 28 probability (p) as a function of FQ concentration at Day 7 (ConcD7FQ), Artesunate (AR) indicator (no/yes) and baseline log10-parasitemia. It was developed from DRI10382 data: African patients, Per Protocol population, excluding patients with reinfections, using the 4 arms of the DRI10382 (FQ 4 mg/kg or AR + FQ 2, 4 or 6 mg/kg, repeated doses). Two types of analyses were done, classifying as failure or excluding patients with rescue therapy before/without failure, and finally the results of the analysis excluding patients were favored, to compensate for the possible under-estimation of FQ single dose effect (model built on repeated doses). In this analysis based on 223 patients, the FQ efficacy was estimated to be ACPR28corr=80.9% (95% Confidence Interval [CI]: 66.7% to 90.9%) in arm FQ 600 mg.

(eq 1) $\log(p/(1-p)) = \alpha + \beta FQ.ConcD7FQ + \beta AR. (AR=yes) + \beta BasePar.\log10(BasePar)$

FQ concentrations at Day 7 (168 h post dose) were simulated from the FQ PopPK model updated from DRI12805 (POH0456) for TPGS formulation and single dose administration, in an African adult population (weights resampled from 194 profiles of African adults of DRI10382 and DRI12805 cohort 1A and 1B, of median weight 55 kg and median age 21 years). Log-normal distributions were fitted to these simulated concentrations and then used for simulation.

Baseline parasitemia was simulated for different scenarii, using truncated log-normal distributions. The base assumption was a baseline parasitemia greater than 1000 parasites/µL and of median about 10,000 parasites/µL. Some other scenarii were considered: baseline parasitemia greater than 3000, 5000, or 10,000 parasites/µL, with a maximum fixed at 5.5 log10 parasites/µL. In the following, the baseline parasitemia greater than 3000 parasites/µL scenario was selected to ensure a better enrollment of patients during the study.

Based on N=10,000 simulations/arm and S=1000 simulations for the model parameters uncertainty, the results showed a PCR-corrected ACPR at Day 28 estimated at 72% (90%CI: [57% to 84%]) for FQ 400mg alone and 79% (90%CI: 67% to 87%) for FQ 600 mg alone for a baseline parasitemia greater than 3000 parasites/µL. Finally the FQ fixed dose of 400 mg was selected.

Part 2: Estimated power for various sample size of an OZ439 exposure-effect analysis, considering the fixed FQ dose, the 4 selected OZ439 dose levels (0, 300, 600, 1000 mg) and the selected baseline parasitemia

Simulation of clinical trials

Clinical trial simulations were used to simulate trials of 4 arms with a fixed dose of 400 mg FQ and 4 dose levels of OZ439 (0, 300, 600, and 1000 mg) for a baseline parasitemia greater than 3000 parasites/µL. FQ and OZ439 concentrations at Day 7 (168 h post dose) were simulated using log-normal distributions that were adjusted to concentrations obtained from the FQ PopPK model and from the OZ439 PopPK model in an African adult population (as in part 1), and were assumed to be slightly correlated. Truncated log-normal distributions were used to simulate baseline parasitemia greater than 3000 parasites/µL in log10Par/µL by a normal distribution: mean=4, SD=0.55, minimum=3.48; maximum=5.5 (minimum and maximum for the truncation). Day 28 PCR-corrected ACPR was simulated using a “hypothetical” model linking ACPR at Day 28 probability to FQ and OZ439 concentration at Day 7 and baseline parasitemia.

The “hypothetical” logistic regression model (eq 2, see below) used for simulations was a composite of the “PQP/OZ-ACPR28 model” built by MMV, modeling PCR-corrected ACPR at Day 28 probability (p) as a function of OZ439 and PQP concentrations at Day 7 and baseline log10-parasitemia, and of assumptions made on FQ efficacy, which were based on the results of part 1. Precisely, βFQ was fixed at 0.12 by calibration to get ~ 72% success ACPR at Day 28 for FQ 400 mg alone for a baseline parasitemia greater than 3000 parasites/µL, and the following parameters were extracted from the MMV model: α=3.23, βOZ439 = 0.73, and βBasePar = -1.27.

(eq 2) $\log(p/(1-p)) = \alpha + \beta FQ.ConcD7FQ + \beta OZ439.\ ConcD7OZ + \beta BasePar.\log10(BasePar)$

Using all these assumptions, the PCR-corrected ACPR at Day 28 rates simulated in the 4 arms FQ 400 mg + OZ439 at 0, 300, 600 and 1000 mg were of 72%, 81%, 91%, and 97% for a baseline parasitemia greater than 3000 parasites/µL.

Clinical trials analyses

Exposure-effect analyses of the simulated clinical trials were performed, consisting of a logistic regression model of exposure at Day 7 for both drugs, with baseline parasitemia as covariate, and the Day 28 PCR-corrected ACPR as response variable (as in eq 2 above). Significance of OZ439 concentration effect (βOZ439) was analyzed using a 2-sided alpha Wald test at 5%.

Overall, five thousand trial simulations were performed, and the power was deduced as the percentage of significant simulated trials. Results estimated that 30 evaluable participants per treatment arm will yield a power around 90% to detect an OZ439 concentration effect relation (in a population of patients with a baseline parasitemia greater than 3000 parasites/µL, for 4 arms FQ 400 mg + OZ439 at 0, 300, 600, and 1000 mg).

Around 15 % of early dropout is anticipated, consequently approximately 35 participants are to be included in each arm (to get 30 evaluable participants/arm) resulting in approximately 140 participants to be enrolled in the whole study.

A rather large uncertainty was attached to the models and to the different assumptions used to perform the simulations, so these results are approximate.

Appendix 9 Country-specific requirements

ACT14655 16.1.1 Protocol

ELECTRONIC SIGNATURES

Signed by	Meaning of Signature	Server Date (dd-MMM-yyyy HH:mm)
[redacted]	[redacted]	[redacted]
[redacted]	[redacted]	[redacted]
