# Supplementary material for: Randomized, open-label, phase 2a study to evaluate the contribution of artefenomel to the clinical and parasiticidal activity of artefenomel plus ferroquine in African patients with uncomplicated Plasmodium falciparum malaria
Source: Malar J. 2023 Jan 3;22:2. doi: 10.1186/s12936-022-04420-2 (PMC9809015; doi:10.1186/s12936-022-04420-2)
Supplement: Supplementary file 2 — Additional file 2: Clinical trial simulation to determine the dose and sample sizes. [file 12936_2022_4420_MOESM2_ESM.pdf]

Additional file 2: Dose selection and sample size

Contents

Overall strategy 2

Part 1: Estimated efficacy (Day 28 PCR-adjusted ACPR) of ferroquine 200, 400, 600 and 900 mg administrated in presence of artefenomel TPGS for different levels of baseline parasitemia 2

Part 2: Estimated power for various sample size of an artefenomel exposure-effect analysis, considering the fixed ferroquine dose, the 4 selected artefenomel dose levels (0, 300, 600, 1000 mg) and the selected baseline parasitemia 3

Simulation of clinical trials 3

Clinical trials simulations 3

References 4

Abbreviations

ACPR, adequate clinical and parasitological response

AR, artesunate

FALCI: ferroquine and artefenomel in adults and children with *Plasmodium falciparum* malaria

FQ, ferroquine

OZ, artefenomel

PCR, polymerase chain reaction

PK, pharmacokinetic

PopPK, population pharmacokinetic

PQP, piperaquine

TPGS, alpha tocopherol polyethylene glycol 1000 succinate

Overall strategy

The strategy for selecting fixed ferroquine dose, baseline parasitemia, and sample size was composed of two parts:

1. Estimated efficacy (Day 28 PCR-adjusted ACPR) of ferroquine 200, 400, 600 and 900 mg administrated in presence of artefenomel alpha tocopherol polyethylene glycol 1000 succinate (TPGS) formulation for different levels of baseline parasitemia.

2. Estimated power for various sample size of an artefenomel exposure-effect analysis, considering the fixed ferroquine dose, the 4 selected artefenomel dose levels (0, 300, 600, 1000 mg) and the selected baseline parasitemia.

Part 1: Estimated efficacy (Day 28 PCR-adjusted ACPR) of ferroquine 200, 400, 600 and 900 mg administrated in presence of artefenomel TPGS for different levels of baseline parasitemia

Clinical trial simulations were used, consisting of simulating ferroquine concentrations at Day 7, baseline parasitemia, and then PCR-adjusted ACPR at Day 28 using a model linking ACPR at Day 28 probability to ferroquine concentration at Day 7 and baseline parasitemia.

The model (eq 1) was a logistic regression modeling PCR-adjusted ACPR at Day 28 probability (p) as a function of ferroquine concentration at Day 7 (ConcD7,FQ), artesunate (AR) indicator (no/yes) and baseline log10-parasitemia. It was developed from data reported in Held, 2015: African patients, per protocol population, excluding patients with reinfections, using the 4 arms of the study (ferroquine 4 mg/kg or AR + ferroquine 2, 4 or 6 mg/kg, repeated doses). Two types of analyses were done, classifying as failure or excluding patients with rescue therapy before/without failure, and finally the results of the analysis excluding patients were favored, to compensate for the possible underestimation of ferroquine single dose effect (model built on repeated doses). In this analysis based on 223 patients, the ferroquine efficacy was estimated to be Day 28 PCR-adjusted ACPR = 80.9% (95% Confidence Interval [CI]: 66.7% to 90.9%) in arm ferroquine 600 mg.

(eq 1) $\log(p/(1-p)) = \alpha + \beta FQ.ConcD7,FQ + \beta AR.\ (AR=yes) + \beta BasePar.\log10(BasePar)$

ferroquine concentrations at Day 7 (168 h post dose) were simulated from the ferroquine PopPK model updated using available data from the FALCI study (Adoke, 2021; Boulu POH0456) for TPGS formulation and single dose administration, in an African adult population (weights resampled from 194 profiles of African adults of Held, 2015 and FALCI (Adoke, 2021: cohort 1A and 1B, of median weight 55 kg and median age 21 years). Log-normal distributions were fitted to these simulated concentrations and then used for simulation.

Baseline parasitemia was simulated for different scenarios, using truncated log-normal distributions. The base assumption was a baseline parasitemia greater than 1000 parasites/µL and of median about 10,000 parasites/µL. Some other scenarios were considered: baseline parasitemia greater than 3000, 5000, or 10,000 parasites/µL, with a maximum fixed at 5.5 log10 parasites/µL (316,228 parasites/µL). In the following, the baseline parasitemia greater than 3000 parasites/µL scenario was selected to ensure a better enrollment of patients during the study.

Based on N=10,000 simulations/arm and S=1000 simulations for the model parameters uncertainty, the results showed a PCR-adjusted ACPR at Day 28 estimated at 72% (90%CI: 57% to 84%) for ferroquine 400 mg alone and 79% (90%CI: 67% to 87%) for ferroquine 600 mg alone for a baseline parasitemia greater than 3000 parasites/µL. Finally, the ferroquine fixed dose of 400 mg was selected.

Part 2: Estimated power for various sample size of an artefenomel exposure-effect analysis, considering the fixed ferroquine dose, the 4 selected artefenomel dose levels (0, 300, 600, 1000 mg) and the selected baseline parasitemia

Simulation of clinical trials

Clinical trial simulations were used to simulate trials of 4 arms with a fixed dose of 400 mg ferroquine and 4 dose levels of artefenomel (0, 300, 600, and 1000 mg) for a baseline parasitemia greater than 3000 parasites/µL. Ferroquine and artefenomel concentrations at Day 7 (168 h post dose) were simulated using log-normal distributions that were adjusted to concentrations obtained from the ferroquine PopPK model and from the artefenomel PopPK model in an African adult population (Boulu, POH0456 as in part 1), and were assumed to be slightly correlated. Truncated log-normal distributions were used to simulate baseline parasitemia greater than 3000 parasites/µL in log10Par/µL by a normal distribution: mean=4, SD=0.55, minimum=3.48; maximum=5.5 (minimum and maximum for the truncation). Day 28 PCR-adjusted ACPR was simulated using a “hypothetical” model linking ACPR at Day 28 probability to ferroquine and artefenomel concentration at Day 7 and baseline parasitemia.

The “hypothetical” logistic regression model (eq 2, see below) used for simulations was a composite of the “PQP/OZ-ACPR28 model” built by Medicines for Malaria Venture (MMV) (reported in Macintyre, 2017), modeling PCR-adjusted ACPR at Day 28 probability (p) as a function of artefenomel and piperaquine (PQP) concentrations at Day 7 and baseline log10-parasitemia, and of assumptions made on ferroquine efficacy, which were based on the results of part 1. Precisely, βFQ was fixed at 0.12 by calibration to get ~ 72% success ACPR at Day 28 for ferroquine 400 mg alone for a baseline parasitemia greater than 3000 parasites/µL, and the following parameters were extracted from the MMV model: α=3.23, βartefenomel = 0.73, and βBasePar = -1.27.

(eq 2) $\log(p/(1-p)) = \alpha + \beta FQ.ConcD7,FQ + \beta artefenomel. ConcD7,OZ + \beta BasePar.\log10(BasePar)$

Using all these assumptions, the PCR-adjusted ACPR at Day 28 rates simulated in the 4 arms ferroquine 400 mg + artefenomel at 0, 300, 600 and 1000 mg were of 72%, 81%, 91%, and 97% for a baseline parasitemia greater than 3000 parasites/µL.

Clinical trials simulations

Exposure–effect analyses of the simulated clinical trials were performed, consisting of a logistic regression model of exposure at Day 7 for both drugs, with baseline parasitemia as covariate, and the Day 28 PCR-adjusted ACPR as response variable (as in eq 2 above). Significance of artefenomel concentration effect (βartefenomel) was analyzed using a 2-sided alpha Wald test at 5%.

Overall, five thousand trial simulations were performed, and the power was deduced as the percentage of significant simulated trials. Results estimated that 30 evaluable participants per treatment arm will yield a power around 90% to detect an artefenomel concentration effect relation (in a population of patients with a baseline parasitemia greater than 3000 parasites/µL, for 4 arms ferroquine 400 mg + artefenomel at 0, 300, 600, and 1000 mg).

Around 15 % of early dropout was anticipated, consequently approximately 35 participants were to be included in each arm (to get 30 evaluable participants/arm) resulting in approximately 140 participants to be enrolled in the whole study.

A rather large uncertainty was attached to the models and to the different assumptions used to perform the simulations, so these results were approximate.

References

Adoke Y, Zoleko-Manego R, Ouoba S, Tiono A, Kaguthi G, Bonzela JE, et al. A randomized, double-blind, phase 2b study to investigate the efficacy, safety, tolerability and pharmacokinetics of a single-dose regimen of ferroquine with artefenomel in adults and children with uncomplicated *Plasmodium falciparum* malaria. Malaria J. 2021; 20:222.

Boulu L. POH0456. Population PK analysis of ferroquine (SSR97193), and its metabolite SSR97213 from a pool of phase 1 and 2 studies (TDU5419, TDU5967, TDR5969, INT6856, ACT10420, DRI10382, TDU12511 and DRI12805). Sanofi. Internal Report, 2016.

Held J, Supan C, Salazar CL, Tinto H, Bonkian LN, Nahum A, et al: Ferroquine and artesunate in African adults and children with *Plasmodium falciparum* malaria: a phase 2, multicentre, randomised, double-blind, dose-ranging, non-inferiority study. Lancet Infect Dis. 2015; 15:1409–1419.

Macintyre F, Adoke Y, Tiono AB, Duong TT, Mombo-Ngoma G, Bouyou-Akotet M, et al. A randomised, double-blind clinical phase 2 trial of the efficacy, safety, tolerability and pharmacokinetics of a single dose combination treatment with artefenomel and piperaquine in adults and children with uncomplicated *Plasmodium falciparum* malaria. BMC Med. 2017; 15:181.
