# Supplementary material for: Randomized, open-label, phase 2a study to evaluate the contribution of artefenomel to the clinical and parasiticidal activity of artefenomel plus ferroquine in African patients with uncomplicated Plasmodium falciparum malaria
Source: Malar J. 2023 Jan 3;22:2. doi: 10.1186/s12936-022-04420-2 (PMC9809015; doi:10.1186/s12936-022-04420-2)
Supplement: Supplementary file 3 — Additional file 3: Pharmacokinetic analysis supporting the primary analysis. [file 12936_2022_4420_MOESM3_ESM.pdf]

Additional file 3: Pharmacokinetic analysis

Contents

Methods 2
Pharmacokinetic samples 2
Pharmacokinetic analysis 2
Historical population PK models 2
Historical population PK models validation 5
Results 6
Data 6
Ferroquine/desmethyl-ferroquine PK parameter estimation 13
Artefenomel PK parameter estimation 16
Ferroquine/desmethyl-ferroquine exposure parameters 18
Artefenomel exposure parameters 21
References 22

Abbreviations

$AUC_{[0\text{-}inf]}$, area under the concentration–time curve from 0 to infinity

$AUC_{[0\text{-}day28]}$, area under the concentration–time curve from 0 to day 28

BLOQ, below the limit of quantification

C_{max}, maximal observed concentration

C_{day7}, concentration at Day 7 post-dose

FALCI: ferroquine and artefenomel in adults and children with *Plasmodium falciparum* malaria

FQ, ferroquine

SSR or SSR97213, desmethyl-ferroquine

OZ or OZ439, artefenomel

PK, pharmacokinetic

Methods

Population pharmacokinetic techniques were applied using historical population PK models to estimate the individual patient pharmacokinetic (PK) parameters. The analysis was pre-specified in the PK analysis plan of the study.

All data processing, analysis, model setup and modeling result analysis were conducted within R (Microsoft Open R 3.5.1) combined with the IQR package (v1.1.1) developed by IntiQuan (IQR Tools, https://iqrtools.intiquan.com) to support the entire workflow of a PK/PD analysis from estimations to simulations. For all estimations, a nonlinear mixed effects (NLME) modeling approach was performed using the importance sampling algorithm of Monolix (Monolix version 2019R1. Antony, France: Lixoft SAS, 2019) by automatically generating and running MONOLIX projects through IQR from R environment. The log-likelihood and the Fisher information matrix were approximated by linearization. The individual parameters were determined as conditional modes.

Pharmacokinetic samples

Per the protocol, samples for PK analysis of artefenomel in plasma and ferroquine/SSR97213 in blood (dried blood spot) were collected. Samples for analysis of ferroquine and desmethyl-ferroquine concentration were collected pre-dose, and following dosing at 1, 4, 6, 8, 12, 24, 72, 168 hours, and on Day 14±1 and Day 28±2. Samples for analysis of artefenomel concentration were collected pre-dose, and at 1, 2, 4, 6, 12, 24, 48, 72, 120, 168 hours, and on Day 14±1 post-dose. All PK samples were processed and analyzed using validated methods by liquid chromatography tandem mass spectroscopy (LC-MS/MS). The lower limit of quantification (LLOQ) was 1 ng/mL for artefenomel and 5 ng/ml for ferroquine and desmethyl-ferroquine. Observations below the quantification limit were included in the data set. These data were handled with the M3 method as implemented in Monolix, (Samson 2006).

Pharmacokinetic analysis

The individual Empirical Bayes Estimates of the PK parameters for each individual patient and each analyte (artefenomel, ferroquine and desmethyl-ferroquine) were estimated applying the historical population PK models developed previously (described below). The historical structural population PK model was fitted to the observed data and dosing history of the study. All parameters were fixed and only the residual error was estimated to obtain the individual PK parameters for each subject as post-hoc estimates. The individual fits were evaluated visually to confirm that the individual plasma profiles were adequately described.

Subsequently, individual concentration time profiles were simulated using the estimated individual PK parameters and actual doses allowing estimation of the individual exposures, including the maximal observed concentration ($C_{\max}$), concentration at Day 7 post-dose (C_{day7}), $AUC_{[0\text{-}inf]}$ and $AUC_{[0\text{-}day28]}$ (only estimated for ferroquine/desmethyl-ferroquine).

All patients who vomited after either ferroquine or artefenomel were considered vomiters for the analyses and exposure statistics summaries unless they were successfully re-dosed (i.e., without vomiting after re-dose). If patients vomited after ferroquine, they were not to be re-dosed with ferroquine but received rescue medication. If patients vomited within 5 minutes after artefenomel administration, they were to be re-dosed with artefenomel only. No patients were re-dosed with artefenomel.

Historical population PK models

Separate historical population PK models for ferroquine (including its active metabolite desmethyl-ferroquine) and artefenomel were used to estimate individual PK parameters. Population PK parameter

estimates of ferroquine/desmethyl-ferroquine and artefenomel population PK models in patients are summarized in Table 1 and Table 2, respectively.

A single population PK model including both ferroquine and its active metabolite desmethyl-ferroquine was developed and validated, using the data of 541 patients from 7 phase 1 and phase 2 studies conducted in healthy volunteers, asymptomatic adult subjects and symptomatic adult and pediatric patients (>2 years old) (Boulu, 2016). It included a 3- and 2-compartment PK model for ferroquine and desmethyl-ferroquine, respectively. The absorption of ferroquine was described with a first-order absorption process, lag-time and relative bioavailability. Allometric scaling was implemented and disease status (i.e., healthy volunteers and asymptomatic subject versus symptomatic patients) was identified as a covariate. Some data from the FALCI study (reported in Boulu, 2016), where ferroquine was co-administered with artefenomel, were included in the model development. No effect of artefenomel on the PK of ferroquine or its metabolite was identified. For artefenomel, a population PK model was previously developed, based on 3 phase 2 studies: two monotherapy studies (MMV_OZ439_10_002 and MMV_OZ439_12_006) and one study in combination with piperaquine which included African and Asian men and women; age 6 months to 60 years; body weight range 5.6 to 89 kg; single doses 100 to 1200 mg; 800 mg when dosed with piperaquine (MMV_OZ439_13_003) (reported in Macintyre, 2017). It included a 3-compartment disposition model with first-order absorption and a lag-time. Body weight, vomiting, age, actual artefenomel dose and adult equivalent artefenomel dose were all identified as covariates. In none of the studies, artefenomel was co-administered with ferroquine.

Table 1 Parameter estimates for ferroquine historical population PK model in patients (Boulu, 2016)

**Parameter**		**Estimate[a]**	**BSV[b] (%)**
Ferroquine			
F	Relative Oral Bioavailability	0.873 (2)	124 (45)
$F_{withOZ439}$	Relative Oral Bioavailability when co-administered with OZ439	1	-
tlag (hr)	Absorption lag time	0	-
$tlag_{withOZ439}$ (hr)	Absorption lag time when co-administered with OZ439	0.45 (1)	-
ka (1/hr)	Absorption rate constant	0.71 (8)	110 (12)
Cl/F (L/hr)	Apparent Clearance	$13.5(6) * \left(\frac{BW}{65}\right)^{0.75}$	46 (10)
V1/F (L)	Apparent central volume of distribution	$2590(4) * \left(\frac{BW}{65}\right)^{1}$	56 (10)
Q/F (L/hr)	Apparent inter compartmental Clearance 1	$141(7) * \left(\frac{BW}{65}\right)^{0.75}$	90 (13)
V2/F (L)	Apparent peripheral volume of distribution 1	$3540(4) * \left(\frac{BW}{65}\right)^{1}$	37 (16)
Q2/F (L/hr)	Apparent inter compartmental Clearance 2	$19.5(8) * \left(\frac{BW}{65}\right)^{0.75}$	98 (11)

Parameter		Estimate[a]	BSV[b] (%)
V3/F (L)	Apparent peripheral volume of distribution 2	12400 (5)	-
Desmethyl-ferroquine			
Cl_{pm} (L/hr)	Transformation FQ to SSR	0.25 (4)	19 (18)
CL/F (L/hr)	Apparent Clearance	0.20 (4)	16 (26)
V1/F (L)	Apparent central volume of distribution	1 (fixed)	-
Q/F (L/hr)	Apparent inter compartmental Clearance 1	0.72 (6)	42 (12)
V2/F (L)	Apparent peripheral volume of distribution 1	43 (5)	47 (18)
residual	Proportional ferroquine	0.22 (2)	
residual	Proportional desmethyl-ferroquine	0.26 (2)	-

[a]Estimate, with between brackets the RSE (Relative Standard Error %)

[b]Between Subject Variability

BW= body weight (kg)

Table 2 Parameter estimates for artefenomel historical population PK model in patients (Macintyre, 2017)

Parameter		Estimate[a]	BSV[b] (%)
F	Relative Oral Bioavailability	$1 * \left(\frac{\mathrm{AGE}}{20}\right)^{0.19(12)}$	62 (4)
F_{vom}	Relative Oral Bioavailability in Vomiters	$0.51(9) * \left(\frac{\mathrm{AGE}}{20}\right)^{0.19(12)}$	86 (8)
tlag (hr)	Absorption lag time	0.41 (1)	14 (8)
ka (1/hr)	Absorption rate constant	$0.17(2) * \left(\frac{\mathrm{ODOS}}{800}\right)^{-0.34(8)}$	22 (8)
Cl/F (L/hr)	Apparent Clearance	$49.2(2) * \left(\frac{\mathrm{BW}}{50}\right)^{0.75} \left(\frac{\mathrm{ODGP}}{800}\right)^{-0.37(11)}$	33 (5)
V1/F (L)	Apparent central volume of distribution	$135(5) * \left(\frac{\mathrm{BW}}{50}\right)^{1}$	73 (6)
Q/F (L/hr)	Apparent inter compartmental Clearance 1	$9.7(4) * \left(\frac{\mathrm{BW}}{50}\right)^{0.75}$	36 (8)
V2/F (L)	Apparent peripheral volume of distribution 1	$269(5) * \left(\frac{\mathrm{BW}}{50}\right)^{1}$	-

Parameter		Estimate[a]	BSV[b] (%)
Q2/F (L/hr)	Apparent inter compartmental Clearance 2	$7.0(3) * \left(\frac{\mathrm{BW}}{50}\right)^{0.75}$	-
V3/F (L)	Apparent peripheral volume of distribution 2	$1130(4) * \left(\frac{\mathrm{BW}}{50}\right)^{1}$	51 (2)
residual	proportional	0.26 (2)	-

[a]Estimate, with between brackets the RSE (Relative Standard Error %)

[b]Between Subject Variability

AGE=age (years); ODOS=actual administered dose (mg); ODGP=adult equivalent dose (mg); BW= body weight (kg)

Historical population PK models validation

The assumption that historical population PK models would reasonably describe the PK of both drugs in this study population was evaluated through visual predictive check (VPC) plots, stratified by artefenomel dose. For all three analytes, concentration observations (uncertainty, between subject variability and residual error) were simulated for the dosing and population of the current study (covariates and number of patients) 100 times using the historical population PK models and parameters. The predicted concentrations were summarized and plotted with the actual observations.

Results

Data

The PK analysis population for artefenomel consisted of 105 patients. The PK Population for ferroquine consisted of 140 patients who were dosed with ferroquine. The data sets used in the analysis included a total of 1303 samples for artefenomel and 1524 samples for ferroquine/desmethyl-ferroquine.

For the ferroquine/desmethyl-ferroquine analysis 2 samples removed from the analysis because of sample date-times missing, and 2 samples because of a positive pre-dose ferroquine and desmethyl-ferroquine concentration. For the artefenomel analysis, 4 samples were removed because of a positive artefenomel concentration. Data exploration revealed 12 patients with at least 90% of PK observations measured below the limit of quantitation (BLOQ) for at least one drug and these patients were flagged due to the associated uncertainty of PK estimates and derived exposure metrics. A post-hoc sensitivity analysis verified that these patients did not influence the conclusions of the exposure-response analyses. The ferroquine and artefenomel median observed concentration over time profiles are presented in Figure 1.

Figure 1 Artefenomel and ferroquine median observed concentration over time

A. Artefenomel. B. Ferroquine. Continuous lines represent the median PK profile.

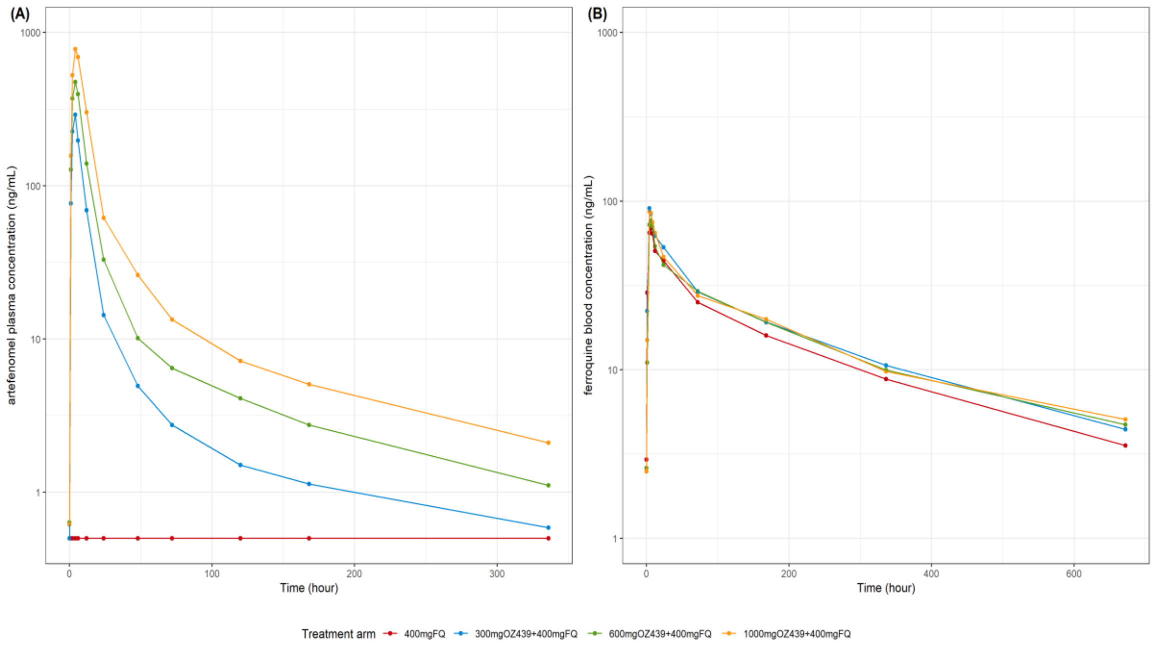

Visual Predictive Check (VPC) plots

Median observed ferroquine and desmethyl-ferroquine blood concentrations were slightly lower than predicted concentrations, although overall, historical models for ferroquine/desmethyl-ferroquine and artefenomel adequately described the data from the present study, as shown by VPC plots in Figures 2, 3 and 4, respectively.

Figure 2 VPC of ferroquine observations and ferroquine/desmethyl-ferroquine model predictions stratified by dose

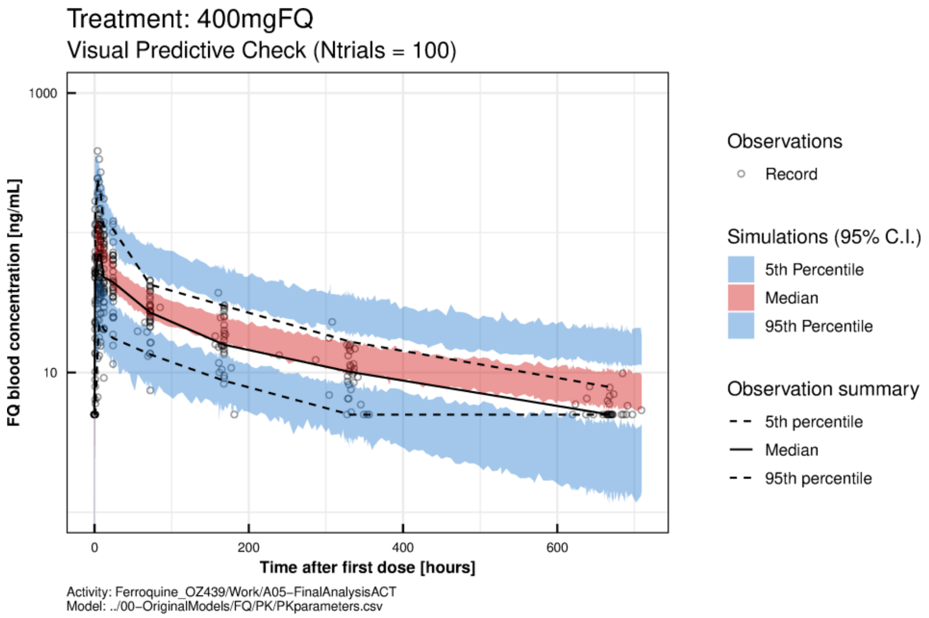

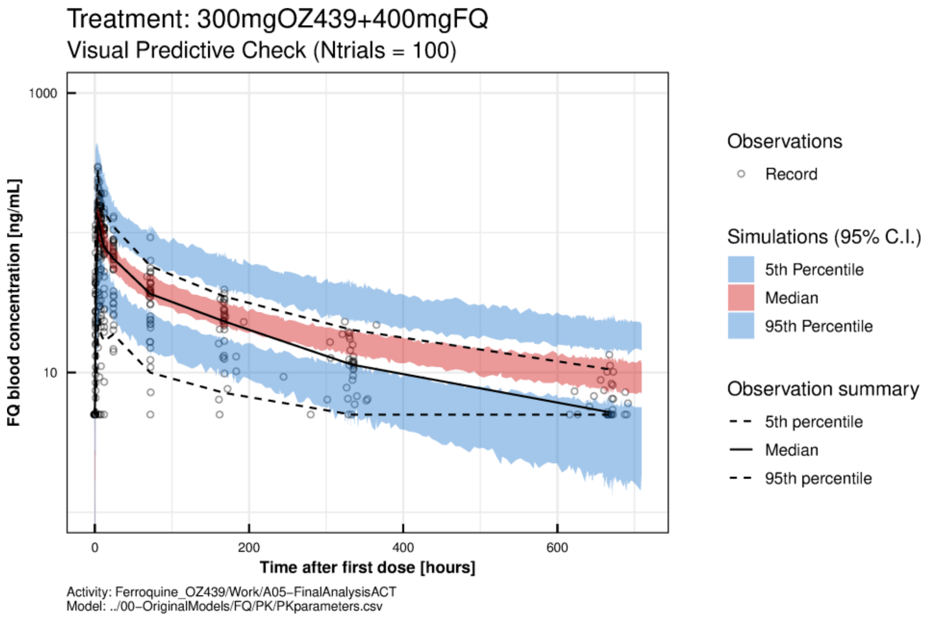
Treatment: 300mgOZ439+400mgFQ
Visual Predictive Check (Ntrials = 100)
FQ blood concentration [ng/mL]
1000
10
0
200
400
600
Time after first dose [hours]
Observations
Record
Simulations (95% C.I.)
5th Percentile
Median
95th Percentile
Observation summary
5th percentile
Median
95th percentile
Activity: Ferroquine_OZ439/Work/A05–FinalAnalysisACT
Model: ../00–OriginalModels/FQ/PK/PKparameters.csv

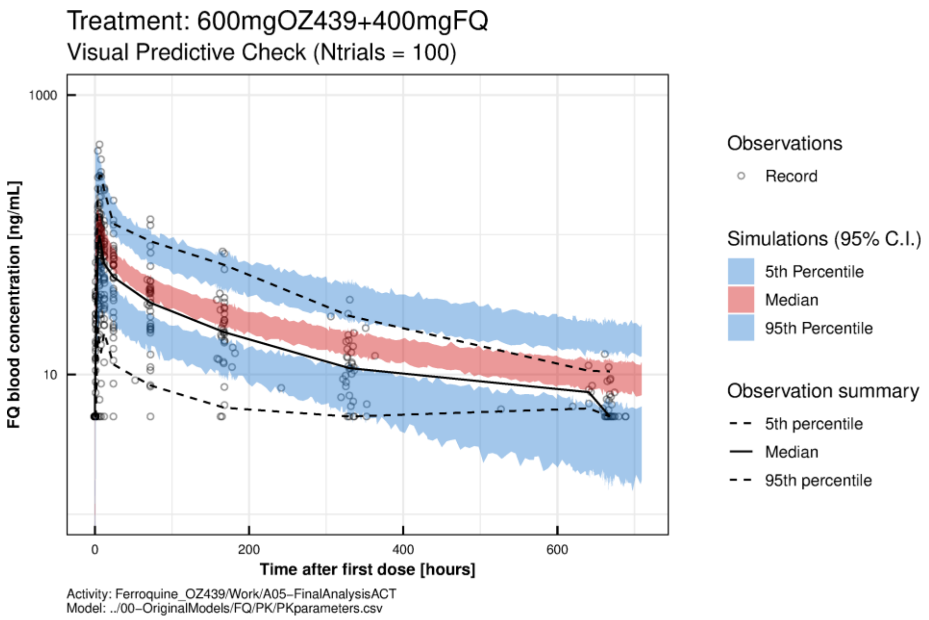
Treatment: 600mgOZ439+400mgFQ
Visual Predictive Check (Ntrials = 100)
FQ blood concentration [ng/mL]
1000
10
0
200
400
600
Time after first dose [hours]
Observations
Record
Simulations (95% C.I.)
5th Percentile
Median
95th Percentile
Observation summary
5th percentile
Median
95th percentile
Activity: Ferroquine_OZ439/Work/A05–FinalAnalysisACT
Model: ../00–OriginalModels/FQ/PK/PKparameters.csv

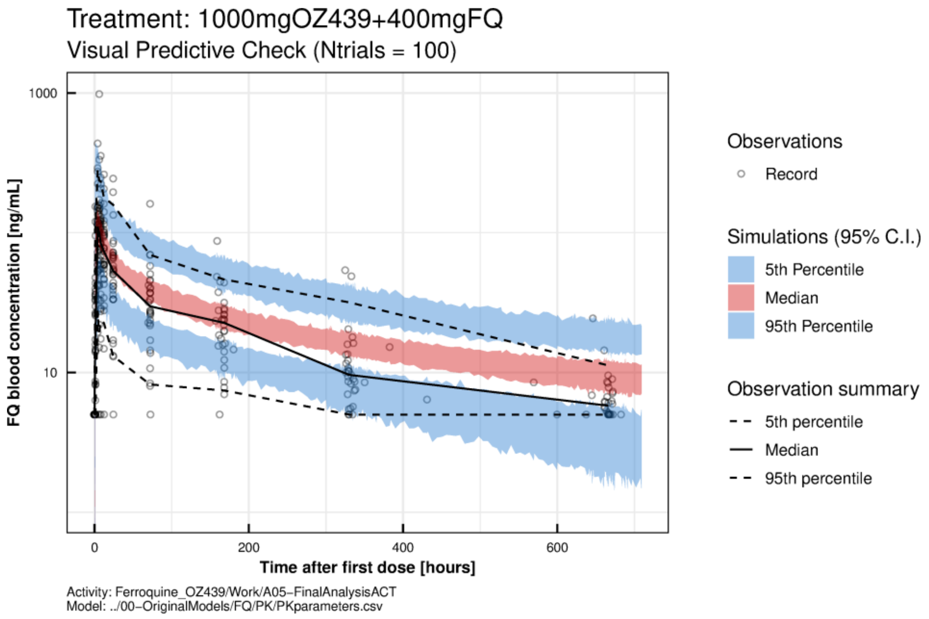

Treatment: 1000mgOZ439+400mgFQ
Visual Predictive Check (Ntrials = 100)
1000
10
FQ blood concentration [ng/mL]
0
200
400
600
Time after first dose [hours]
Activity: Ferroquine_OZ439/Work/A05–FinalAnalysisACT
Model: ../00–OriginalModels/FQ/PK/PKparameters.csv
Observations
Record
Simulations (95% C.I.)
5th Percentile
Median
95th Percentile
Observation summary
5th percentile
Median
95th percentile

Figure 3 VPC of desmethyl-ferroquine observations and ferroquine/desmethyl-ferroquine model predictions stratified by dose

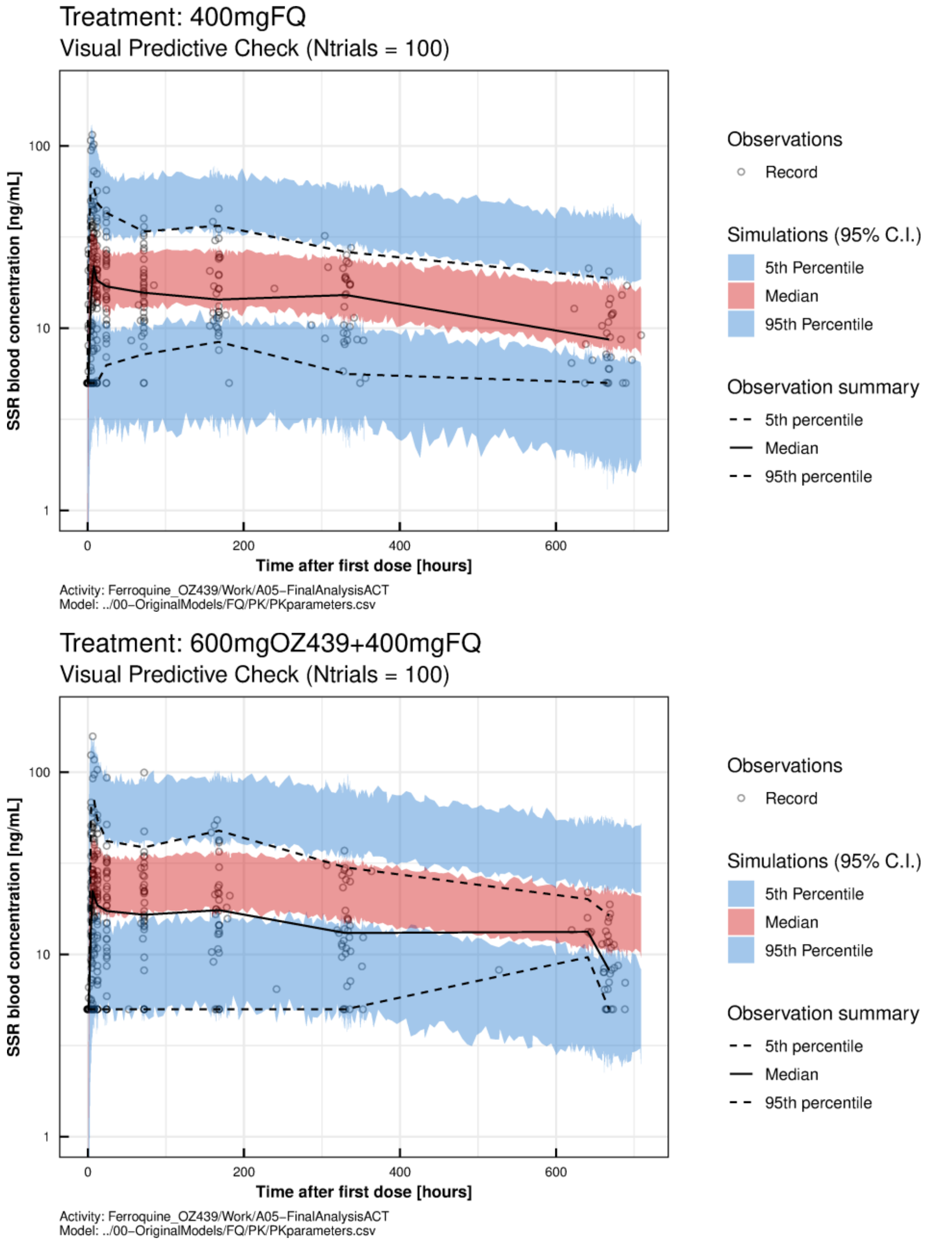

Treatment: 300mgOZ439+400mgFQ

Visual Predictive Check (Ntrials = 100)

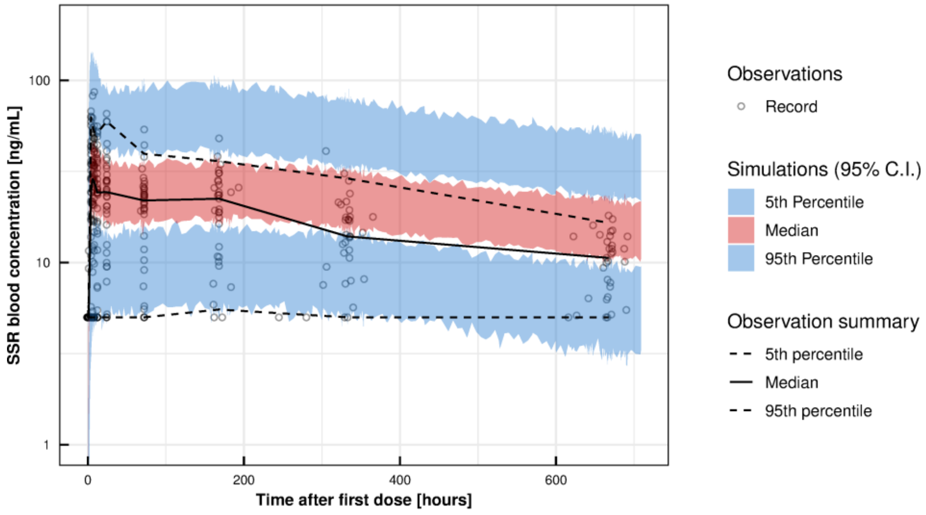

Activity: Ferroquine_OZ439/Work/A05–FinalAnalysisACT
Model: ../00–OriginalModels/FQ/PK/PKparameters.csv

Treatment: 1000mgOZ439+400mgFQ

Visual Predictive Check (Ntrials = 100)

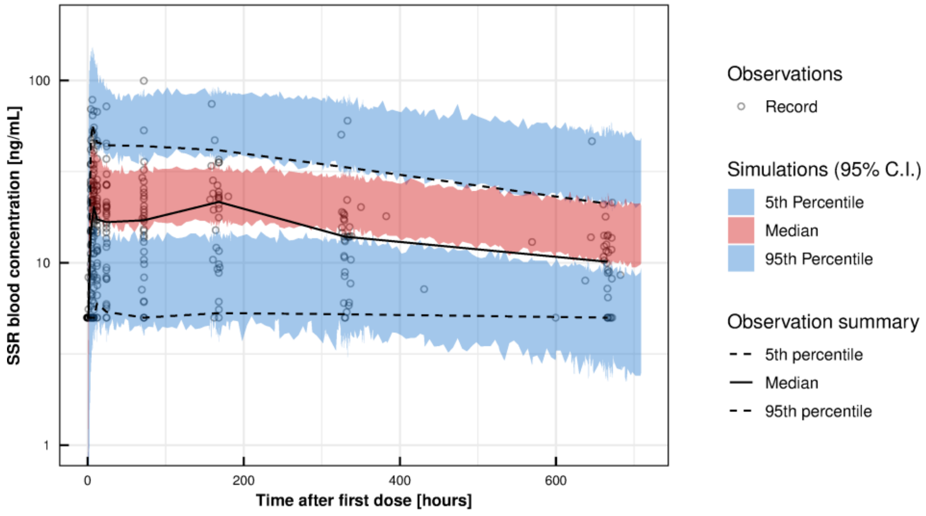

Activity: Ferroquine_OZ439/Work/A05–FinalAnalysisACT
Model: ../00–OriginalModels/FQ/PK/PKparameters.csv

Figure 4 VPC of artefenomel observations and artefenomel model predictions stratified by dose

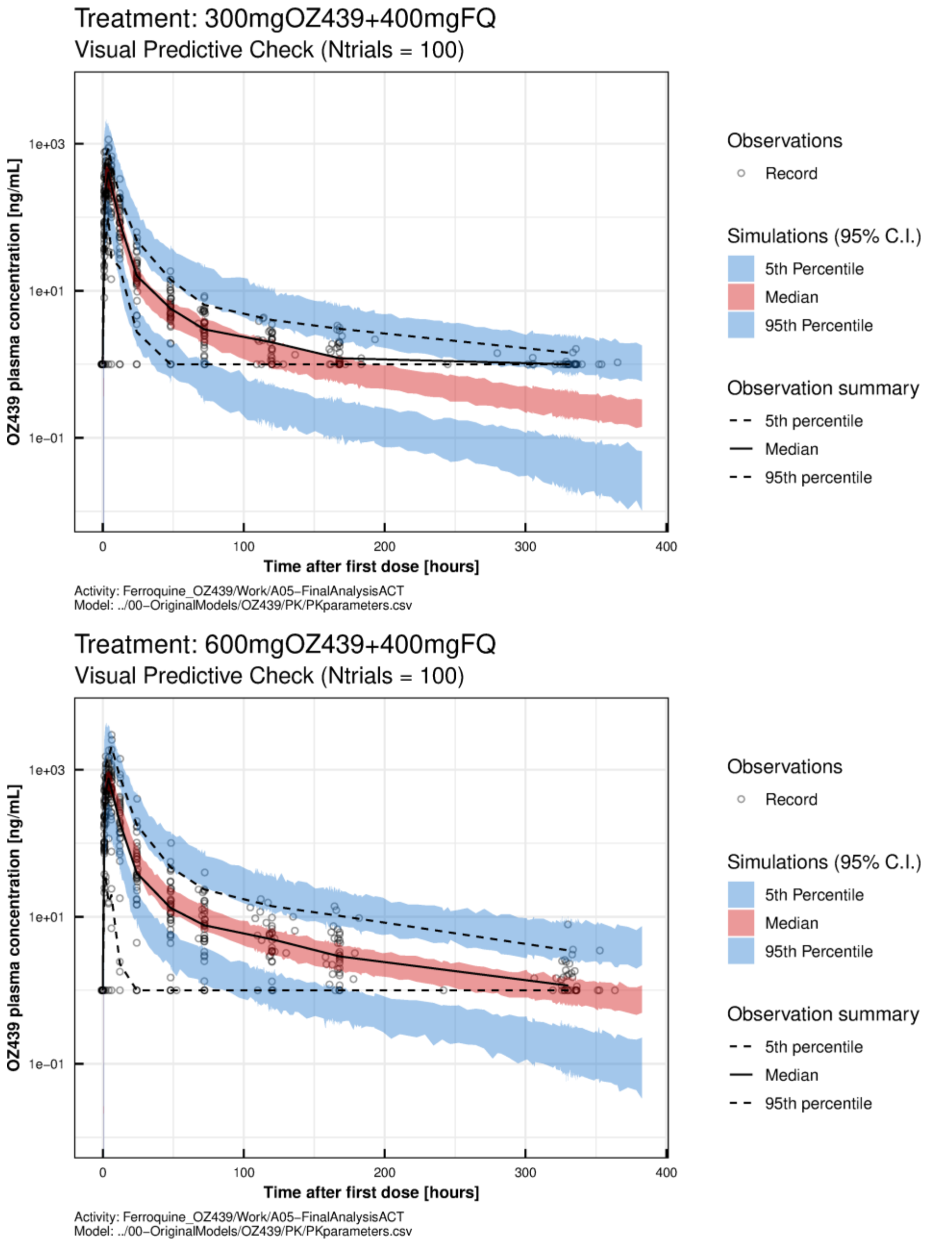

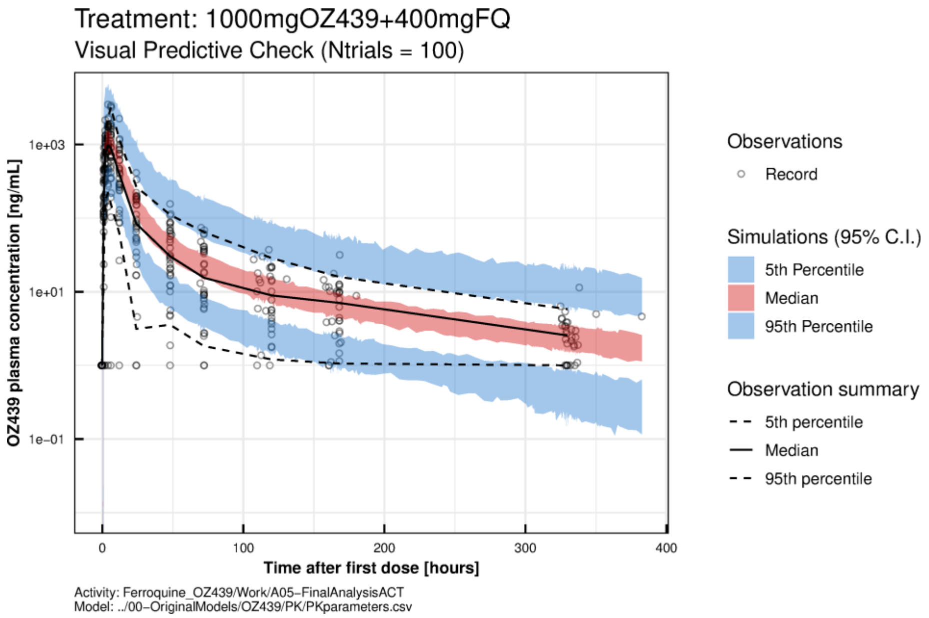

Ferroquine/desmethyl-ferroquine PK parameter estimation

Individual Empirical Bayes Estimates of ferroquine and desmethyl-ferroquine PK parameters were estimated for all patients in the PK population (N=140). Individual concentration over time data was well described for most patients (see Figure 5). Goodness of fit plots showed a good distribution around the line of unity and no trend was observed in residuals plots.

Figure 5 Example of individual ferroquine and desmethyl-ferroquine PK profile and model fit

Ferroquine:

Individual fits

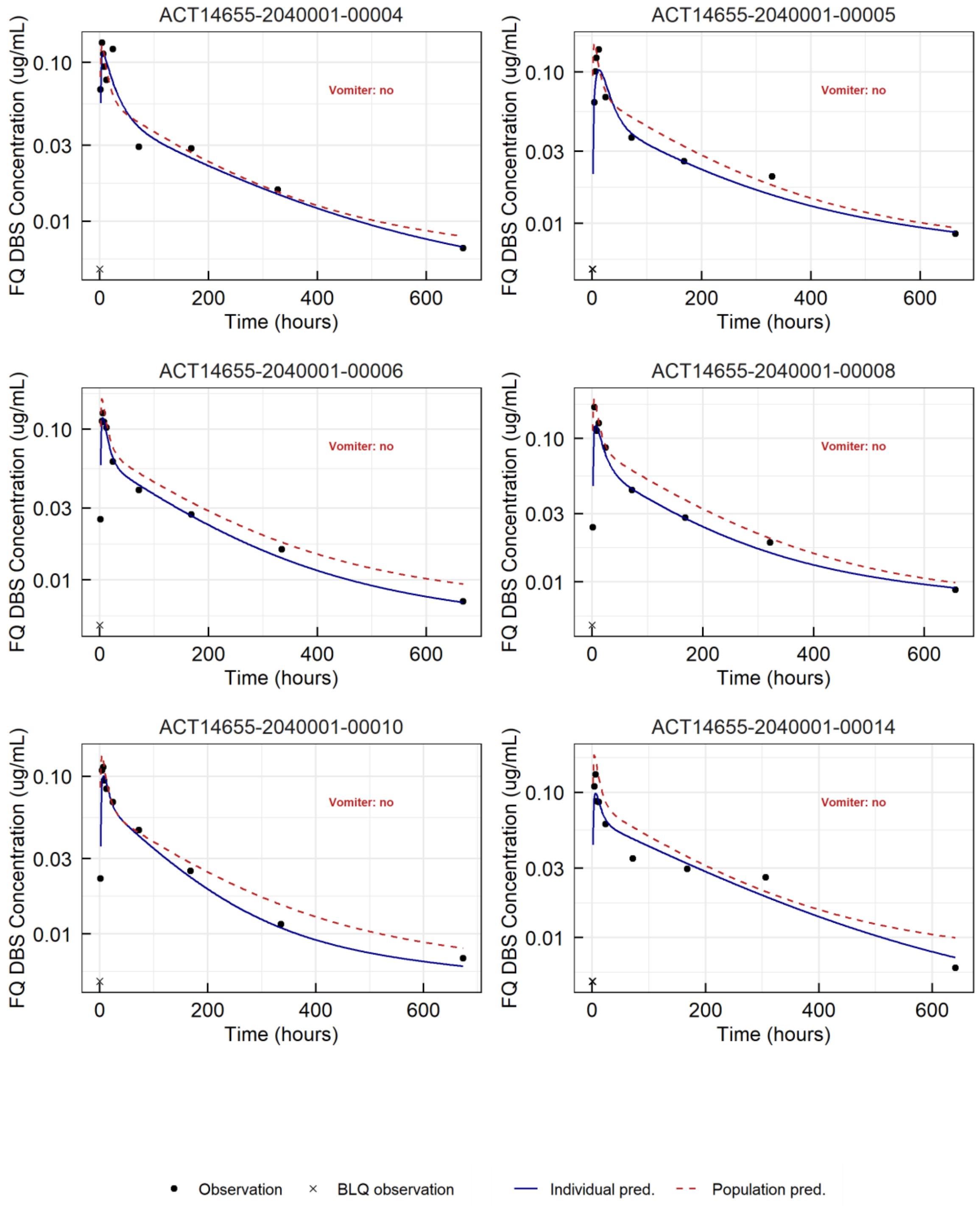

Desmethyl-ferroquine:

Individual fits

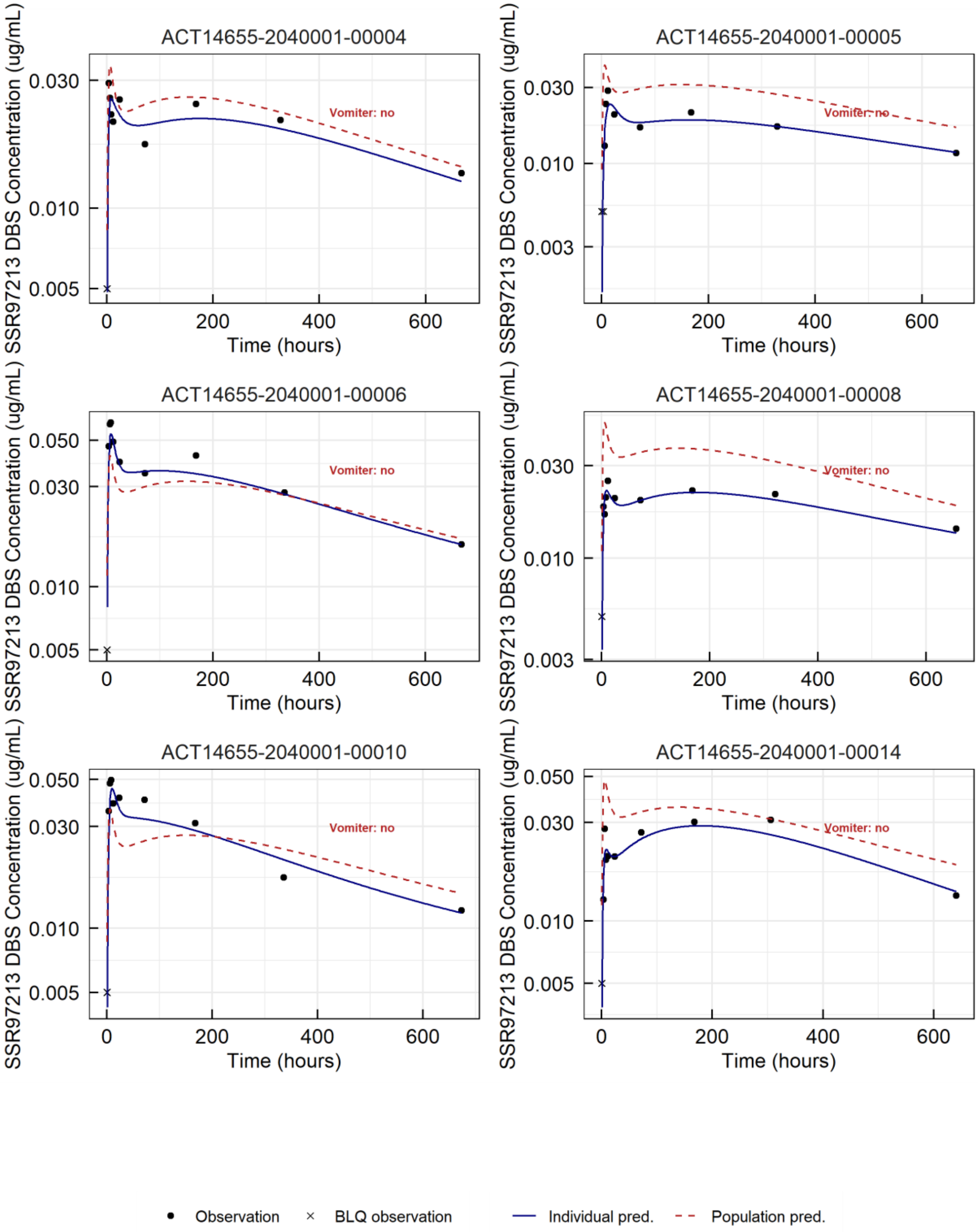

Artefenomel PK parameter estimation

Individual Empirical Bayes Estimates of artefenomal PK parameters were estimated for all patients in the PK population (N=105 patients dosed with artefenomel from a total of 140). Individual concentration over time data was well described for most patients. Goodness of fit plots showed a good distribution around the line of unity and no trend was observed in residuals plots.

Data from patients flagged due to at least 90% of observations BLOQ was adequately described; except for one patient (ACT14655−2660001−00006) where the estimated C_{max} was heavily penalized by the fact that the patient received the highest dose of artefenomel (1000 mg) and did not vomit but all observations were BLOQ (see Figure 6).

Figure 6 Example of individual artefenomel PK profile and model fit

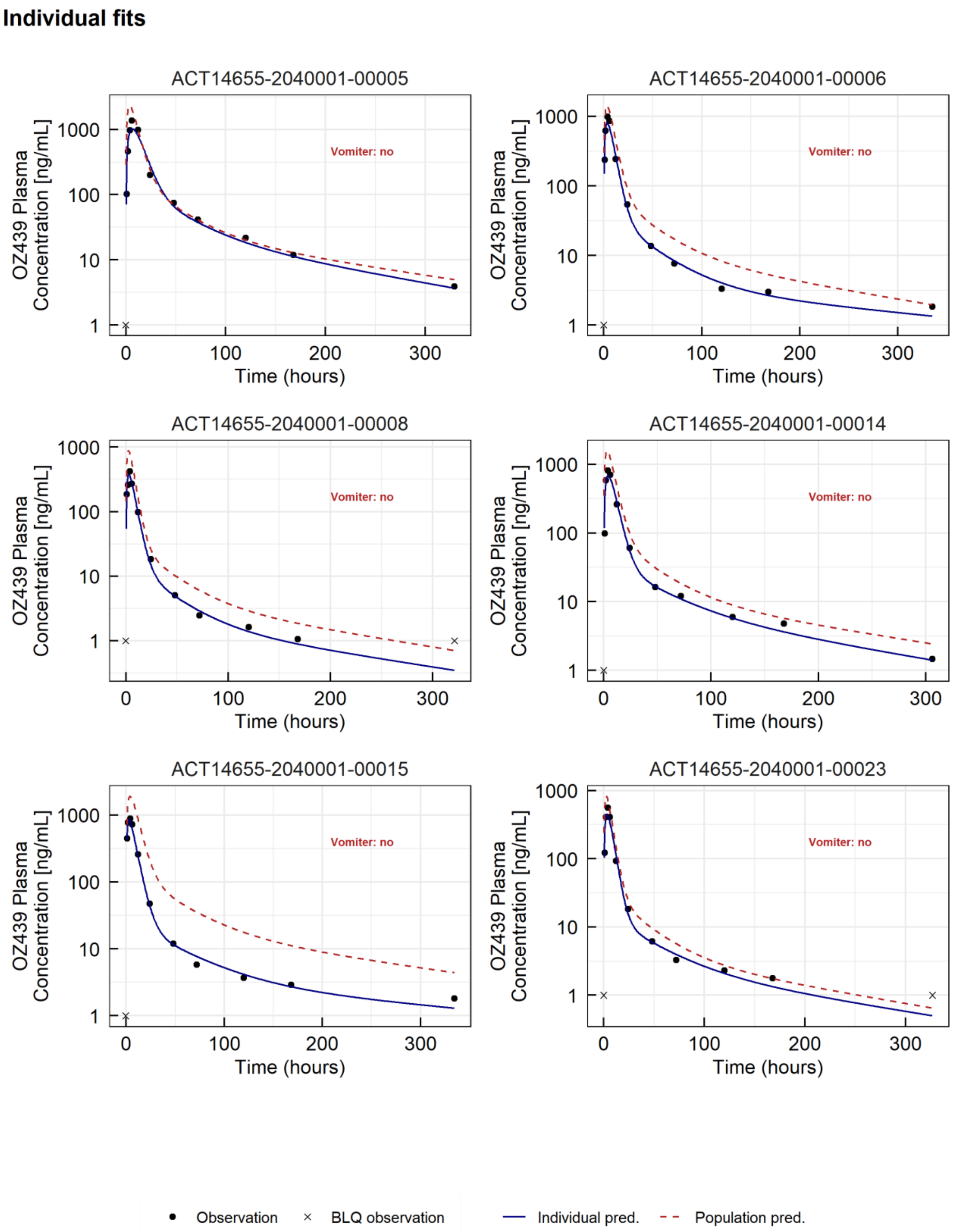

Ferroquine/desmethyl-ferroquine exposure parameters

The mean exposures of ferroquine and desmethyl-ferroquine were highly correlated and comparable across treatment groups (see Table 3 and Table 4).

The overall between-patient variability was moderate to high. Only one patient vomited in the ferroquine 400 mg treatment group and this patient had similar exposures to non-vomiters in the same treatment group. In treatment groups dosed with artefenomel, patients who vomited had lower ferroquine exposures (40% average decrease in C_{day7} and $AUC_{[0\text{-}day28]}$) and higher between-patient variability was observed compared to non-vomiters.

Selected ferroquine and desmethyl-ferroquine exposure parameters are summarized in Table 3 and Table 4, respectively, both overall and by vomiting status for the PK/PD efficacy population (n=132).

Table 3 Summary statistics of ferroquine exposures by vomit status in the PK/PD efficacy population

Treatment Arm Artefenomel (mg): Ferroquine (mg)	Subgroup	n[a]	C_{max}[b] [ng/mL]	C_{day7}[b] [ng/mL]	$AUC_{[0-day28]}$[b] [ug*hr/mL]	$AUC_{[0-inf]}$[b] [µg*hr/mL]
0: 400	All patients	31	80.99 (61%)	15.86 (44%)	8.874 (42%)	15.5 (47%)
300: 400	All patients	33	79.95 (93%)	18.32 (42%)	10.33 (40%)	17.81 (43%)
600: 400	All patients	36	72.64 (88%)	18.69 (52%)	10.13 (51%)	16.3 (63%)
1000: 400	All patients	32	82.45 (95%)	17.83 (60%)	10.14 (56%)	16.87 (59%)
**Vomiting Status**						
0: 400	Non-Vomiters	30	81.08 (62%)	15.83 (45%)	8.869 (42%)	15.41 (48%)
	Vomiters	1	78.37 (NA%)	16.67 (NA%)	9.037 (NA%)	18.26 (NA%)
300: 400	Non-Vomiters	30	87.82 (80%)	19.15 (39%)	10.79 (38%)	18.22 (43%)
	Vomiters	3	31.28 (154%)	11.76 (52%)	6.652 (43%)	14.2 (49%)
600: 400	Non-Vomiters	30	86.7 (59%)	20.79 (44%)	11.26 (41%)	18.33 (50%)
	Vomiters	6	30 (150%)	10.98 (51%)	5.951 (60%)	9.081 (85%)
1000: 400	Non-Vomiters	23	101.3 (55%)	20.15 (49%)	11.41 (49%)	18.46 (60%)
	Vomiters	9	48.67 (164%)	13.03 (76%)	7.501 (64%)	13.41 (48%)

The reported concentrations are blood concentrations.

[a] Number of patients with determinable metric; [b] Geometric Mean (CV%).

Table 4 Summary statistics of desmethyl-ferroquine exposures by vomit status in the PK/PD efficacy population

Treatment Arm Artefenomel (mg): Ferroquine (mg)	Subgroup	n[a]	C_{max}[b] [ng/mL]	C_{day7}[b] [ng/mL]	$AUC_{[0-day28]}$[b] [ug*hr/mL]	$AUC_{[0-inf]}$[b] [µg*hr/mL]
0: 400	All patients	31	24.58 (61%)	16.15 (51%)	8.878 (48%)	19.58 (53%)
300: 400	All patients	33	22.65 (83%)	16.03 (68%)	9.051 (56%)	20.09 (48%)
600: 400	All patients	36	19.14 (99%)	15.2 (78%)	8.483 (68%)	17.94 (69%)
1000: 400	All patients	32	19.76 (78%)	14.95 (86%)	8.487 (71%)	18.32 (68%)
**Vomiting Status**						
0: 400	Non-Vomiters	30	24.76 (62%)	16.13 (52%)	8.856 (48%)	19.4 (53%)
	Vomiters	1	19.81 (NA%)	16.82 (NA%)	9.579 (NA%)	25.56 (NA%)
300: 400	Non-Vomiters	30	24.99 (72%)	17.36 (58%)	9.677 (49%)	20.8 (46%)
	Vomiters	3	8.465 (107%)	7.259 (105%)	4.636 (78%)	14.18 (58%)
600: 400	Non-Vomiters	30	23.33 (78%)	18 (59%)	9.847 (53%)	20.68 (52%)
	Vomiters	6	7.127 (91%)	6.53 (83%)	4.027 (74%)	8.817 (90%)
1000: 400	Non-Vomiters	23	23 (69%)	17.36 (66%)	9.632 (62%)	20.03 (70%)
	Vomiters	9	13.39 (83%)	10.19 (122%)	6.141 (84%)	14.6 (57%)

The reported concentrations are blood concentrations.

[a] Number of patients with determinable metric; [b] Geometric Mean (CV%).

Figure 7 shows the distribution of desmethyl-ferroquine $AUC_{[0-day28]}$ for the PKPD Efficacy population.

Figure 7 Distribution of desmethyl-ferroquine $AUC_{[0-day28]}$ for the PKPD Efficacy population

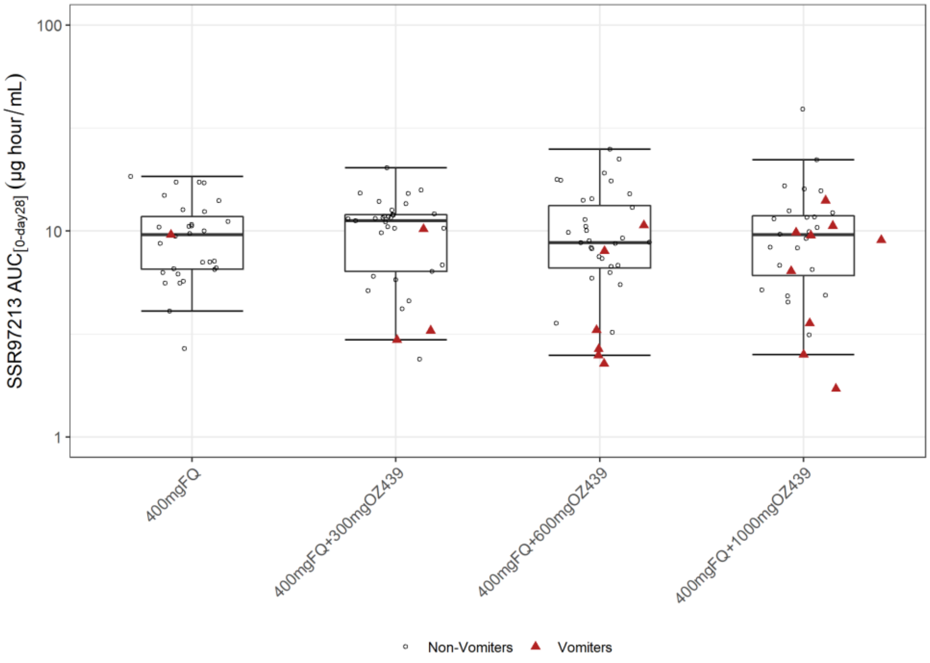

Figure 8 shows the correlation between ferroquine and desmethyl-ferroquine $AUC_{[0\text{-}day28]}$.

Figure 8 Relationship between Ferroquine and desmethyl-ferroquine $AUC_{[0\text{-}day28]}$

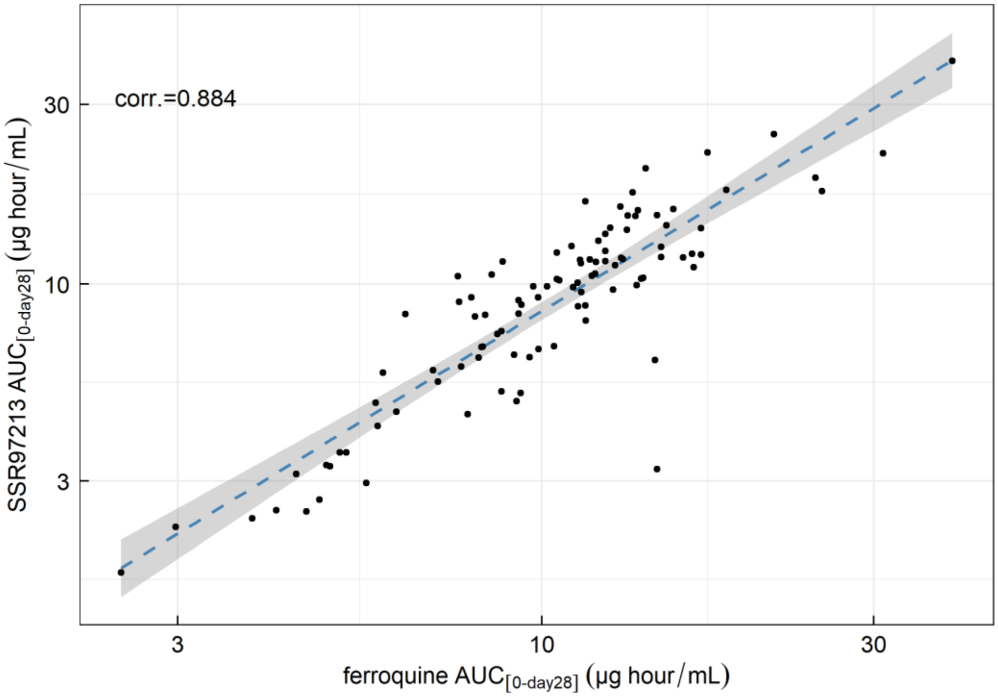

Correlation coefficient calculated for log-transformed values.
Dashed blue line and shaded area indicates linear regression model.

Artefenomel exposure parameters

Artefenomel exposures were approximately dose proportional (see Table 5). The overall between-patient variability in artefenomel exposures was very large and on average, patients who vomited presented lower artefenomel exposures (72% and 64% average decrease in C_{day7} and $AUC_{[0inf],}$ respectively) and a much higher between-patient variability was observed compared to non-vomiters.

Selected artefenomel exposure parameters are summarized in Table 5, both overall and by vomiting status for the PK/PD efficacy population (n=101 patients dosed with artefenomel from a total of 132).

Table 5 Summary statistics of artefenomel exposures by vomit status in the PK/PD efficacy population

Treatment Arm Artefenomel (mg): Ferroquine (mg)	Subgroup	n[a]	C_{max}[b] [ng/mL]	C_{day7}[b] [ng/mL]	$AUC_{[0-day28]}$[b] [µg*hr/mL]
300: 400	All patients	33	277.3 (169%)	0.9251 (110%)	3.269 (113%)
600: 400	All patients	36	488.3 (232%)	2.152 (202%)	6.46 (181%)
1000: 400	All patients	32	920.6 (77%)	4.445 (165%)	13.05 (114%)
**Vomiting Status**					
300: 400	Non-Vomiters	30	298.8 (169%)	1.069 (76%)	3.64 (94%)
	Vomiters	3	131.2 (143%)	0.2178 (363%)	1.114 (253%)
600: 400	Non-Vomiters	30	606.5 (204%)	3.117 (77%)	8.837 (102%)
	Vomiters	6	165.3 (220%)	0.3374 (649%)	1.349 (334%)
1000: 400	Non-Vomiters	23	1060 (56%)	5.662 (132%)	16.04 (87%)
	Vomiters	9	641.7 (115%)	2.395 (213%)	7.707 (156%)

The reported concentrations are plasma concentrations.

[a] Number of patients with determinable metric; [b] Geometric Mean (CV%).

References

Boulu L. POH0456. Population PK analysis of ferroquine (SSR97193), and its metabolite SSR97213 from a pool of phase I and II studies (TDU5419, TDU5967, TDR5969, INT6856, ACT10420, DRI10382, TDU12511 and DRI12805). Sanofi. Internal Report, 2016.

Macintyre F, Adoke Y, Tiono AB, Duong TT, Mombo-Ngoma G, Bouyou-Akotet M, et al. A randomised, double-blind clinical phase 2 trial of the efficacy, safety, tolerability and pharmacokinetics of a single dose combination treatment with artefenomel and piperaquine in adults and children with uncomplicated *Plasmodium falciparum* malaria. BMC Med. 2017; 15:181.

Modeling & Simulation in R – Supporting efficient model informed drug development with IQR tools. IntiQuan GmbH, Basel, Switzerland, 22 October 2020. https://iqrtools.intiquan.com/. Accessed 16 February 2021.

Monolix version 2019R1. Antony, France: Lixoft SAS, 2019. http://lixoft.com/products/monolix/. Accessed 16 February 2021.

Samson A, Lavielle M, Mentré F. Extension of the SAEM algorithm to left-censored data in nonlinear mixed-effects model: Application to HIV dynamics model. Computational Statistics & Data Analysis. 2006; 51(3):1562–1574.
