# Supplementary material for: Randomized, open-label, phase 2a study to evaluate the contribution of artefenomel to the clinical and parasiticidal activity of artefenomel plus ferroquine in African patients with uncomplicated Plasmodium falciparum malaria
Source: Malar J. 2023 Jan 3;22:2. doi: 10.1186/s12936-022-04420-2 (PMC9809015; doi:10.1186/s12936-022-04420-2)
Supplement: Supplementary file 4 — Additional file 4: Exposure–response pharmacokinetic/pharmacodynamic analysis. [file 12936_2022_4420_MOESM4_ESM.pdf]

Additional file 4: Exposure–response analysis

Contents

Methods 2
Logistic regression 2
Exposure–response for Day 28 PCR-adjusted ACPR 4
Exposure–response for Day 28 crude ACPR 6
References 8

Abbreviations

AIC, Akaike Information Criterion

$AUC_{[0\text{-}inf]}$, area under the concentration–time curve from 0 to infinity

$AUC_{[0\text{-}day28]}$, area under the concentration–time curve from 0 to day 28

ACPR, adequate clinical and parasitological response

C_{day7}, concentration at Day 7 post-dose

CI, confidence interval

FQ, ferroquine

PCR, polymerase chain reaction

SSR or SSR97213, desmethyl-ferroquine

OZ439, artefenomel

PD, pharmacodynamic

PK, pharmacokinetic

Methods

Logistic regression

The relationship between estimated artefenomel and ferroquine AUC and Day 28 PCR-adjusted adequate clinical and parasitological response (ACPR) and Day 28 crude ACPR (success or failure) was evaluated statistically by logistic regression. This relationship was suggested by the exploratory plots.

See [Figure 3] in the main paper for the relationship between artefenomel and ferroquine Day 28 PCR-adjusted ACPR. Figure 1 shows the plot for Day 28 crude ACPR.

Figure 1 Relationship between artefenomel and ferroquine AUC with Day 28 crude ACPR

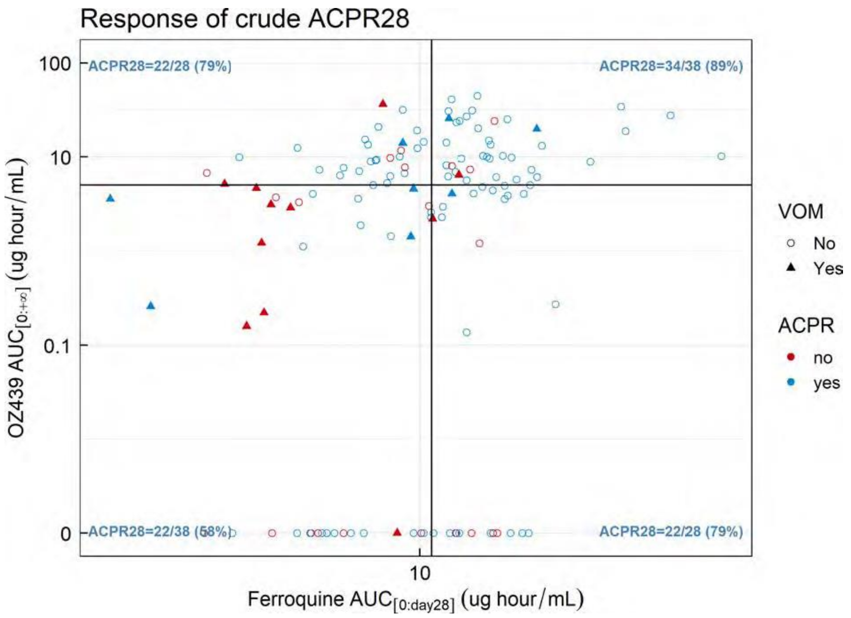

All data processing, analysis, model setup and modeling result analysis were conducted within R (Microsoft Open R 3.5.1) combined with the IQR package (v1.1.1) developed by IntiQuan (IQR Tools, https://iqrtools.intiquan.com) to support the entire workflow of a PK/PD analysis from estimations to simulations.

Baseline parasitemia, age, baseline body weight, sex, vomit status and study center were evaluated as covariates in the analysis. Data exploration suggested that 3 study centers (2 in Gabon and 1 in Benin) demonstrated lower efficacy (Day 28 PCR-adjusted ACPR ≤75%) compared to all other centers (Day 28 PCR-adjusted ACPR ≥85%). No obvious explanation for this observation was identified. The covariate "Low Efficacy Study Center" was created by grouping the 3 centers in question versus all other centers to identify any study center effects. Baseline age and body weight were included in the logistic regression with units of 10 years and 10 kg respectively to allow for a better interpretation of the odds ratio (OR).

The general form of the logistic regression model can be presented as follows,

$$log(odds) = \beta_0 + \sum_{i=1}^{n} \beta_i * cov_i + \sum_{i=1}^{n-1} \sum_{j=i+1}^{n} \beta_{i,j} * cov_i * cov_j$$

where

$$log(odds) = logit(p) = log\left(\frac{p}{1-p}\right)$$

and where p is the probability of $ACPR_{28}$ response, β_0 is the intercept, β_i represents the slope for the effect of covariate (cov) i, and $\beta_{i,j}$ is the coefficient for the interaction between two covariates i and j.

For the logistic regression the function IQRlogisticRegression() in IQRtools was used which is an interface to the glm() function in R:

e.g. glm($ACPR_{28}$ ~ cov_1 + cov_2, family = binomial(link = "logit"), data=data)

The OR for each covariate was subsequently calculated by taking the exponent of the estimated slope: e.g. OR for COV1 = $e^{(\beta_1)}$. The Wald test was used to calculate the 95% CI of the OR.

The OR for a particular covariate can be interpreted as follows:

For a categorical variable: the OR represents the fold change in odds versus the reference

For a continuous variable: the OR represents the fold change in odds per unit of the variable

The model building process is summarized below.

A base model was developed first, including the ferroquine $AUC_{[0\text{-}day28]}$ as the basis for evaluation of any other covariate effects.

The remaining covariates were evaluated as follows:

A univariate analysis (compared to the base model) of each of the other covariates to be evaluated. Identification of any statistically significant (p-value <0.05 and >2 point lower Akaike Information Criterion [AIC]) covariates as well as covariates deemed scientifically credible or of particular interest.

All identified covariates were included in a new full model. Removal of each covariate was evaluated in a backward elimination analysis. If removal of one or more covariates did not make the model significantly worse (increase in AIC) the one covariate whose removal was associated with the lowest AIC was removed. This process was repeated until no covariate could be removed without resulting in a significantly worse (increase in AIC) model.

Evaluation of any interactions between the remaining covariates in the model. AIC and other statistical criteria as well as scientific judgement were applied.

A visual predictive check for the final model was performed by plotting the observed and model predicted Day 28 ACPR by treatment group for the study population with 90%CI in various subgroups. The simulations were based on the actual patient population in each subgroup (i.e., estimated individual $AUC_{[0\text{-}day28]}$ and covariates in those patients) and summarized across 1000 replicates.

Exposure–response for Day 28 PCR-adjusted ACPR

The relationship between the estimated exposure of artefenomel and ferroquine and the response defined as Day 28 PCR-adjusted ACPR was evaluated using logistic regression as described above. The following exposure variables were evaluated: artefenomel $AUC_{[0\text{-}inf]}$, ferroquine $AUC_{[0\text{-}day28]}$ and desmeythl-ferroquine $AUC_{[0\text{-}day28]}$. The contribution of artefenomel exposure was also evaluated as a binary variable by testing artefenomel treatment versus no artefenomel and by testing artefenomel $AUC_{[0\text{-}inf]}$ first quartile (Q1, corresponding to lower artefenomel exposure) versus the remaining quantiles (combined Q2, Q3 and Q4, corresponding to higher artefenomel exposure).

Univariate analysis for Day 28 PCR-adjusted ACPR is shown in Figure 2.

Figure 2 Univariate analysis for Day 28 PCR-adjusted ACPR

Factor	Odds Ratio [95% CI]	P-value
**Base Model (BM):**		
**FQ $AUC_{[0\text{-}day28]}$** ug*hour/mL	1.2 [1.1, 1.5]	0.0161
**BM + Univariate Addition:**		
**SSR97213 $AUC_{[0\text{-}day28]}$** ug*hour/mL	1.2 [0.95, 1.6]	0.113
**OZ439 $AUC_{[0\text{-}inf]}$** ug*hour/mL	1.1 [0.98, 1.2]	0.207
**OZ439 treatment** Yes vs No	1.7 [0.47, 5.6]	0.384
**OZ439 $AUC_{[0\text{-}inf]}$** Q1 vs Q2-Q4	2.1 [0.62, 6.7]	0.212
**Baseline Parasitemia** log10 (p/mL)	0.1 [0.012, 0.64]	0.0235
**Age** 10 years	1.6 [0.89, 3.8]	0.192
**Baseline Bodyweight** 10 kg	1.5 [0.88, 2.6]	0.158
**Sex** Male vs Female	0.32 [0.1, 1]	0.0524
**Low Efficacy Study Center** Yes vs No	0.11 [0.027, 0.36]	<0.001

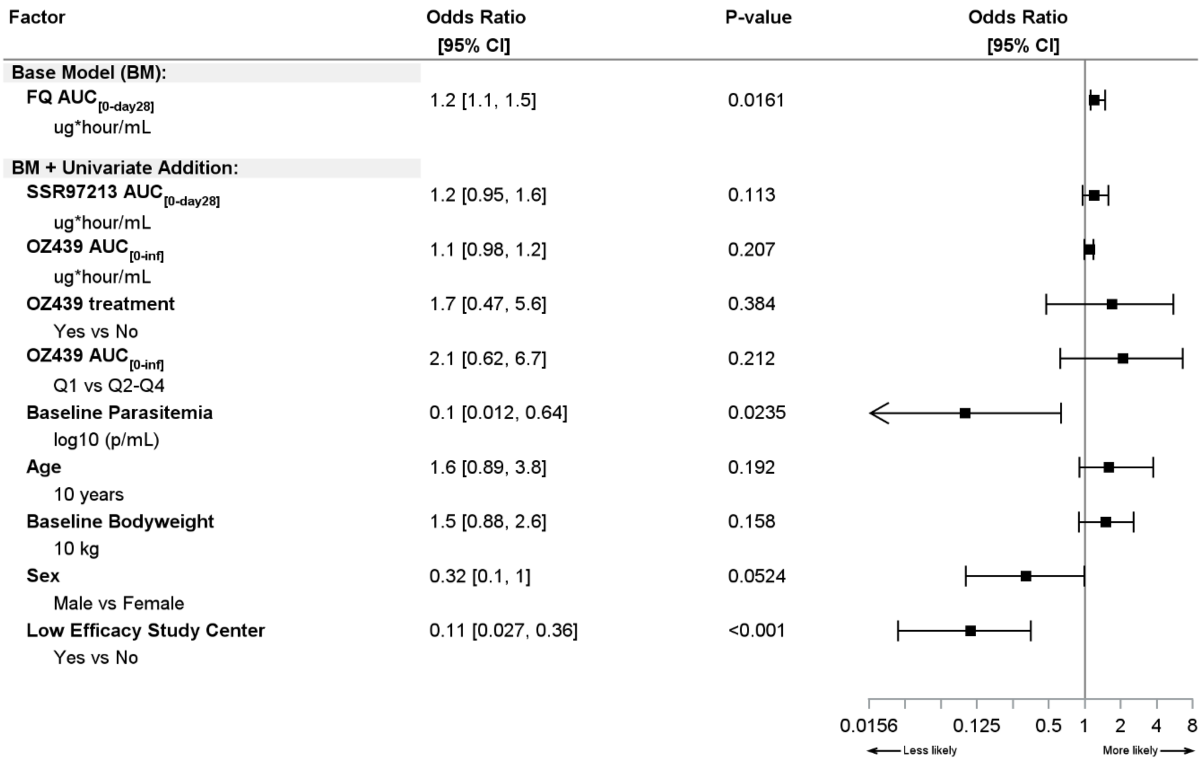

An odds ratio of greater than 1 represents a higher likelihood of achieving PCR-adjusted ACPR at Day 28.

Ferroquine $AUC_{[0\text{-}day28]}$, baseline parasitemia, and Low Efficacy Study Centers showed a statistically significant association with Day 28 PCR-adjusted ACPR in the univariate analysis ($P < 0.05$), whereas none of the artefenomel exposure covariates reached statistical significance. No effect of desmeythl-ferroquine $AUC_{[0\text{-}day28]}$, age, baseline body weight or sex could be identified. Since artefenomel exposure contribution to Day 28 PCR-adjusted ACPR was the primary objective, the artefenomel exposure covariate with the best outcome in the univariate analysis ($AUC_{[0\text{-}inf]}$, based on p-value) was taken forward to the backward elimination analysis. Table 1 summarizes the model parameters of the full model including the artefenomel $AUC_{[0\text{-}inf]}$ variable.

During the backward elimination, artefenomel $AUC_{[0\text{-}inf]}$ was not retained in the model and no meaningful interactions were identified for the final model.

Table 1 Model parameters of exposure–response for Day 28 PCR-adjusted ACPR full model

Parameter	Value	95%CI	*P* value
Intercept	22.06	6.35, 41.10	0.01
Ferroquine $AUC_{[0\text{-}day28]}$, µg*hour/mL	0.205	0.027, 0.410	0.034
Artefenomel $AUC_{[0\text{-}inf]}$, µg*hour/mL	0.067	-0.025, 0.205	0.245
Baseline Parasitemia, Log10 (p/mL)	-2.945	-5.559, -0.766	0.014
Low Efficacy Study Center, Yes vs No	-2.545	-4.125, -1.217	<0.001

The model that best fitted the data statistically indicated that Day 28 PCR-adjusted ACPR could be described as a function of ferroquine $AUC_{[0\text{-}day28]}$, baseline parasitemia and Low Efficacy Study Centers. Model predictions were compared to observed Day 28 PCR-adjusted ACPR across treatment groups and showed that the final model described the observed data adequately (shown in Figure 3).

Figure 3 Visual predictive checks of the best model for Day 28 PCR-corrected ACPR exposure–response analysis

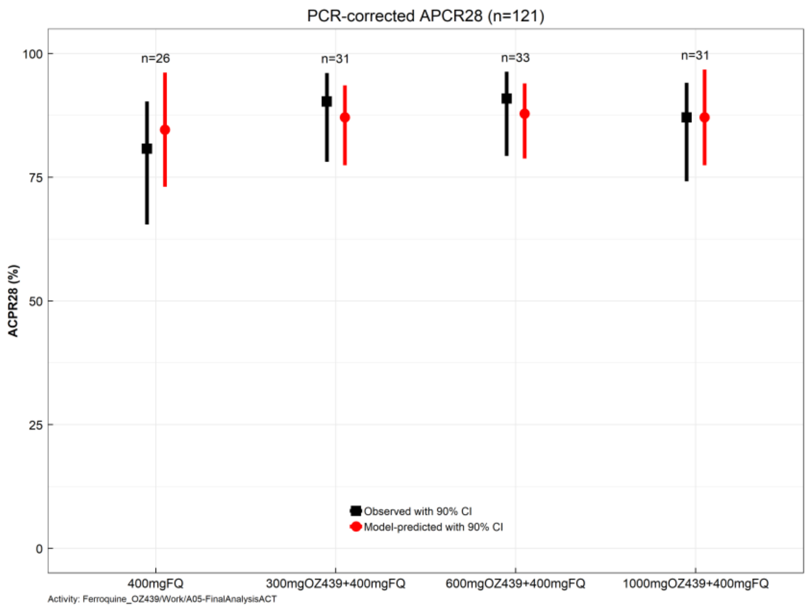

ACPR28: adequate clinical and parasitological response at Day 28.

A difference between the clinical trial simulations used to design this study and the final analysis was that the former (as well as the Ferroquine and Artefenomel in aduLts and Children with *Plasmodium*

falciparum malaria [FALCI] study analysis [Adoke, 2021]) used a model linking the concentrations at day 7 (C_{day7}) rather than AUC to clinical response. A post-hoc analysis did not identify a significant contribution of artefenomel C_{day7} on Day 28 PCR-adjusted ACPR either, which was not surprising considering the high correlation between AUC and C_{day7} after a single dose.

Exposure–response for Day 28 crude ACPR

The relationship between the estimated exposure of artefenomel and ferroquine and the response defined as Day 28 crude ACPR was evaluated using a logistic regression as described above. The same exposure variables and clinical and demographic covariates tested in the exposure–response analysis for Day 28 PCR-adjusted ACPR were tested for Day 28 crude ACPR.

Univariate analysis for Day 28 PCR-adjusted ACPR is shown in Figure 4.

Figure 4 Univariate analysis for Day 28 crude ACPR

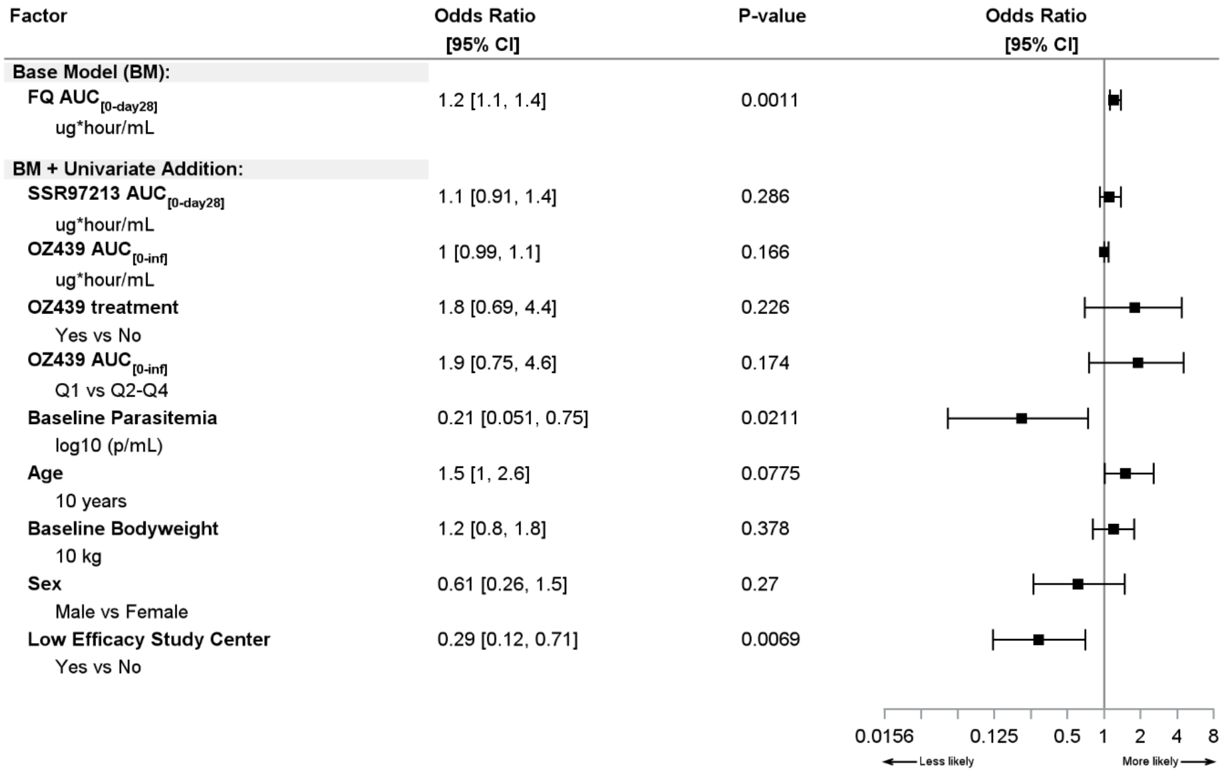

An odds ratio (OR) of greater than 1 represents a higher likelihood of achieving crude ACPR at Day 28.

Ferroquine $AUC_{[0\text{-}day28]}$, baseline parasitemia, and Low Efficacy Study Centers showed a statistically significant association with Day 28 crude ACPR in the univariate analysis ($P < 0.05$), whereas none of the artefenomel exposure covariates reached statistical significance. No effect of desmeythl-ferroquine $AUC_{[0\text{-}day28]}$, age, baseline body weight or sex could be identified. Since artefenomel exposure contribution to Day 28 crude ACPR was the question of interest, the artefenomel exposure covariate with the best outcome in the univariate analysis (artefenomel $AUC_{[0\text{-}inf]}$, based on *P* value) was taken

forward to the backward elimination analysis. Table 2 summarizes the model parameters of the full model including the artefenomel $AUC_{[0\text{-}inf]}$ variable.

Table 2 Model parameters of exposure–response for Day 28 crude ACPR full model

Parameter	Value	95%CI	P-value
Intercept	11.18	1.34, 22.05	0.03
ferroquine $AUC_{[0\text{-}day28]}$, µg*hour/mL	0.202	0.076, 0.345	0.003
artefenomel $AUC_{[0\text{-}inf]}$, µg*hour/mL	0.042	-0.016, 0.117	0.210
Baseline Parasitemia, Log10 (p/mL)	-1.649	-3.170, -0.269	0.025
Low Efficacy Study Center, Yes vs No	-1.299	-2.260, -0.374	0.007

During the backward elimination, artefenomel $AUC_{[0\text{-}inf]}$ was not retained in the model and no meaningful interactions were identified for the final model.

The model that best fitted the data statistically indicated that Day 28 crude ACPR could be described as a function of ferroquine $AUC_{[0\text{-}day28]}$, baseline parasitemia and Low Efficacy Study Centers. Model predictions were compared to observed Day 28 PCR-adjusted ACPR across treatment groups and showed that the final model described the observed data adequately (shown in Figure 4).

Figure 4 Visual predictive checks of the best model for Day 28 crude ACPR exposure–response analysis

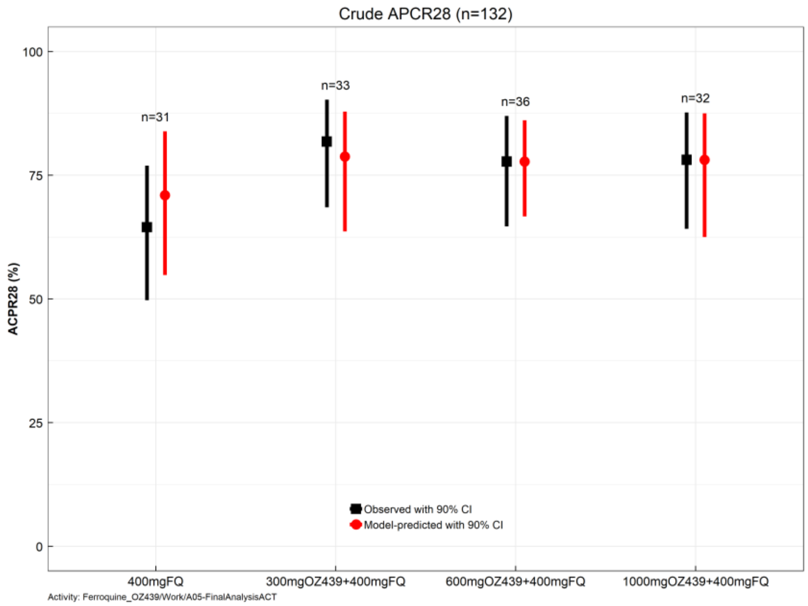

ACPR28: Day 28 adequate clinical and parasitological response.

References

Adoke Y, Zoleko-Manego R, Ouoba S, Tiono A, Kaguthi G, Bonzela JE, et al. A randomized, double-blind, phase 2b study to investigate the efficacy, safety, tolerability and pharmacokinetics of a single-dose regimen of ferroquine with artefenomel in adults and children with uncomplicated *Plasmodium falciparum* malaria. Malaria J. 2021; 20:222.

Modeling & Simulation in R – Supporting efficient model informed drug development with IQR tools. IntiQuan GmbH, Basel, Switzerland, 22 October 2020. https://iqrtools.intiquan.com/. Accessed 16 February 2021.
