# Supplementary material for: Randomized, open-label, phase 2a study to evaluate the contribution of artefenomel to the clinical and parasiticidal activity of artefenomel plus ferroquine in African patients with uncomplicated Plasmodium falciparum malaria
Source: Malar J. 2023 Jan 3;22:2. doi: 10.1186/s12936-022-04420-2 (PMC9809015; doi:10.1186/s12936-022-04420-2)
Supplement: Supplementary file 5 — Additional file 5. Supplementary tables and figures. [file 12936_2022_4420_MOESM5_ESM.pdf]

Additional file 5: Supplementary results

Contents

Table S1: Categorical and continuous information used for the univariate efficacy exposure–response analysis, artefenomel and ferroquine exposure, and Day 28 PCR-adjusted ACPR in the PK/PD efficacy population 2

Figure S1: Final exposure–response model for Day 28 PCR-adjusted ACPR (PK/PD efficacy population). 4

Figure S2: Full exposure–response model for Day 28 crude ACPR (PK/PD efficacy population). 5

Table S2: Day 28 PCR-adjusted and crude adequate clinical and parasitological response (ACPR) in the per-protocol (PP) and microbiological intention-to-treat (mITT) populations 6

Figure S3: Kaplan–Meier plot of elapsed time below the lower limit of quantification (LLQ) of parasitemia in the microbiological intention-to-treat population. 7

Table S3: Observed parasite clearance kinetics (mITT population) 8

Table S4: Pharmacokinetic (PK) parameters for ferroquine, desmethyl-ferroquine and artefenomel (PK population) 9

Table S5: All treatment emergent adverse events of any cause (safety population). 10

Table S6: Adverse events considered related to ferroquine administration (safety population). 12

Table S7: Adverse events considered related to artefenomel administration (safety population). 13

Table S8: Maximum changes from baseline in hematology and clinical chemistry parameters (safety population). 14

Figure S5: Patients with potentially clinically relevant changes in hematology parameters (safety population). 15

Figure S6: E-DISH of post-baseline peak alanine aminotransferase and peak total bilirubin during treatment (safety population). 16

Table S9: Maximum changes from baseline in vital signs (safety population). 17

Table S10: Summary of QT prolongation using Bazett's (QTcB) or Fridericia's (QTcF) formulae (safety population). 18

Table S1: Categorical and continuous information used for the univariate efficacy exposure–response analysis, artefenomel and ferroquine exposure, and Day 28 PCR-adjusted ACPR in the PK/PD efficacy population

Characteristic	Ferroquine 400 mg (N = 26)	Ferroquine 400 mg plus artefenomel: 300 mg (N = 31)	600 mg (N = 33)	1000 mg (N = 31)
Sex				
Female, n (%)	17 (65.4)	24 (77.4)	23 (69.7)	20 (64.5)
Male, n (%)	9 (34.6)	7 (22.6)	10 (30.3)	11 (35.5)
Country, n (%)				
Benin	4 (15.4)	4 (12.9)	4 (12.1)	4 (12.9)
Burkina Faso	15 (57.7)	16 (51.6)	16 (48.5)	16 (51.6)
Gabon	5 (19.2)	4 (12.9)	5 (15.2)	5 (16.1)
Kenya	0 (0)	2 (6.5)	3 (9.1)	2 (6.5)
Uganda	2 (7.7)	5 (16.1)	5 (15.2)	4 (12.9)
Low efficacy study center, n (%)				
No	17 (65.4)	23 (74.2)	24 (72.7)	22 (71.0)
Yes	9 (34.6)	8 (25.8)	9 (27.3)	9 (29.0)
Centre				
Cotonou	4 (15.4)	4 (12.9)	4 (12.1)	4 (12.9)
Libreville	3 (11.5)	1 (3.2)	2 (6.1)	2 (6.5)
Lambaréné	2 (7.7)	3 (9.7)	3 (9.1)	3 (9.7)
Kisumu	0 (0)	2 (6.5)	3 (9.1)	2 (6.5)
Kampala	2 (7.7)	5 (16.1)	5 (15.2)	4 (12.9)
Nanoro	6 (23.1)	6 (19.4)	7 (21.2)	7 (22.6)
Banfora	9 (34.6)	10 (32.3)	9 (27.3)	9 (29.0)
Artefenomel treatment				
No	26 (100)	0 (0)	0 (0)	0 (0)
Yes	0 (0)	31 (100)	33 (100)	31 (100)
Artefenomel exposure				
No	26 (100)	1 (3.23)	1 (3.03)	0 (0)
Yes	0 (0)	30 (96.8)	32 (97.0)	31 (100)
Vomit status				
No	25 (96.2)	29 (93.5)	28 (84.8)	23 (74.2)
Yes	1 (3.85)	2 (6.45)	5 (15.2)	8 (25.8)
Age, years	23.2 (12.4) [14–61]	22.1 (12.3) [14–64]	21.2 (8.79) [14–48]	22.1 (11.5) [14–67]

Body weight, kg	56 (11.3) [35.7–74.9]	54.3 (10.4) [37.0–86.0]	54.2 (11.8) [36.8–87.8]	57.9 (12.2) [38.0–80.2]
Baseline parasitemia, p/µL	19,500 (11,900) [3490–45,300]	19,200 (12,900) [3020–44300]	16,300 (13,200) [3120–48,100]	20,900 (13,900) [3760–50,600]
Parasitemia, $\log_{10}$ parasites/µL	4.2 (0.30) [3.54–4.66]	4.16 (0.36) [3.48–4.65]	4.07 (0.36) [3.49–4.68]	4.21 (0.34) [3.58–4.7]
Artefenomel C_{day7}, ng/mL	0 (0) [0–0]	1.24 (0.70) [0.033–2.53]	3.54 (2.68) [0.025–12.3]	7.06 (5.38) [0.176–23.6]
Artefenomel $AUC_{0–\infty}$, µg*h/mL	0 (0) [0–0]	4.25 (2.28) [0.14–10.3]	10.3 (7.6) [0.16–34.2]	18 (11.6) [1.2–44.7]
Ferroquine C_{day7}, ng/mL	17.8 (5.96) [8.29–31.2]	20 (6.92) [6.62–31.5]	21.5 (10.7) [6.78–50.2]	20.9 (11.7) [3.21–62.8]
Ferroquine $AUC_{0–d28}$, µg*h/mL	9.82 (3.45) [4.3–16.3]	11.2 (3.68) [3.84–16.9]	11.6 (5.29) [2.98–25.3]	11.9 (7.01) [2.49–38.9]
Desmethyl-ferroquine C_{day7}, ng/mL	18.6 (7.46) [9.04–35.4]	19.3 (8.65) [3.27–41.3]	19.2 (11.9) [3.67–58.0]	19.3 (13.5) [1.57–75.1]
Desmethyl-ferroquine $AUC_{0–d28}$, µg*h/mL	10 (3.9) [4.08–18.4]	10.4 (4.04) [2.38–20.3]	10.3 (5.61) [2.27–25.0]	10.5 (6.93) [1.72–39.1]
Day 28 PCR-adjusted ACPR, n/N (%) [95%CI]	21/26 (80.8) [62.1, 91.5]	28/31 (90.3) [75.1, 96.7]	30/33 (90.9) [76.4, 96.9]	27/31 (87.1) [71.1, 94.9]

Values are mean (standard deviation) [range] unless otherwise indicated.
C_{day7}, concentration on Day 7. $AUC_{0–d28}$, area under the curve from time 0 to Day 28; $AUC_{0–\infty}$, area under the curve from time 0 to infinity.

Figure S1: Final exposure–response model for Day 28 PCR-adjusted ACPR (PK/PD efficacy population).

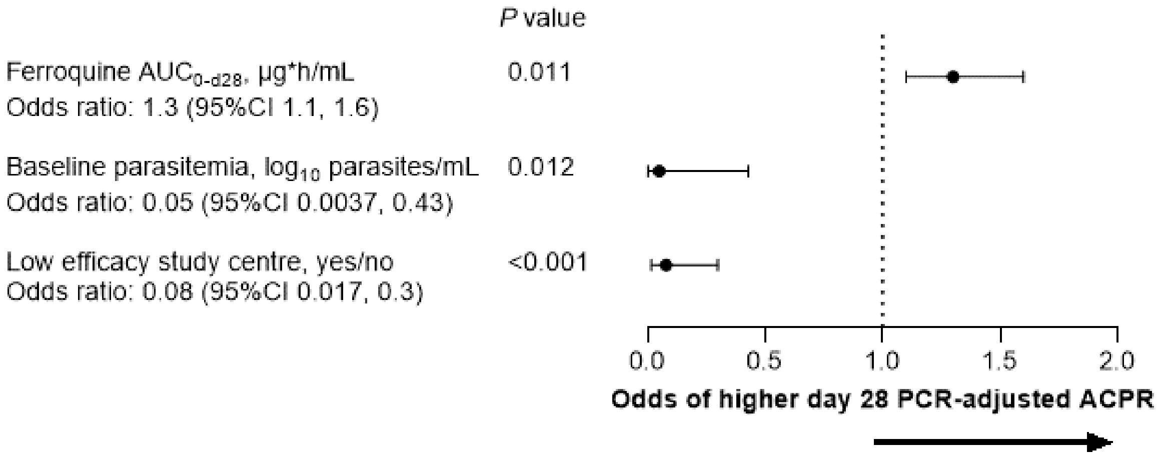

$AUC_{0–d28}$, area under the curve from time 0 to Day 28; $AUC_{0–\infty}$, area under the curve from time 0 to infinity; ACPR, adequate clinical and parasitological response.

Figure S2: Full exposure–response model for Day 28 crude ACPR (PK/PD efficacy population).

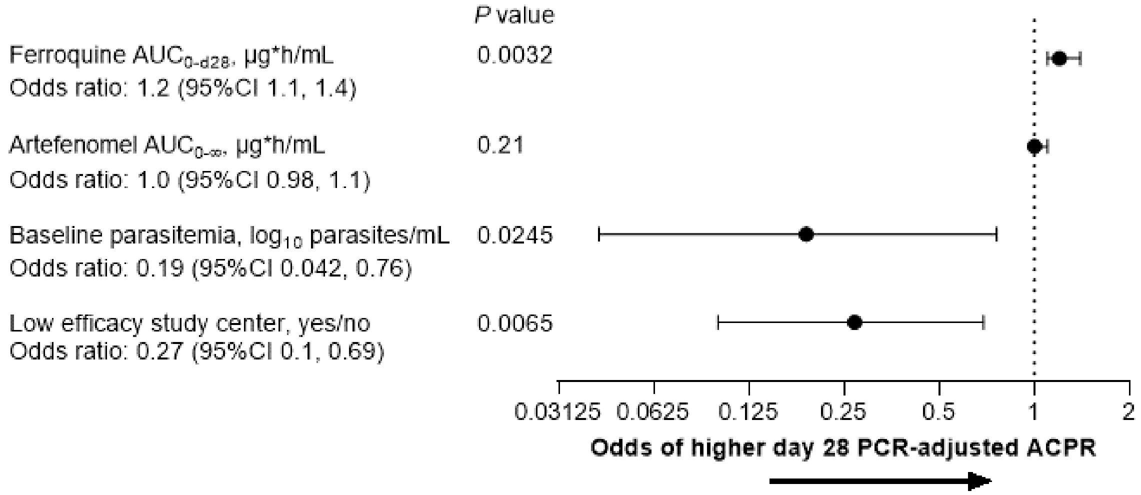

$AUC_{0–d28}$, area under the curve from time 0 to Day 28; $AUC_{0–\infty}$, area under the curve from time 0 to infinity; ACPR, adequate clinical and parasitological response.

Table S2: Day 28 PCR-adjusted and crude adequate clinical and parasitological response (ACPR) in the per-protocol (PP) and microbiological intention-to-treat (mITT) populations.

Outcome, n (%) [95%CI]	Ferroquine 400 mg	Ferroquine 400 mg plus artefenomel:		
		300 mg	600 mg	1000 mg
**PCR-adjusted (PP population)**	N = 31	N = 33	N = 36	N = 32
Number evaluable	26	31	33	31
Success	21 (80.8) [60.6, 93.4]	28 (90.3) [74.2, 98.0]	30 (90.9) [75.7, 98.1]	27 (87.1) [70.2, 96.4]
Early treatment failure	1 (3.8) [0.1, 19.6]	1 (3.2) [0.1, 16.7]	1 (3.0) [0.1, 15.8]	0 [0, 11.2]
Late clinical failure	1 (3.8) [0.1, 19.6]	0 [0, 11.2]	0 [0, 10.6]	2 (6.5) [0.8, 21.4]
Late parasitological failure	3 (11.5) [2.4, 30.2]	2 (6.5) [0.8, 21.4]	2 (6.1) [0.7, 20.2]	2 (6.5) [0.8, 21.4]
PCR undetermined	3	1	1	1
P. falciparum reinfection	2	1	2	0
**Crude (PP population)**	N = 31	N = 33	N = 36	N = 32
Number evaluable	31	33	36	32
Success	20 (64.5) [45.4, 80.8]	27 (81.8) [64.5. 93.0]	28 (77.8) [60.8, 89.9]	25 (78.1) [60.0, 90.7]
Early treatment failure	1 (3.2) [0.1, 16.7]	1 (3.0) [0.1, 15.8]	1 (2.8) [0.1, 14.5]	0 [0, 10.9]
Late clinical failure	2 (6.5) [0.8, 21.4]	0 [0, 10.6]	0 [0, 9.7]	3 (9.4) [2.0, 25.0]
Late parasitological failure	8 (25.8) [11.9, 44.6]	5 (15.2) [5.1, 31.9]	7 (19.4) [8.2, 36.0]	4 (12.5) [3.5, 29.0]
**PCR-adjusted (mITT population)**	N = 35	N = 36	N = 36	N = 33
Number evaluable	35	36	36	33
Success	21 (60.0) [42.1, 76.1]	28 (77.8) [60.8, 89.9]	30 (83.3) [67.2, 93.6]	27 (81.8) [64.5, 93.0]
Early treatment failure	1 (2.9) [0.1, 14.9]	1 (2.8) [0.1, 14.5]	1 (2.8) [0.1, 14.5]	0 [0, 10.6]
Late clinical failure	2 (5.7) [0.7, 19.2]	0 [0, 9.7]	0 [0, 9.7]	3 (9.1) [1.9, 24.3]
Late parasitological failure	7 (20.0) [8.4, 36.9]	4 (11.1) [3.1, 26.1]	5 (13.9) [4.7, 29.5]	2 (6.1) [0.7, 20.2]
**Crude (mITT population)**	N = 35	N = 36	N = 36	N = 33
Number evaluable	35	36	36	33
Success	20 (57.1) [39.4, 73.7]	27 (75.0) [57.8, 87.9]	28 (77.8) [60.8, 89.9]	25 (75.8) [57.7. 88.9]
Early treatment failure	1 (2.9) [0.1, 14.9]	1 (2.8) [0.1, 14.5]	1 (2.8) [0.1, 14.5]	0 [0, 10.6]
Late clinical failure	2 (5.7) [0.7, 19.2]	0 [0, 9.7]	0 [0, 9.7]	3 (9.1) [1.9, 24.3]
Late parasitological failure	8 (22.9) [10.4, 40.1]	5 (13.9) [4.7, 29.5]	7 (19.4) [8.2, 36.0]	4 (12.1) [3.4, 28.2]

Figure S3: Kaplan–Meier plot of elapsed time below the lower limit of quantification (LLQ) of parasitemia in the microbiological intention-to-treat population.

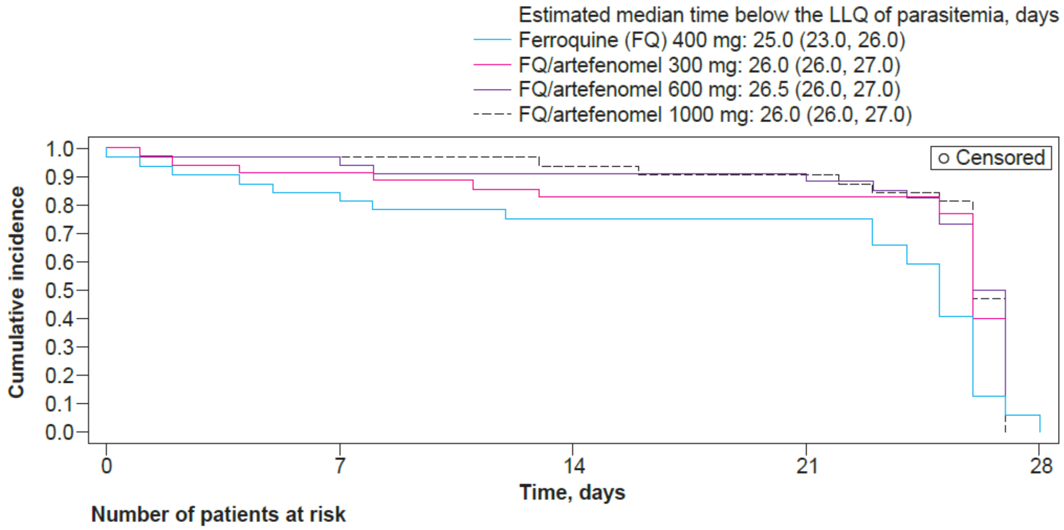

Number of patients at risk

	0	7	14	21	28
Ferroquine (FQ) 400 mg	32	27	24	24	0
FQ/artefenomel 300 mg	35	32	29	29	2
FQ/artefenomel 600 mg	34	33	31	31	2
FQ/artefenomel 1000 mg	32	31	30	29	0

Figure S4: Kaplan–Meier plot of time to confirmed fever clearance in the microbiological intention-to-treat population.

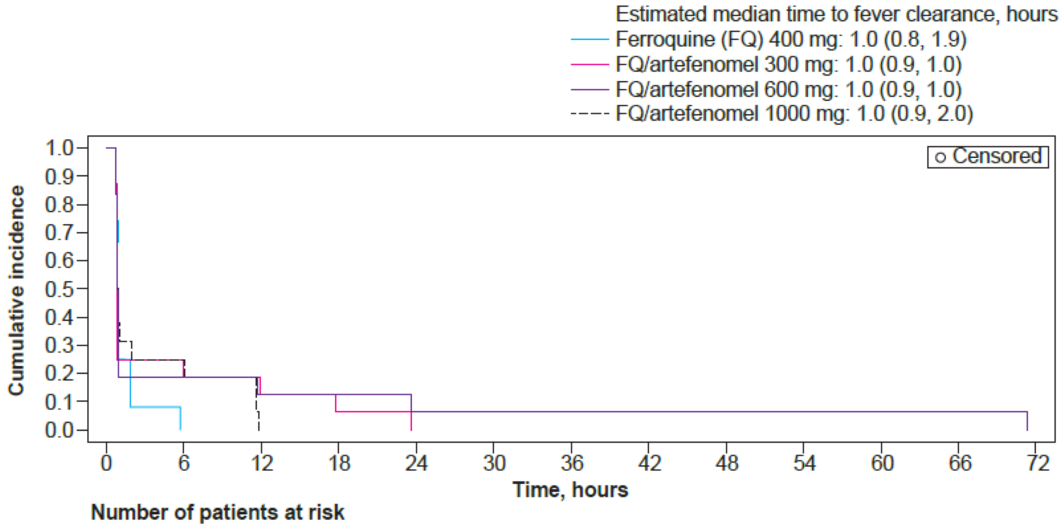

Number of patients at risk

	0	6	12	18	24	30	36	42	48	54	60	66	72
Ferroquine (FQ) 400 mg	12	1	0	0	0	0	0	0	0	0	0	0	0
FQ/artefenomel 300 mg	16	4	3	1	1	0	0	0	0	0	0	0	0
FQ/artefenomel 600 mg	16	3	3	2	2	1	1	1	1	1	1	1	1
FQ/artefenomel 1000 mg	16	4	3	0	0	0	0	0	0	0	0	0	0

Table S3: Observed parasite clearance kinetics (mITT population)

Endpoint	Ferroquine 400 mg (N = 35)	Ferroquine 400 mg plus artefenomel:		
		300 mg (N = 36)	600 mg (N = 36)	1000 mg (N = 33)
Observed PRR24	n = 34	n = 36	n = 36	n = 33
Mean (SD)	638.4 (2013.9)	5921.2 (11,566.2)	5715.0 (9364.9)	7929.7 (12,675.8)
[range]	[0.45 to 9417]	[1.5 to 45,061.0]	[0.08 to 40,105.0]	[1.2 to 48,411.0]
Median (Q1, Q3)	17.8 (5.0, 53.9)	405.2 (134.5, 3843.5)	814.1 (243.2, 6858.5)	1279.3 (158.5, 9962.0)
Observed PRR48	n = 34	n = 36	n = 36	n = 33
Mean (SD)	9709.1 (13,987.7)	17,594.7 (13,522.0)	13,120.4 (13,182.3)	19,328.1 (14,392.8)
[range]	[0.87 to 46,076.0]	[1.0 to 45,061.0]	[0.6 to 54,141.0]	[5.1 to 51,862.0]
Median (Q1, Q3)	1472.8 (101.5, 14,419.0)	15,689.0 (5571.5, 29,394.5)	8280.0 (4018.0, 16,897.0)	18,685.0 (7675.0, 30,294.0)
Observed PRR72	n = 33	n = 35	n = 35	n = 32
Mean (SD)	15,655.4 (12,806.5)	18,069.6 (13409.1)	14,561.4 (13,657.1)	19,125.7 (14,516.8)
[range]	[8.1 to 46,076.0]	[1.4 to 45,061.0]	[4.6 to 54,141.0]	[27.8 to 51,862.0]
Median (Q1, Q3)	13,156.0 (6855.0, 23,838.0)	16,213.0 (6167.0, 29,654.0)	9873.0 (4090.0, 18,624.0)	15,273.0 (7577.5, 30,998.5)

Table S4: Pharmacokinetic (PK) parameters for ferroquine, desmethyl-ferroquine and artefenomel (PK population)

Analyte Parameter	Ferroquine 400 mg (N = 35)	Ferroquine 400 mg plus artefenomel: 300 mg (N = 36)	600 mg (N = 36)	1000 mg (N = 33)
Ferroquine				
C_{max}, ng/mL	77.31 (61)	83.39 (90)	72.64 (88)	82.59 (93)
C_{d7}, ng/ml	15.73 (43)	18.73 (41)	18.69 (52)	17.8 (59)
$AUC_{0–d28}$, µg*h/mL	8.81 (41)	10.59 (40)	10.13 (51)	10.17 (55)
$AUC_{0–\infty}$, µg*h/mL	15.69 (47)	18.5 (43)	16.3 (63)	17.13 (58)
Desmethyl-ferroquine				
C_{max}, ng/mL	23.03 (64)	23.73 (81)	19.14 (99)	19.6 (76)
C_{day7}, ng/ml	15.63 (50)	16.73 (66)	15.2 (78)	14.84 (84)
$AUC_{0–d28}$, µg*h/mL	8.65 (47)	9.43 (56)	8.48 (68)	8.48 (70)
$AUC_{0–\infty}$, µg*h/mL	19.54 (53)	21.17 (49)	17.94 (69)	18.58 (67)
Artefenomel				
C_{max}, ng/mL	NA	292.1 (161)	488.3 (232)	899.8 (77)
C_{d7}, ng/ml	NA	0.92 (104)	2.15 (202)	4.42 (161)
$AUC_{0–\infty}$, µg*h/mL	NA	3.32 (107)	6.46 (181)	12.94 (112)

Values are geometric mean (% coefficient of variation). NA, not applicable.
$AUC_{0–d28}$: area under the curve from time 0 to Day 28; $AUC_{0–\infty}$: area under the curve from time 0 to infinity; C_{d7}: concentration at Day 7 post-dose; C_{max}: maximal observed concentration.

Table S5: All treatment emergent adverse events of any cause (safety population).

System organ class, preferred term, n (%)	Ferroquine 400 mg (N = 35)	Ferroquine plus artefenomel:		
		300 mg (N = 36)	600 mg (N = 36)	1000 mg (N = 33)
At least one adverse event	18 (51.4)	21 (58.3)	24 (66.7)	24 (72.7)
Mild severity	10 (28.6)	9 (25.0)	12 (33.3)	14 (42.4)
Moderate severity	8 (22.9)	12 (33.3)	12 (33.3)	10 (30.3)
Infections and infestations	9 (25.7)	11 (30.6)	12 (33.3)	7 (21.2)
Malaria	7 (20.0)	6 (16.7)	8 (22.2)	6 (18.2)
Rhinitis	0	1 (2.8)	2 (5.6)	2 (6.1)
Bronchitis	1 (2.9)	1 (2.8)	1 (2.8)	1 (3.0)
Oral herpes	0	0	0	1 (3.0)
Schistosomiasis	0	1 (2.8)	0	1 (3.0)
Ear infection	0	1 (2.8)	0	0
Furuncle	0	1 (2.8)	0	0
Gastroenteritis	0	1 (2.8)	0	0
Influenza	1 (2.9)	1 (2.8)	1 (2.8)	0
Parasitic gastroenteritis	0	0	1 (2.8)	0
Schistosomiasis bladder	1 (2.9)	0	0	0
Sinusitis	1 (2.9)	0	0	0
Blood and lymphatic system disorders	3 (8.6)	2 (5.6)	2 (5.6)	1 (3.0)
Neutropenia	1 (2.9)	0	1 (2.8)	1 (3.0)
Anemia	0	2 (5.6)	1 (2.8)	0
Thrombocytopenia	2 (5.7)	0	0	0
Metabolism and nutrition disorders	0	1 (2.8)	0	0
Decreased appetite	0	1 (2.8)	0	0
Psychiatric disorders	1 (2.9)	0	0	0
Conversion disorder	1 (2.9)	0	0	0
Nervous system disorders	3 (8.6)	6 (16.7)	3 (8.3)	10 (30.3)
Headache	3 (8.6)	6 (16.7)	1 (2.8)	7 (21.2)
Dizziness	0	0	2 (5.6)	4 (12.1)
Migraine	0	0	1 (2.8)	0
Eye disorders	0	0	1 (2.8)	0
Conjunctival hyperemia	0	0	1 (2.8)	0
Ear and labyrinth disorders	0	2 (5.6)	0	0
Hypoacusis	0	1 (2.8)	0	0
Vertigo	0	1 (2.8)	0	0
Cardiac disorders	1 (2.9)	0	1 (2.8)	0
Palpitations	1 (2.9)	0	1 (2.8)	0
Vascular disorders	1 (2.9)	0	0	0
Phlebitis	1 (2.9)	0	0	0
Respiratory, thoracic, and mediastinal disorders	0	1 (2.8)	0	1 (3.0)
Cough	0	1 (2.8)	0	1 (3.0)
Gastrointestinal disorders	5 (14.3)	8 (22.2)	10 (27.8)	11 (33.3)
Vomiting	3 (8.6)	3 (8.3)	5 (13.9)	10 (30.3)
Abdominal pain	1 (2.9)	4 (11.1)	0	2 (6.1)
Diarrhea	0	1 (2.8)	0	2 (6.1)
Nausea	1 (2.9)	1 (2.8)	2 (5.6)	2 (6.1)

System organ class, preferred term, n (%)	Ferroquine 400 mg (N = 35)	Ferroquine plus artefenomel:		
		300 mg (N = 36)	600 mg (N = 36)	1000 mg (N = 33)
Enteritis	0	0	1 (2.8)	1 (3.0)
Constipation	0	2 (5.6)	0	0
Dyspepsia	0	0	1 (2.8)	0
Food poisoning	0	0	1 (2.8)	0
Gastritis	1 (2.9)	0	0	0
Toothache	0	0	1 (2.8)	0
Skin and subcutaneous tissue disorders	2 (5.7)	1 (2.8)	0	1 (3.0)
Pruritis	2 (5.7)	0	0	1 (3.0)
Rash vesicular	0	1 (2.8)	0	0
Musculoskeletal disorders	0	0	0	1 (3.0)
Myalgia	0	0	0	1 (3.0)
Renal and urinary disorders	1 (2.9)	0	1 (2.8)	0
Hematuria	1 (2.9)	0	1 (2.8)	0
Pregnancy, puerperium, and perinatal disorders	0	1 (2.8)	0	0
Pregnancy	0	1 (2.8)	0	0
General disorders and administration site conditions	2 (5.7)	2 (5.6)	2 (5.6)	4 (12.1)
Pyrexia	0	2 (5.6)	1 (2.8)	4 (12.1)
Asthenia	0	0	0	1 (3.0)
Chills	1 (2.9)	0	1 (2.8)	0
Fatigue	0	1 (2.8)	0	0
Treatment failure	1 (2.9)	0	0	0
Investigations	2 (5.7)	2 (5.6)	4 (11.1)	4 (12.1)
ALT increased	0	0	0	1 (3.0)
Blook CPK increased	0	1 (2.8)	1 (2.8)	1 (3.0)
ECG PR prolongation	0	0	0	1 (3.0)
Neutrophil decreased	0	1 (2.8)	1 (2.8)	1 (3.0)
Creatinine real clearance decreased	1 (2.9)	0	0	0
GFR decreased	0	0	1 (2.8)	0
Hemoglobin decreased	0	0	1 (2.8)	0
Specific gravity urine increased	1 (2.9)	0	0	0
WBC count decreased	0	0	1 (2.8)	0
pH urine	0	0	1 (2.8)	0
Injury, poisoning, and procedural complications	0	1 (2.8)	1 (2.8)	1 (3.0)
Thermal burn	0	0	0	1 (3.0)
Limb injury	0	0	1 (2.8)	0
Nasal injury	0	1 (2.8)	0	0

Table S6: Adverse events considered related to ferroquine administration (safety population).

System organ class, preferred term, n (%)	Ferroquine 400 mg (N = 35)	Ferroquine plus artefenomel:		
		300 mg (N = 36)	600 mg (N = 36)	1000 mg (N = 33)
At least one adverse event	5 (14.3)	2 (5.6)	1 (2.8)	3 (9.1)
Blood and lymphatic system disorders	1 (2.9)	0	0	0
Neutropenia	1 (2.9)	0	0	0
Nervous system disorders	0	0	0	1 (3.0)
Dizziness	0	0	0	1 (3.0)
Gastrointestinal disorders	1 (2.9)	1 (2.8)	1 (2.8)	2 (6.1)
Vomiting	1 (2.9)	1 (2.8)	1 (2.8)	2 (6.1)
Nausea	0	0	0	1 (3.0)
Skin and subcutaneous tissue disorders	1 (2.9)	1 (2.8)	0	0
Pruritis	1 (2.9)	0	0	0
Rash vesicular	0	1 (2.8)	0	0
Renal and urinary disorders	1 (2.9)	0	0	0
Hematuria	1 (2.9)	0	0	0
Investigations	1 (2.9)	0	0	1 (3.0)
ALT increased	0	0	0	1 (3.0)
Creatinine renal clearance decreased	1 (2.9)	0	0	0

ALT, alanine aminotransferase.
Relatedness was assigned based on the opinion of the investigator.

Table S7: Adverse events considered related to artefenomel administration (safety population).

System organ class, preferred term, n (%)	Ferroquine 400 mg (N = 35)	Ferroquine plus artefenomel:		
		300 mg (N = 36)	600 mg (N = 36)	1000 mg (N = 33)
At least one adverse event	2 (5.7)	3 (8.3)	5 (13.9)	9 (27.3)
Nervous system disorders	0	0	0	1 (3.0)
Dizziness	0	0	0	1 (3.0)
Gastrointestinal disorders	1 (2.9)	2 (5.6)	5 (13.9)	8 (24.2)
Vomiting	1 (2.9)	2 (5.6)	3 (8.3)	8 (24.2)
Nausea	0	0	2 (5.6)	2 (6.1)
Skin and subcutaneous tissue disorders	0	1 (2.8)	0	0
Rash vesicular	0	1 (2.8)	0	0
Investigations	1 (2.9)	0	0	1 (3.0)
ALT increased	0	0	0	1 (3.0)
Creatinine renal clearance decreased	1 (2.9)	0	0	0

ALT, alanine aminotransferase.
Relatedness was assigned based on the opinion of the investigator.

Table S8: Maximum changes from baseline in hematology and clinical chemistry parameters (safety population).

Parameter	Ferroquine 400 mg (N = 35)		Ferroquine plus artefenomel: 300 mg (N = 36)		600 mg (N = 36)		1000 mg (N = 33)	
	n	Mean (SD)	n	Mean (SD)	n	Mean (SD)	n	Mean (SD)
Basophils, 10^9/L	15	−0.0027 (0.014)	19	0.015 (0.053)	18	−0.0039 (0.032)	17	0.0062 (0.035)
Eosinophils, 10^9/L	14	0.39 (0.76)	19	0.32 (0.56)	18	0.26 (0.38)	17	0.19 (0.43)
Erythrocytes, 10^{12}/L	34	−0.38 (0.59)	36	−0.44 (0.42)	36	−0.28 (0.55)	33	−0.39 (0.43)
Hematocrit, v/v	34	−0.034 (0.029)	36	−0.035 (0.050)	36	−0.035 (0.047)	33	−0.036 (0.037)
Hemoglobin, g/L	34	−10.4 (13.91)	36	−10.2 (13.16)	36	−11.7 (10.38)	33	−12.1 (11.00)
Leukocytes, 10^9/L	34	−1.47 (1.48)	36	−1.72 (2.87)	36	−0.74 (2.81)	33	−1.74 (1.70)
Lymphocytes, 10^9/L	15	1.02 (0.84)	19	0.88 (0.67)	18	0.85 (0.75)	17	1.02 (0.74)
Monocytes, 10^9/L	15	0.13 (0.72)	19	−0.28 (0.36)	18	−0.094 (0.34)	17	−0.044 (0.45)
Neutrophils, 10^9/L	15	−2.47 (1.54)	19	−3.12 (2.91)	18	−1.03 (3.27)	17	−2.53 (1.75)
Platelets, 10^9/L	34	−5.1 (50.76)	36	1.3 (43.14)	36	4.6 (49.08)	33	11.2 (61.74)
Reticulocytes, 10^9/L	30	15.99 (43.91)	32	6.76 (35.08)	31	6.73 (31.20)	29	1.48 (32.02)
Alanine aminotransferase, IU/L	34	2.48 (12.32)	36	−0.34 (11.46)	36	6.81 (15.01)	33	5.61 (14.92)
Albumin, g/L	34	−1.06 (7.47)	36	1.67 (5.99)	36	−0.19 (4.48)	33	0.22 (5.53)
Alkaline phosphatase, IU/L	34	28.72 (46.20)	36	19.43 (43.39)	36	11.55 (54.54)	33	28.46 (81.94)
Aspartate aminotransferase, IU/L	34	3.19 (14.66)	36	−0.99 (17.88)	36	3.53 (12.55)	33	2.18 (10.46)
Bilirubin, µmol/L	34	−3.35 (9.44)	36	−0.43 (19.10)	36	−1.69 (17.97)	33	0.15 (14.60)
Calcium, mmol/L	1	0.14 (NC)	2	0.15 (0.07)	0	NC	1	−0.11 (NC)
Creatine kinase, IU/L	34	143.12 (297.38)	36	113.52 (261.47)	36	190.26 (310.32)	33	121.34 (162.17)
Creatinine, µmol/L	34	−2.95 (19.10)	36	−13.56 (14.36)	36	−9.33 (17.64)	33	−13.31 (13.31)
Creatinine clearance, mL/min	34	−6.41 (31.39)	36	7.27 (35.57)	36	3.73 (33.82)	33	9.89 (29.86)
Direct bilirubin, µmol/L	22	−3.18 (2.27)	27	−2.52 (3.81)	28	−3.12 (2.71)	24	−2.15 (2.28)
Glucose, mmol/L	34	−0.35 (1.55)	36	−0.98 (1.84)	36	−0.06 (2.25)	33	0.14 (2.44)
Haptoglobin, g/L	31	−0.26 (0.37)	34	−0.18 (0.49)	34	−0.23 (0.49)	31	−0.28 (0.68)
Lactate dehydrogenase, IU/L	33	−25.50 (79.41)	35	−24.42 (105.73)	36	−11.55 (106.13)	33	−7.25 (106.76)
Magnesium, mmol/L	1	0.06 (NC)	2	− 0.05 (0.30)	0	NC	1	0.47 (NC)
Potassium, mmol/L	34	0.079 (0.85)	36	−0.11 (0.42)	36	0.10 (1.04)	33	−0.012 (0.69)
Sodium, mmol/L	34	−1.63 (5.27)	36	−0.55 (3.63)	36	−0.72 (4.25)	33	−0.69 (4.25)
Urea, mmol/L	34	−1.19 (1.47)	36	−1.58 (1.67)	36	−0.95 (1.29)	33	−1.34 (1.39)

NC, not calculated.

Figure S5: Patients with potentially clinically relevant changes in hematology parameters (safety population).

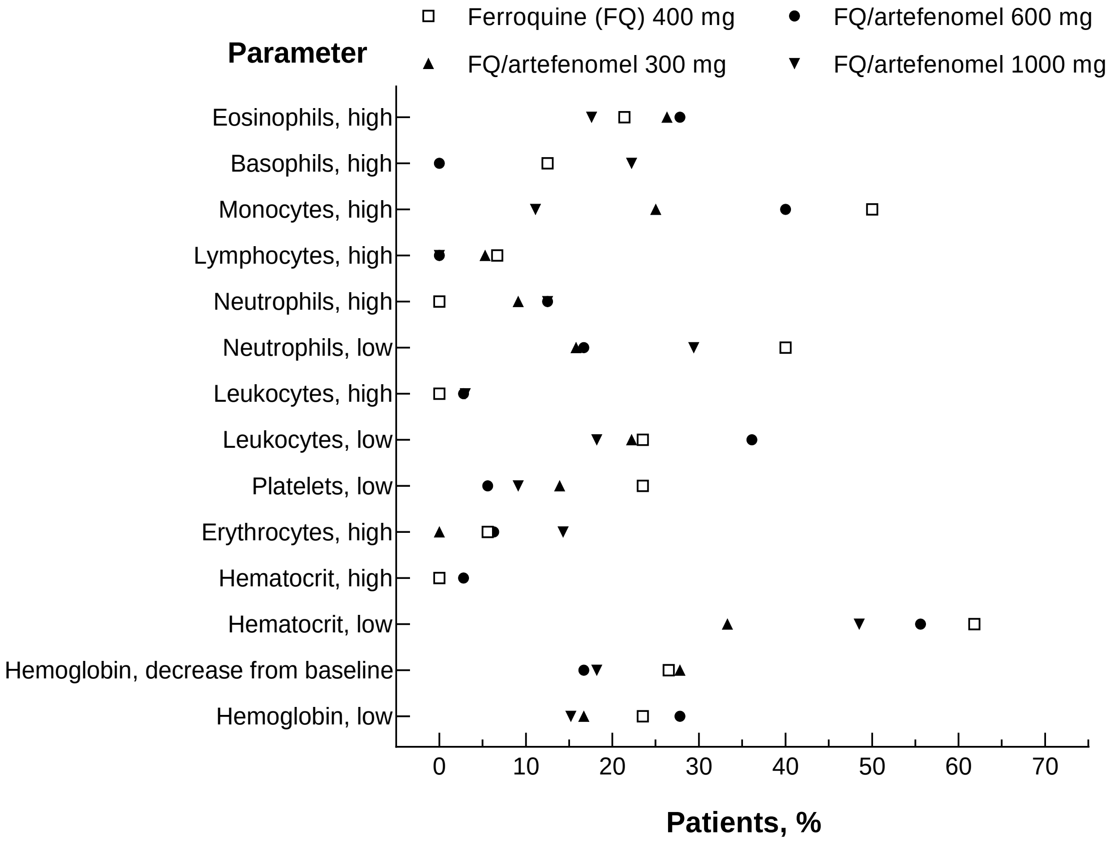

Figure S6: E-DISH of post-baseline peak alanine aminotransferase and peak total bilirubin during treatment (safety population).

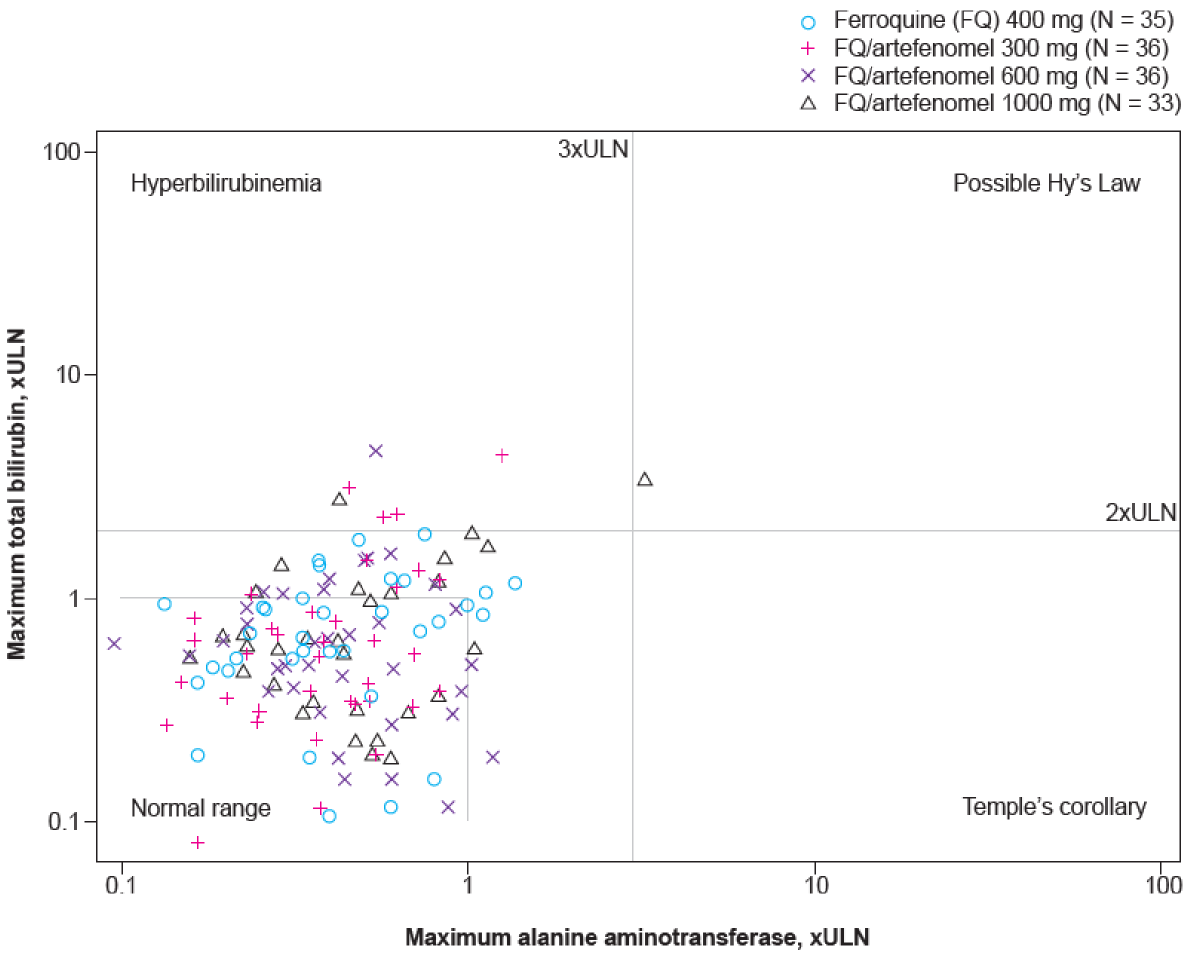

Table S9: Maximum changes from baseline in vital signs (safety population).

Parameter	Ferroquine 400 mg (N = 35)		Ferroquine plus artefenomel:					
			300 mg (N = 36)		600 mg (N = 36)		1000 mg (N = 33)	
	n	Mean (SD)	n	Mean (SD)	n	Mean (SD)	n	Mean (SD)
Heart rate, beats/min	19	−17.95 (15.45)	19	−8.21 (28.29)	16	−13.63 (19.02)	21	−16.10 (15.87)
Systolic blood pressure, mmHg	35	−4.4 (12.36)	36	−5.8 (12.96)	36	−0.9 (16.23)	33	−3.7 (18.46)
Diastolic blood pressure, mmHg	35	−9.6 (9.60)	36	−6.7 (11.20)	36	−6.1 (11.25)	33	−10.4 (10.52)

Table S10: Summary of QT prolongation using Bazett's (QTcB) or Fridericia's (QTcF) formulae (safety population).

Parameter n/N (%)	Category	Ferroquine 400 mg (N = 35)	Ferroquine plus artefenomel: 300 mg (N = 36)	600 mg (N = 36)	1000 mg (N = 33)
Highest QTcB, msec	>450	7/35 (20.0)	4/36 (11.1)	11/36 (30.6)	8/33 (24.2)
	>480	0	0	1/36 (2.8)	1/33 (3.0)
	>500	0	0	0	0
QTcB change from baseline, msec	>30	1/35 (2.9)	0	4/35 (11.4)	6/33 (18.2)
	>60	0	0	0	0
Highest QTcF, msec	>450	1/35 (2.9)	0	5/36 (13.9)	2/33 (6.1)
	>480	0	0	0	0
	>500	0	0	0	0
QTcF change from baseline, msec	>30	6/35 (17.1)	5/36 (13.9)	8/35 (22.9)	12/33 (36.4)
	>60	0	0	0	0
